# Structure–Activity Relationship of Metabolic Sialic Acid Inhibitors and Labeling Reagents

**DOI:** 10.1021/acschembio.1c00868

**Published:** 2022-02-18

**Authors:** Sam J. Moons, Emiel Rossing, Mathilde A. C. H. Janssen, Torben Heise, Christian Büll, Gosse J. Adema, Thomas J. Boltje

**Affiliations:** † Cluster of Molecular Chemistry, Institue for Molecules and Materials, Radboud University Nijmegen, Nijmegen 6525 AJ, The Netherlands; ‡ Department of Biomolecular Chemistry, Institute for Molecules and Materials, Radboud University Nijmegen, Nijmegen 6525 GA, The Netherlands; § Radiotherapy & OncoImmunology Laboratory, Department of Radiation Oncology, Radboud University Medical Center, Nijmegen 6525 GA, The Netherlands

## Abstract

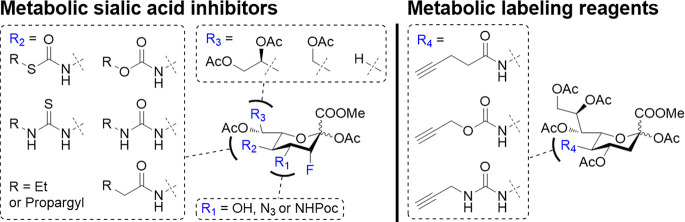

Sialic acids cap the glycans of cell surface glycoproteins and glycolipids. They are involved in a multitude of biological processes, and aberrant sialic acid expression is associated with several pathologies, such as cancer. Strategies to interfere with the sialic acid biosynthesis can potentially be used for anticancer therapy. One well-known class of sialylation inhibitors is peracetylated 3-fluorosialic acids. We synthesized 3-fluorosialic acid derivatives modified at the C-4, C-5, C-8, and C-9 position and tested their inhibitory potency in vitro. Modifications at C-5 lead to increased inhibition, compared to the natural acetamide at this position. These structure–activity relationships could also be applied to improve the efficiency of sialic acid metabolic labeling reagents by modification of the C-5 position. Hence, these results improve our understanding of the structure–activity relationships of sialic acid glycomimetics and their metabolic processing.

## Introduction

Sialic acids are nine-carbon sugars abundantly expressed at the termini of mammalian glycans on membrane-bound and secreted glycoproteins and glycolipids. The sialic acid family contains >80 chemically distinct members that are related to the nonulosonic acids, nine-carbon backbone α-keto sugars that are widely found in nature.^1,2^ Mammalian cells can produce sialic acids *via* a *de novo* biosynthesis pathway starting from *N*-acetylmannosamine (ManNAc) in a three-step enzymatic process.^3^ Subsequently, sialic acids are converted into CMP-sialic acids by CMP-sialic acid synthase (CMAS). CMP-sialic acids are transported into the Golgi apparatus where they are utilized as a sialyl donor by 20 sialyltransferase isoenzymes which install sialic acids *via* distinct glycosidic linkages (α2-3, α2-6, or α2-8) on various glycoconjugates (*N*/*O*-glycans, glycolipids) giving rise to so-called sialoglycans. Cell surface sialic acids are important modulators of a myriad of biological processes such as the binding and transport of ions, enhancing the viscosity of mucins, regulating glycoprotein half-life, and interacting with sialic acid binding proteins.^4^ Sialoglycans are recognized by sialic acid-binding immunoglobulin-like lectins (Siglecs), a family of immunoregulatory receptors,^5^ and selectins that mediate the trafficking of immune cells. Sialic acids therefore play an important role in modulating immune activity.^6^

Cancers of various origins display altered cell surface glycosylation with the overexpression of sialic acid as a frequently observed feature. The overexpression of sialic acid in cancer arises from increased metabolic flux of the sialic acid pathway, increased sialyltransferase expression, and a decrease in sialidase expression. Cancer hypersialylation is associated with resistance to radio- and chemotherapy, apoptotic evasion, cancer progression, and metastasis and the induction of an immunosuppressive tumor microenvironment hence leading to aggressive and invasive forms of cancer with a poor prognosis.^7^ Blocking aberrant sialylation in cancer may therefore represent a promising therapeutic strategy.^8^ Bacterial sialidases have been investigated in clinical trials to reduce cancer sialylation albeit with limited success. Bacterial sialidases are often contaminated,^9,10^ immunogenic, and known to stay bound after hydrolysis^11−13^ and their effects are short-lived as the sialic acid biosynthesis rapidly restores sialic acid levels.^14,15^ Recently, this approach has been revisited utilizing human sialidase–antibody constructs.^16^ An alternative strategy relies on the use of small-molecule inhibitors of sialoglycan biosynthesis. Paulson et al. reported a cell-permeable metabolic inhibitor of sialylation based on peracetylated 3-fluorosialic acid (P-SiaFNAc).^17^ Upon entering the cell, P-SiaFNAc is deacetylated by esterases, and SiaFNAc is converted to CMP-SiaFNAc by CMAS.^17−19^ CMP-SiaFNAc has been shown to act as a competitive inhibitor of sialyltransferase enzymes and leads to feedback inhibition of the *de novo* biosynthesis pathway. We and others have shown that P-SiaFNAc inhibits sialylation with high specificity in cancer cells resulting in reduced tumor growth and metastasis.^17,20−22^ The use of P-SiaFNAc has also significantly contributed to the understanding of how desialylation leads to potentiation of the immune response.^23,24^ By taking advantage of the substrate tolerance at C-5 of sialic acid, we have recently developed more potent analogues of P-SiaFNAc.^3,25^ We found that C-5 carbamate derivatives of P-SiaFNAc significantly enhanced inhibitory potency in vitro. The increased inhibitory potency was attributed to increased metabolic flux toward the active CMP derivative giving rise to higher intracellular concentrations of the active inhibitor. In addition, we reported a similar difference in the efficiency of metabolic labeling of C-5-modified sialic acids containing a C-5 propargyloxycarbonyl (Poc) with respect to the azidoacetyl (Az) derivative. Also, in this case, the carbamate derivative (Poc) was more effective than the amide (Az) derivative, although the difference in the chemical reporter group (alkyne *vs* azide) prevented a direct comparison.^26^

**Figure 1:**
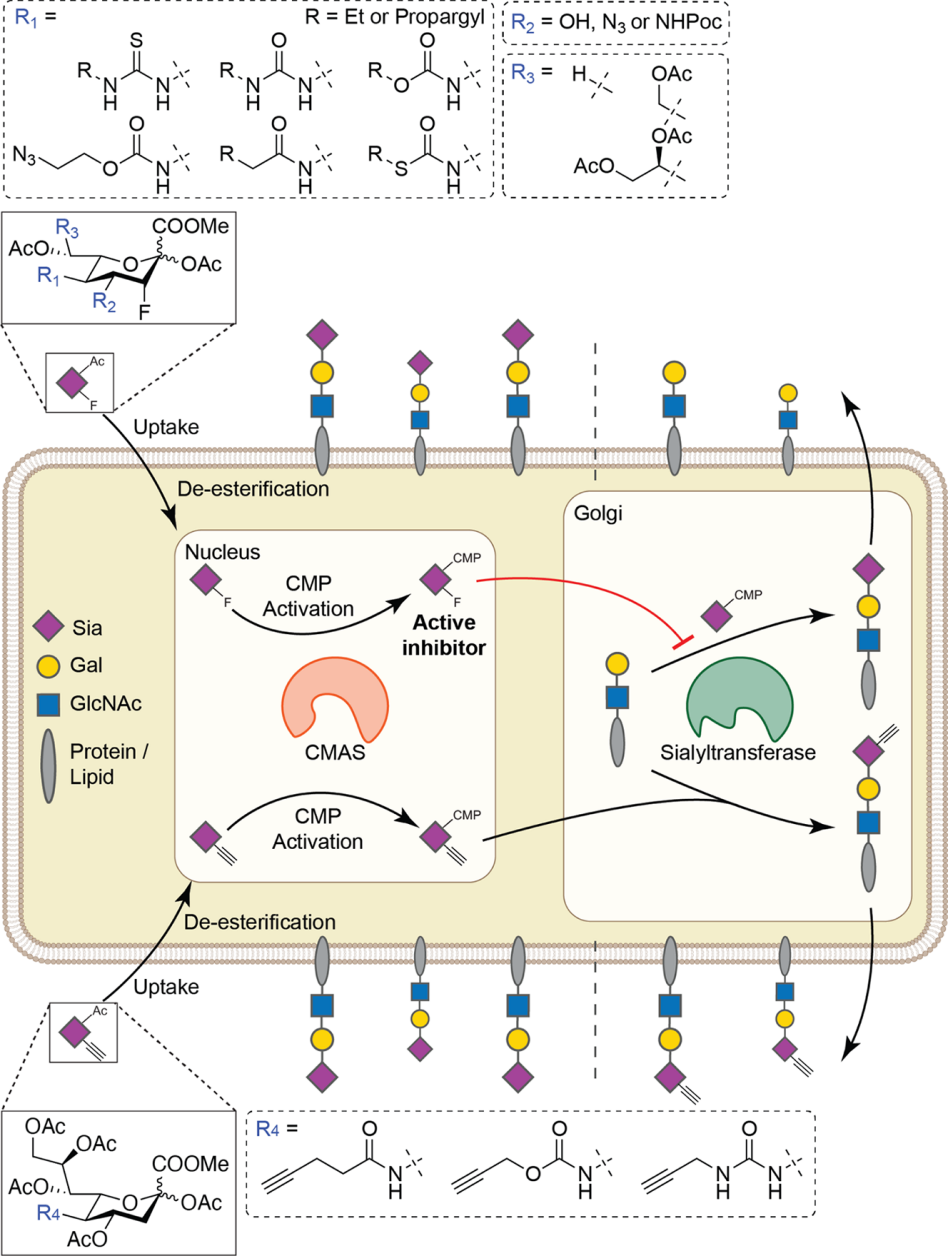
Structure and mode of action of metabolic sialylation inhibitors and metabolic labeling reagents. Sia = sialic acid, Gal = galactose, and GlcNAc = *N*-acetyl glucosamine.

Hence, to investigate the impact of further unnatural modifications, we herein report the synthesis and biological evaluation of a new panel of C-4-, C-5-, C-8-, and C-9-modified (3-fluoro-)sialic acid analogues. These derivatives can passively diffuse into the cell and are subsequently processed *via* the sialic acid metabolic pathway (Figure 1). We systematically substituted the C-5 *N*-acetamide of 3-fluoro-sialic acid for carbamate, *S*-thiocarbamate, urea, and thiourea groups. We found that inhibitory potency of the C-5 derivatives can be ordered as follows: carbamate > *S*-thiocarbamate > thiourea > urea > amide. Modifications at the glycerol side chain led to erosion of inhibitory potency but interestingly could be recovered by also introducing C-5 modifications. The results demonstrate a clear structure–activity relationship for the metabolism of unnatural analogues resulting in more potent inhibitors than the P-SiaFNAc parent compound. Indeed, this guideline could be translated to also improve the efficacy of sialic acid metabolic labeling reagents (Figure 1). C-5 propargyl carbamates outperformed the corresponding thiourea and amide analogues resulting in more efficient metabolic labeling.

## Results and Discussion

### Structure–Activity Relationship of Metabolic Sialic Acid Inhibitors

To enable the direct comparison of different C-5 derivatives, two sets of derivatives containing the same saturated [ethyl, **2**, **4**, **6**, **8**, **10**, **12** (azidoethyl)] or unsaturated (propargyl, **3**, **5**, **7**, **9**, **11**) chain length were prepared. Alkynes and azides are functional handles that can undergo orthogonal reactions in biological systems. We previously observed that the introduction of a heteroatom at C-5 (carbamate *vs* amide) led to increased metabolic processing by CMAS affording an increased intracellular concentration of the corresponding active CMP derivative.^20^ Hence, we investigated various heteroatom substitution patterns in the form of C-5 amide (a)-, carbamate (oc)-, urea (ur)-, thiourea (tur)-, and *S*-thiocarbamate (tc)-containing compounds the establish their effect on the inhibitory potency.

**Figure 2:**
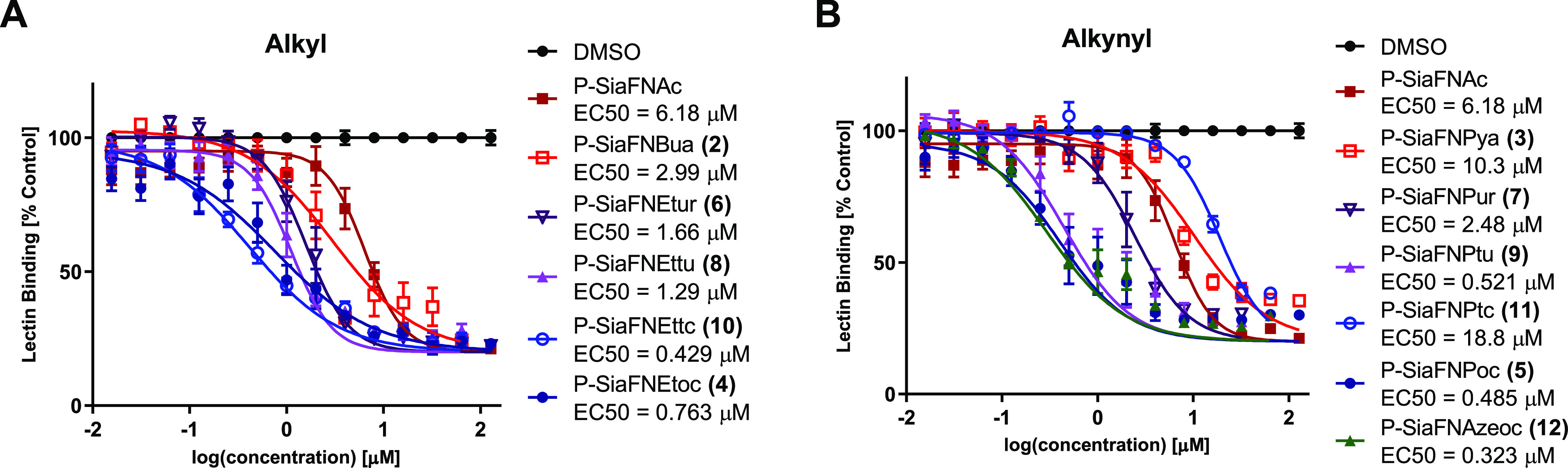
Dose-dependent inhibition of α2-3-linked sialic acid by (A) alkyl-substituted and (B) alkynyl-substituted fluorinated sialic acid analogues. THP-1 cells were cultured for 3 days with 0–256 μM fluorinated sialic acid analogues **2**–**12** and then stained with biotinylated MAL-II for α2-3-linked sialic acid, followed by streptavidin–PE. Fluorescence was measured using flow cytometry and normalized to a DMSO control. Values are plotted as mean ± standard error means.

**Scheme 1:**
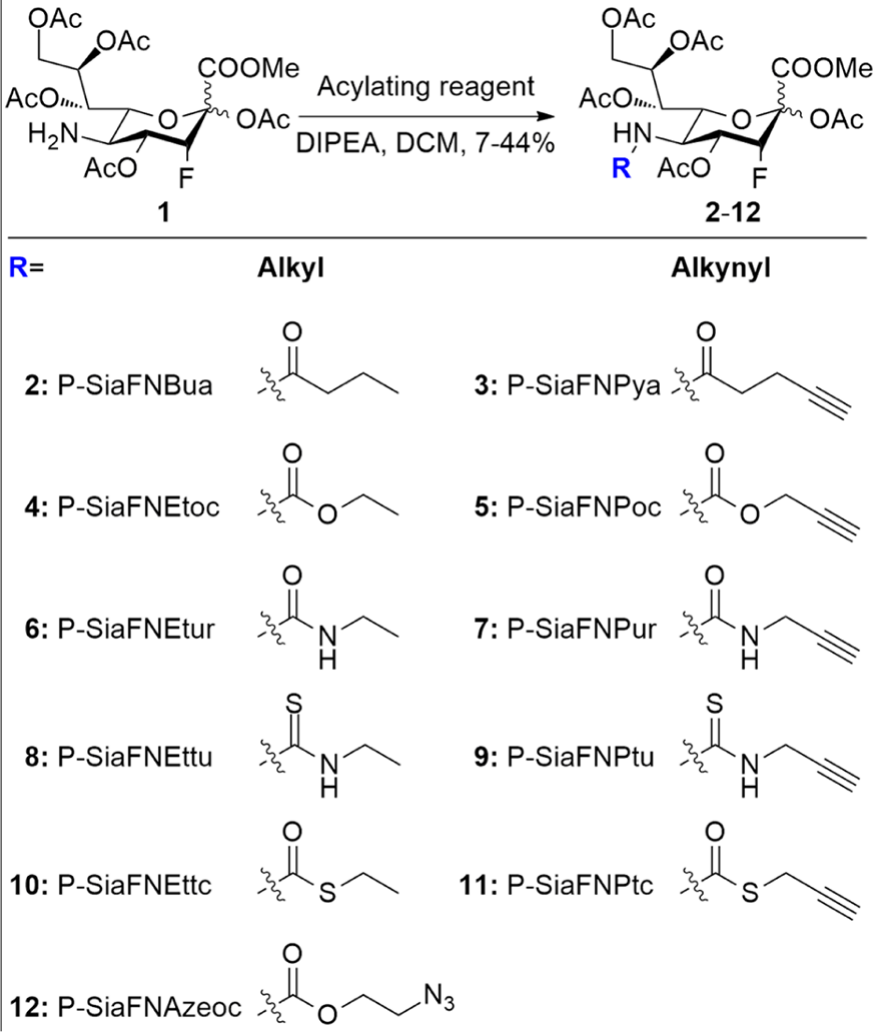
Synthesis of the SiaF Derivatives (**2**–**12**)

P-SiaFNAc derivatives **2**–**12** were prepared from the corresponding C-5 amine by acylation in low to moderate yields of 7–44% (Scheme 1). Reactions at the C-5 amine in presence of a C-3 fluorine do not proceed efficiently presumably due to the lower amine nucleophilicity. The potency of the inhibitors **2**–**12** was tested on human THP-1 monocytic leukemia cells (Figure 2). Inhibitor potency was assessed by incubating cells with a range of inhibitor concentrations for 3 days to allow for sialoglycan turnover. After 3 days, cellular sialylation was measured by staining for α2-3-linked sialic acids using the MAL-II lectin. The EC_50_ values were determined and defined as the concentration where a 50% decrease in lectin binding compared to the control (DMSO) was observed (Figure 2, Table 1).

**Table 1:** EC_50_ Values in μM for Inhibition of α2-3-Linked Sialic Acid (First Column) and α2-6-Linked Sialic Acid^a^

side group	P-SiaFNR	THP-1 (MAL-II)	THP-1 (SNA-I)	Jurkat (SNA-I)	GL261 (SNA-I)	B16F10 (SNA-I)	EL-4 (SNA-I)
	Ac	6.18	6.78	9.46	71.3	110	≥250
alkyl	Bua (**2**)	2.99	1.63	2.46	9.71	16.5	212
	Etoc (**4**)	0.763	**0.319**	**0.48**	5.71	11.5	77.7
	Etur (**6**)	1.66	1.76	3.32	11.2	29.4	46.1
	Ettu (**8**)	1.29	0.749	2.01	11.2	36.2	≥250
	Ettc (**10**)	**0.429**	0.780	1.29	7.81	11.9	89.1
alkynyl	Pya (**3**)	10.3	3.72	13.8	58.9	16.5	≥250
	Poc (**5**)	**0.485**	**0.199**	**1.00**	**4.40**	**7.10**	119
	Pur (**7**)	2.48	3.45	9.21	11.1	37.0	≥250
	Ptu (**9**)	0.521	1.92	5.08	16.3	197	**60.4**
	Ptc(**11**)^b^	18.8^b^	46.6^b^	N.D.	N.D.	N.D.	N.D.
other	Azeoc (**12**)	**0.323**	0.410	2.95	5.11	8.89	141

a Cells were cultured for 3 days with 0–256 μM fluorinated sialic acid analogues **2**–**12** and then stained with biotinylated SNA-I or MAL-II lectins, followed by streptavidin–PE. Fluorescence was measured using flow cytometry and normalized to a DMSO control.

b Ambiguous fit, due to toxicity at >32 μM.

All synthesized sialosides, with the exception of **3** and **11,** are more potent inhibitors than P-SiaFNAc. This is in line with earlier research that showed that a carbamate group enabled increased metabolic processing by CMAS. Inhibitors having an *S*-thiocarbamate, urea, or thiourea linkage also show an increased inhibitor potency compared to P-SiaFNAc. For both series of compounds (ethyl and propargyl), a trend emerges with the carbamate and thiocarbamate linkage being the most potent, followed by the thiourea, then the urea, and the amide derivative being the least potent inhibitor. Propargyl thiocarbamate **11** showed toxicity at relatively low concentrations. To validate this trend, the potency of the inhibitors was also measured in a broader set of cell lines: Jurkat acute T-lymphocyte leukemia cells and murine EL-4 (T-lymphocyte leukemia), GL261 (glioma), and B16F10 (melanoma) cancer cell lines. As staining with MAL-II can lead to unwanted binding to 3-*O*-sulfated galactose residues,^27^ we therefore switched to SNA-I, which binds α2-6-linked sialic acid. Sialylation was measured at different concentrations of the inhibitors, and EC_50_-values were calculated by fitting a dose–response curve and obtaining the concentration at which 50% of sialic acid remains on the cell surface.

**Scheme 2:**
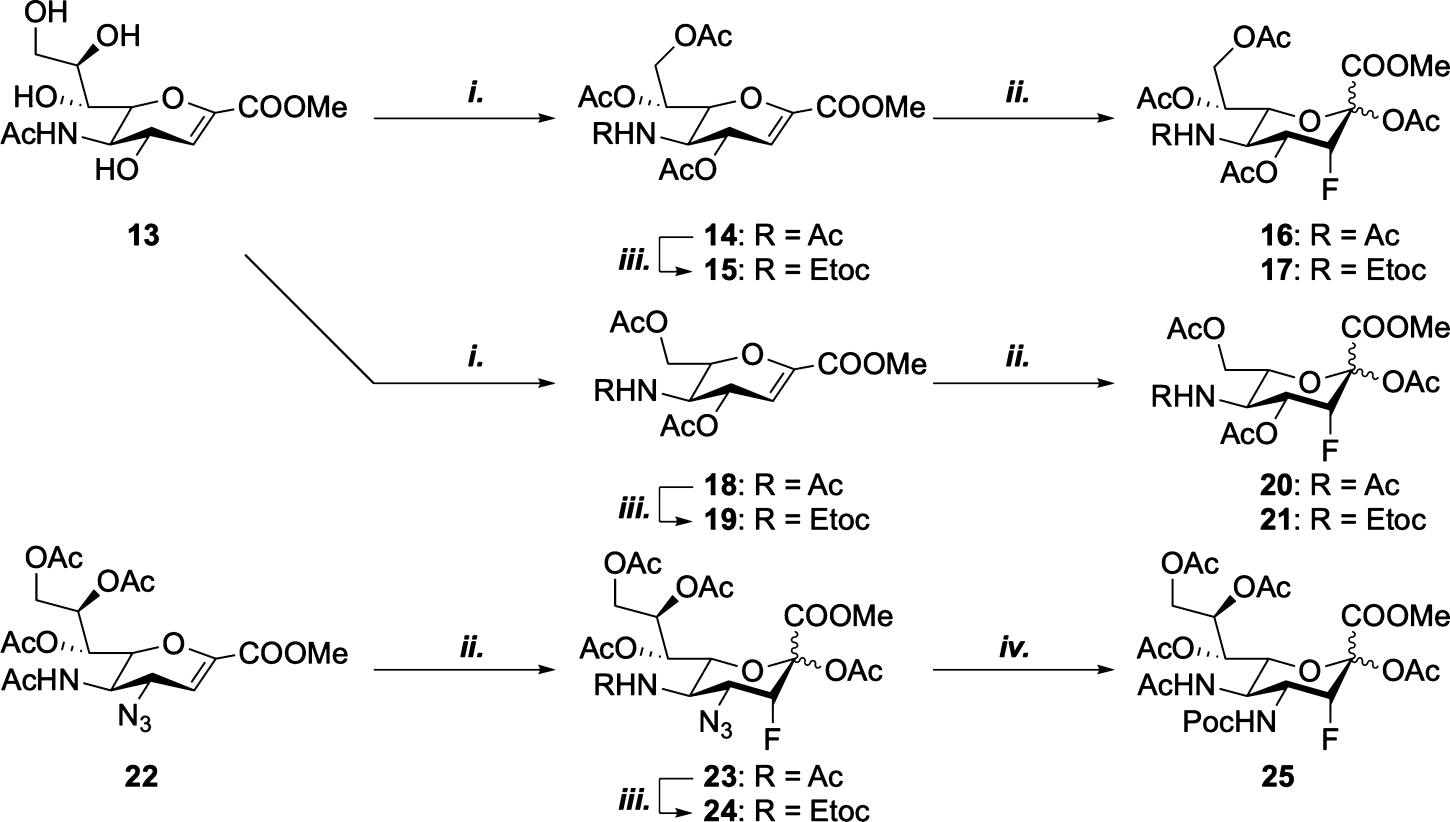
Synthesis of C-4-, C-7-, and C-8-Modified Sialic Acid Inhibitors; (i) (1) NaIO_4_, MeOH; (2) NaBH_4_, MeOH; (3) Ac_2_O, Py, 37% (**14**); 66% (**18**); (ii) (1) Selectfluor, DMF, H_2_O; (2) Ac_2_O, Py, 21% (**16**); 23% (**17**); 33% (**20**); 14% (**21**); 54% (**23**); (iii) (1) Tf_2_O, 2-FPy, DCM, then 1,2-Propanediol; (2) Ethyl Chloroformate, DIPEA, DCM, 68% (**15**); 39% (**19**); 28% (**24**); and (iv) PMe_3_, THF, then Propargyl Chloroformate, 24% (**25**)

For all cell lines, the new inhibitors also proved to be more potent than the parent compound P-SiaFNAc (Table 1). For several compounds, inhibition was found even in less-responsive murine cancer cell lines like EL-4. Although P-SiaFNAc shows low inhibitory potency (>50 μM) in B16F10 cells in vitro, it has been shown to reduce tumor growth in vivo.^14^ Across the five cell lines, the observed trend in potency versus the type of C-5 modification is in line with observations for THP-1 cells, with compounds **4**, **5**, **10,** and **12** being the most potent inhibitors. Next, we investigated the impact of modifications on the glycerol side chain of sialic acid (Scheme 2). Truncated octulosonic and heptulosonic acid inhibitors **16**, **17**, **20,** and **21** were synthesized, containing either a 7- or 8-carbon skeleton. Additionally, C-5 carbamate derivatives were prepared to enable the comparison of these truncated inhibitors to the most potent carbamate inhibitor (**4**). We were also interested in probing to what extent modifications at the C-4 and glycerol side chain contributed to the activity of the sialyltransferase inhibitors. Hence, we prepared derivatives **23** and **25** having an azide or propargyloxycarbonyl at the C-4 position and a dual-modified inhibitor **24**, which has a C-4 azide and C-5 carbamate.

Truncated derivatives **16**, **17**, **20,** and **21** were synthesized from sialic acid glycal **13**. Malaprade oxidation^28^ followed by sodium borohydride reduction and acetylation afforded **14** and **18**.^29^ Selective N-deacetylation followed by acylation with ethyl chloroformate afforded carbamates **15** and **19**. Electrophilic fluorination^30^ and subsequent acetylation of acetamides **14** and **18** and carbamates **15** and **19** afforded truncated inhibitors **16**, **17**, **20,** and **21**. Subsequent fluorination and acetylation of 4-N_3_-glucal **22**(31) afforded **23**. In a similar manner as described above, selective N-deacetylation of **23** followed by acylation gave the C-4- and C-5-modified inhibitor **24**. **25** was synthesized from **23** via a direct conversion of the azide to a propargyl carbamate.^32^

**Figure 3:**
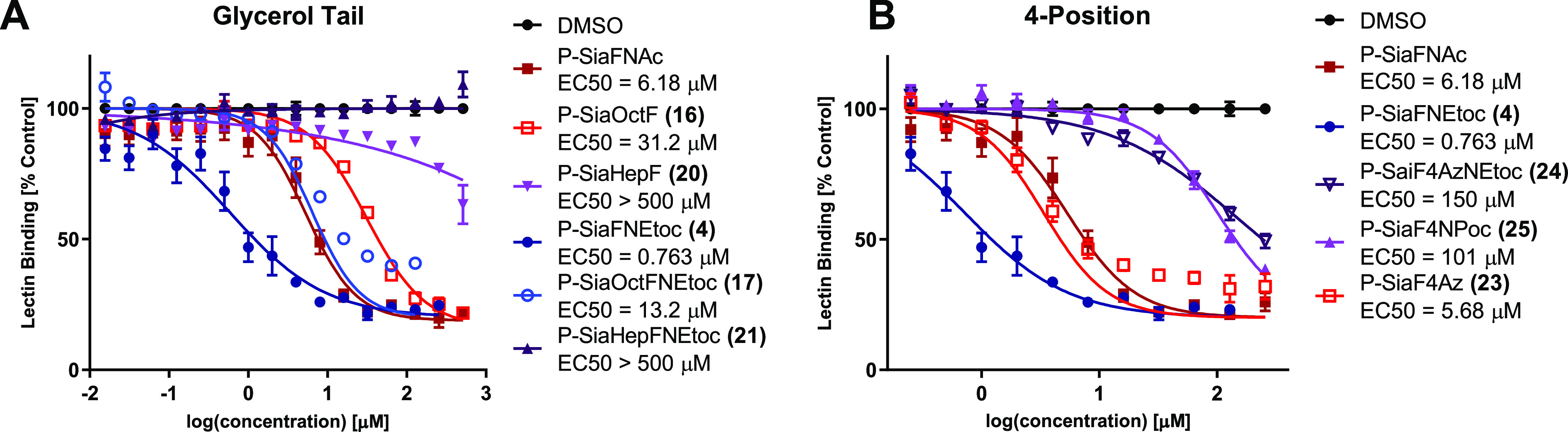
Dose-dependent inhibition of α2-3-linked sialic acid by (A) truncated and (B) C-4-substituted fluorinated sialic acid analogues. Cells were cultured for 3 days with 0–512 μM fluorinated sialic acid analogues **4**, **16**, **17**, **20**, **21,** and **23**–**25** and stained with biotinylated MAL-II for α2-3-linked sialic acid, followed by streptavidin–PE. Fluorescence was measured using flow cytometry and normalized to a DMSO control. Values are plotted as mean ± standard error means.

Using the aforementioned protocol, the inhibitory potency of **16**, **17**, **20**, **21,** and **23**–**25** was tested on THP-1 cells (Figure 3). Truncation of the glycerol tail leads to a length-dependent decrease in inhibition. Octulosonic acid **16** shows a loss of activity compared to P-SiaFNAc. Interestingly, substitution of the C-5 acetamide for a carbamate leads to a recovery of inhibitory potency. Both heptulosonic acids **20** and **21** barely show any activity. The introduction of a C-4 azide (**23**) leads to comparable activity compared to P-SiaFNAc. Swapping the azide for a propargyl carbamate (**25**) led to a loss of activity, indicating that only small modifications at this position are tolerated. Remarkably, a C-5 carbamate in combination with a C-4 azide (**24**) leads to a notable reduction of the inhibitory potency in contrast to earlier research showing that carbamate inhibitors were more potent.^20^

### Structure–Activity Relationship of Metabolic Sialic Acid Labeling Reagents

**Figure 4:**
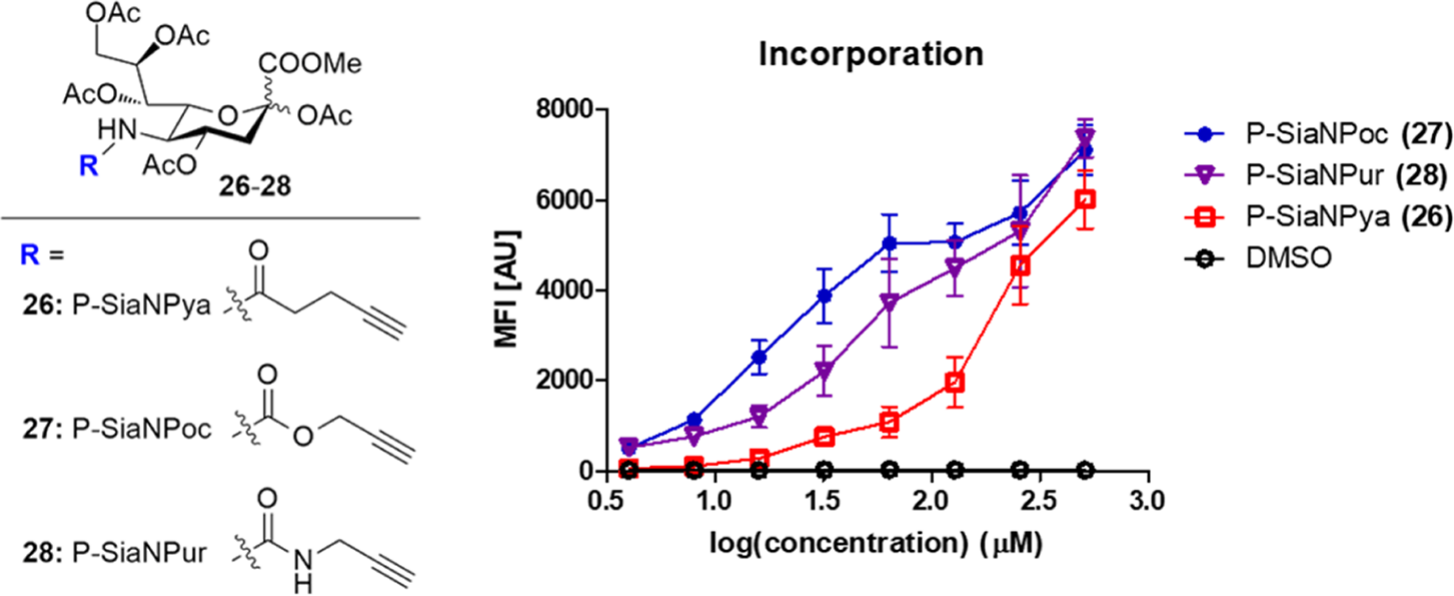
Incorporation of alkyne-containing sugars into cell surface glycans. THP-1 cells were cultured for 3 days with compounds (**26–28**) at concentrations of 4–512 μM and DMSO as the negative control (*n* = 3). Incorporation was visualized using CuAAC, and fluorescence was measured using flow cytometry. Mean fluorescence was plotted against the logarithmic concentration as mean ± standard error means.

From these results, it is clear that C-5 modification can be used to improve the efficiency of metabolic inhibitors. To investigate whether increased metabolic processing can also be employed to improve the metabolic labelling of sialic acids, three non-fluorinated alkyne-containing sialic acid derivatives (**26–28**) were prepared (see the Supporting Information). These derivatives carry the same alkyne reporter which is connected *via* a C-5 amide, urea, or carbamate group, analogous to compounds **3**, **5,** and **7** (Figure 4). THP-1 cells were incubated with 4–512 μM **26–28** for 3 days. Incorporation was visualized by conjugating the incorporated alkyne to azide-biotin, followed by incubating with streptavidin, conjugated to a phycoerythrin dye (PE). Fluorescence was then measured with flow cytometry. As shown in Figure 4, carbamate **27** shows the most efficient incorporation, followed by urea **28** and amide **26**. To minimize the impact of differences in alkyne reactivity in the biotin conjugation step, a large excess of the azide reagent was used to drive the reaction to completion. Furthermore, the use of high concentrations of the sialic acid derivative (**26–28**) leads to a similar plateau in the fluorescence signal, which would not be expected if differences in alkyne reactivity played a role. Moreover, the concentration-dependent incorporation of compounds **26–28** follows the same trend as the inhibitor analogues **3**, **5,** and **7**.

## Conclusions

We have prepared and tested a panel of C-4, C-5, C-7, and C-8 derivatives of (3-fluoro-)sialic acid. C-5 carbamate, urea, thiourea, and *S*-thiocarbamate derivatives are more potent inhibitors than the corresponding C-5 amides. Small modifications at the C-4 are tolerated, and adjustment of the glycerol side chain leads to a length-dependent decrease in inhibition. Incorporation of non-fluorinated analogues shows a similar trend with respect to the C-5 linkage as their fluorinated counterparts. These results could be considered for further inhibitor design and for sialosides that utilize the same metabolic pathway.

## Methods

### Cell Culture

THP-1 cells (TIB-202, ATCC) and Jurkat cells (TIB-152, ATCC) were cultured in RPMI-1640 medium containing 2 mM gutamine and 25 mM *N*-(2-hydroxyethyl)piperazine-*N*′-ethanesulfonic acid (Gibco, Life Technologies), supplemented with 10% v/v heat-inactivated fetal bovine serum (FBS) (Gibco, Life Technologies) and 1× antibiotic–antimycotic solution (100 units/m penicillin, 100 μg/mL streptomycin, and 0.25 μg/mL Fungizone) (Gibco, Life Technologies) and passaged every 3–4 day by seeding a fraction of 0.5–2 mln cells of the culture per 10 mL medium.

EL-4 cells (TIB-39, ATCC) were cultured in Dulbecco’s modified Eagle’s medium (DMEM) (Gibco, Life Technologies), supplemented with 10% v/v heat-inactivated FBS (Gibco, Life Technologies), 1× antibiotic–antimycotic solution (100 units/mL penicillin, 100 μg/mL streptomycin, and 0.25 μg/mL Fungizone) (Gibco, Life Technologies), and 2 mM glutamine (Gibco, Life Technologies) and passaged every 2–4 days by seeding a fraction of 0.5–1 mln cells of the culture per 10 mL medium.

B16F10 cells (CRL-6475, ATCC) and GL261 cells were cultured in DMEM (Gibco), supplemented with 10% v/v heat-inactivated FBS (Gibco, Life Technologies), 1× antibiotic–antimycotic solution (100 units/mL penicillin, 100 μg/mL streptomycin, and 0.25 μg/mL Fungizone) (Gibco, Life Technologies), and 2 mM glutamine (Gibco, Life Technologies) and passaged every 2–4 days at 70–80% confluency. For passaging a T75 flask (Corning), medium was removed, and the monolayer was washed with 10 mL of phosphate-buffered saline (PBS) pH 7.2 without CaCl_2_ and MgCl_2_ (Gibco, Life Technologies) before adding 3.5 mL of 0.05% trypsin/EDTA solution (Gibco, Life Technologies) in PBS. Cells were incubated at room temperature for 30–45 s after which the trypsin/EDTA solution was removed. Cells were incubated for an additional 2 min at 37 °C and 5.0% CO_2_ and resuspended in cell culture medium. Cells were then seeded at ∼40.000 cells/cm^2^ in new culture flasks.

### Lectin Staining of Cells

The protocol mentioned below was repeated until *n* ≥ 3 for each compound using cells at different passage numbers.

Cells were cultured in medium containing different concentrations of unnatural sugar derivatives on either a 48-well plate (adherent cell lines; 20,000–40,000 cells per well) (Corning) or a 96-well plate (suspension cell lines; 13,000–20,000 cells per well) (Thermo Scientific). DMSO, at same dilution as the unnatural sugar derivative stock solutions, was used as a positive control for lectin staining; P-SiaFNAc was used for all concentrations as the negative control. Cells were incubated for 3 days at 37 °C and 5% CO_2_ in a humidified incubator.

Cells were harvested and washed with 100 μL of 1× CF-blocking buffer (Vector Laboratories Inc.) containing 1 mM CaCl_2_ and 1 mM MgCl_2_. The cells were then resuspended in 50 μL of 0.5 μg/mL 0.5 ng/mL biotinylated lectin in 1× carbo-free blocking buffer and incubated at 4–8 °C for 45–60 min. Cells were washed with 3 × 100 μL of PBA (PBS-containing 1% v/v FBS and 0.1% w/w NaN_3_) and incubated with 40 μL of the 1 μg/mL streptavidin–phycoerythrin conjugate (Invitrogen, eBioscience) in PBA for 10–15 min at 4–8 °C. Cells were then washed again with 3 × 100 μL of PBA, resuspended in PBA, and fluorescence was measured with a flow cytometer (Beckman & Dickinson FACS-Calibur). Each replicate was obtained for each condition with >10,000 gated events. Data were processed using FlowJo (FlowJo LLC). The percentage of lectin binding was obtained by normalizing the MFI values to the MFI values of the respective DMSO control.

### Lectin Specificity Assay

The protocol mentioned below was repeated until *n* ≥ 3 for each compound using cells at different passage numbers.

Culture medium was prepared in 96-well plates (Thermo Scientific). For every unnatural sugar derivative, 11 wells containing 100 μM unnatural sugar derivatives and 11 wells containing 10 μM the unnatural sugar derivatives were prepared. DMSO, at same dilution as the tested probes’, was used as a positive control for lectin staining. THP-1 Cells were cultured in the plates (20,000 cells per well), and the cells were incubated for 3 days at 37 °C and 5% CO_2_.

Cells were harvested and washed with 100 μL of 1× CF-blocking buffer (Vector Laboratories Inc.) containing 1 mM CaCl_2_ and 1 mM MgCl_2_. The cells were then resuspended in 50 μL of 0.5 μg/mL either 0.5 ng/mL biotinylated AAL, AOL, SNA, MAL-II, WGA, LCA, PSA, PNA, PHA-L, or GSL-1 lectin in 1× carbo-free blocking buffer or with 50 μL of non-supplemented 1× carbo-free blocking buffer and incubated at 4–8 °C for 45–60 min. Cells were washed with 3 × 100 μL of PBA (PBS containing 1% v/v FBS and 0.1% w/w NaN_3_), incubated with 40 μL of the 1 μg/mL streptavidin–phycoerythrin conjugate (Invitrogen, eBioscience) in PBA for 10–15 min at 4–8 °C. Cells were then washed again with 3 × 100 μL of PBA, resuspended in PBA, and fluorescence was measured with a flow cytometer (Beckman & Dickinson FACS-Calibur). Each replicate was obtained for each condition with >10,000 gated events. Data were processed using FlowJo (FlowJo LLC). The percentage of lectin binding was obtained by normalizing the MFI values to the MFI values of the respective DMSO control.

### CuAAC Staining of the Cell Membrane

The protocol mentioned below was repeated until *n* ≥ 3 for each compound using cells at different passage numbers.

THP-1 cells were cultured in medium containing 4–512 μM unnatural sugar derivatives on 96-well plates (20,000 cells per well) (Thermo Scientific). DMSO, at the same dilution as the unnatural sugar derivative stock solutions’, was used as the negative control. Cells were incubated for 3 days at 37 °C and 5% CO_2_ in a humidified incubator.

Cells were harvested and washed with 100 μL of 2× PBS. The cells were then resuspended in 95 μL of reaction buffer [250 μM CuSO_4_, 200 μM l-histidine, 100 μM of azide-PEG3-biotin conjugate (click chemistry tools) in PBS], and 5 μL of a freshly made solution of sodium ascorbate (10 mM in PBS, final concentration of 500 μM) was added, after which cells were incubated at 37 °C for 20 min. Cells were washed with 3 × 100 μL of PBS containing 1% w/v BSA without NaN_3_ and incubated with 40 μL of the 1 μg/mL streptavidin–phycoerythrin conjugate (BD, Pharmingen) in PBA (PBS containing 1% w/v BSA with 0.2% w/v NaN_3_) for 20 min at 4–8 °C. Cells were then washed again with 3 × 100 μL of PBA, resuspended in PBA, and fluorescence was measured using a flow cytometer (Beckman Coulter FACS-CyAn ADP). Each replicate was obtained for each condition with >10,000 gated events. Data were processed using FlowJo (FlowJo LLC) and Graphpad Prism v5.0.

## Supporting Information Available

Supplemental table, synthetic procedures, and characterization of new compounds (PDF)
