# Supplementary material for: Structure–Activity Relationship of Metabolic Sialic Acid Inhibitors and Labeling Reagents
Source: ACS Chem Biol. 2022 Feb 18;17(3):590–7. doi: 10.1021/acschembio.1c00868 (PMC8938927; doi:10.1021/acschembio.1c00868)
Supplement: Supplementary file 1 — cb1c00868_si_001.pdf [file cb1c00868_si_001.pdf]

Supporting information

The Structure – Activity Relationship of Metabolic Sialic Acid Inhibitors and Labeling Reagents

Sam J. Moons[a#], Emiel Rossing[a#], Mathilde A.C.H. Janssen[a], Torben Heise[a], Christian Büll[b], Gosse J. Adema[c] and Thomas J. Boltje[a*]

a. Cluster of Molecular Chemistry, Institue for Molecules and Materials, Radboud University Nijmegen, Nijmegen 6525 AJ,The Netherlands
b. Department of Biomolecular Chemistry, Institute for Molecules and Materials, Radboud University Nijmegen, Nijmegen 6525 GA, The Netherlands
c. Radiotherapy & OncoImmunology Laboratory, Department of Radiation Oncology, Radboud University Medical Center, Nijmegen 6525 GA, The Netherlands
*e-mail: thomas.boltje@ru.nl

[#]These authors contributed equally

Supplementary table

Table (S-1): EC_{50} values in µM for inhibition of α2,3-linked sialic acid (first column) and α2,6-linked sialic acid. Cells were cultured for three days with 0-512 µM fluorinated sialic acid analogs and then stained with biotinylated SNA- or MAL-II lectins, followed by streptavidin-PE. Fluorescence was measured using flow cytometry and normalized to a DMSO control.

site	compound	THP-1 (MAL-II)	THP-1 (SNA)
	P-SiaFNAc	6.18	6.78
5-position	P-SiaFEtoc **(4)**	0.763	0.319
4-position	P-SiaF4Az **(23)**	5.68	34.2
	P-SiaF4NPoc **(25)**	101	342
	P-SiaF4AzNEtoc **(24)**	150	>500
Glycerol tail	P-SiaHepF **(16)**	>500	>500
	P-SiaOctF **(20)**	31.2	28.6
	P-SiaHepFNEtoc **(17)**	>500	>500
	P-SiaOctFNEtoc **(21)**	13.2	4.33

**EC50 (µM)**
>50
>10-50
>5-10
1-5
<1

Reagents

Biotinylated SNA, MAL-II, AAL, WGA, LCA, PSA, PNA, GSL-I and PHA-L lectins and 10x Carbo-free Blocking Buffer were purchased from Vector laboratories Inc.. Biotinylated AOL lectin was purchased from TCI Europe. 0.2 mg/ml Streptavidin-phycoerythrin conjugate was purchased from Fischer Scientific (Invitrogen, eBioscience). Unnatural sugar derivatives were stored at -20°C at a stock concentration of 100 mM/DMSO.

General methods for synthesis

Chemicals were purchased at Sigma Aldrich, TCI Europe, Fisher Scientific, Fluorochem or Carbosynth and used without further purification. NMR spectra were recorded on a Bruker Avance III 400 MHz or a Bruker 500 MHz spectrometer and the compounds were assigned using 1H NMR,13C NMR, COSY, HSQCED and HMBC spectra. Chemical shifts were reported in parts per million (ppm.) relative to reference ($CDCl_3$: ^{1}H: 7.26 ppm. and ^{13}C 77.16 ppm. NMR data are presented in the following way: chemical shift, multiplicity (s = singlet, bs = broad singlet, d = doublet, t = triplet, dd = doublet of doublets, h = heptet, m = multiplet and/or multiple resonances) and coupling constants J in Hz. Reactions were monitored using TLC F_{254} (Merck KGaA) using UV absorption detection (254 nm) and by spraying them with potassium permanganate or 10% conc. H_2SO_4 in MeOH or cerium ammonium molybdate stain (Hannesian's stain) followed by charring at 300°C. Mass spectra were recorded on a JEOL AccuTOF CS JMS-T100CS (ESI) mass spectrometer. Purification by flash column chromatography was executed using automatic flash column chromatography on a Biotage Isolera Spektra One using Silicycle cartridges (Biotage, 30-100 µm, 60 Å) 4-120 g. Reactions under protective atmosphere were performed under positive Ar/N_2 flow in flame-dried flasks. Reactions were performed at room temperature unless stated otherwise.

Compounds **3 – 5**[1] and **27**[2] were prepared as described previously.

General method A for the synthesis of sialic acid derivatives 2, 6 – 10, 12

Sialic acid amine **1** was dissolved in DCM (0.1M) and cooled to 0°C, followed by addition of DIPEA (3 eq.) and the corresponding acylating reagent (3 eq.). After stirring at rt for 16h, the mixture was concentrated *in vacuo*. The residue was dissolved in EtOAc and washed with 0.1M HCl and brine. The aqueous layer was washed with DCM. The combined organic layers were dried over $MgSO_4$, filtered and concentrated *in vacuo*. The crude product was purified using flash column chromatography (0% to 60% EtOAc in heptane) to obtain the product.

Specific methods

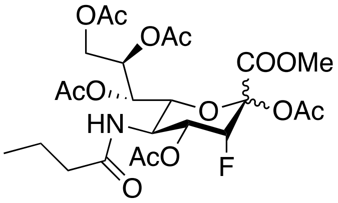

Methyl (5-butylamido-4,7,8,9-tetra-O-acetyl-3-dehydro-3,5-dideoxy-3-fluoro-5-β-D-glycero-D-galacto)onate (2)

Using general method A with butyric anhydride afforded **2** (9.5 mg, 26%)

^{1}H NMR (500 MHz, $CDCl_3$) δ 5.63 (ddd, *J* = 27.9, 11.1, 2.5 Hz, 1H), 5.43 (d, *J* = 8.9 Hz, 1H), 5.32 (dd, *J* = 5.5, 1.9 Hz, 1H), 5.14 (td, *J* = 5.9, 2.5 Hz, 1H), 4.95 (dd, *J* = 49.1, 2.6 Hz, 1H), 4.54 (dd, *J* = 12.5, 2.5 Hz, 1H), 4.36 – 4.29 (m, 1H), 4.20 (dd, *J* = 12.5, 6.2 Hz, 1H), 4.14 – 4.05 (m, 1H), 3.84 (s, 3H), 2.18 (s, 3H), 2.17 (s, 3H), 2.10 (s, 3H), 2.04 (s, 3H), 2.03 (s, 3H), 1.62 (dtt, *J* = 18.8, 7.2, 3.6 Hz, 5H), 0.93 (t, *J* = 7.4 Hz, 3H).
^{13}C NMR (126 MHz, $CDCl_3$) δ 173.24, 170.70, 170.52, 170.36, 170.19, 167.13, 165.10, 95.27, 95.04, 87.76, 86.29, 71.56, 71.10, 68.16, 68.02, 67.97, 62.05, 53.48, 45.84, 38.71, 20.85, 20.82, 20.72, 20.68, 20.56, 18.87, 13.58**.** **^{19}F NMR** (470 MHz, $CDCl_3$) δ -209.06 – -209.34 (m).
HRMS (ESI-TOF) (*m/z*): $[M+H]^+$ calcd. for $C_{23}H_{34}FNO_{14}S$: 580.2042; found, 580.2067**.**
TLC (EtOAc) R_f = 0.7

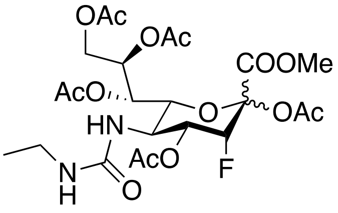

Methyl (5-ethylureido-4,7,8,9-tetra-O-acetyl-3-dehydro-3,5-dideoxy-3-fluoro-5-β-D-glycero-D-galacto)onate (6)

Using general method A with ethyl isocyanate afforded **6** (14 mg, 35%)

^{1}H NMR (500 MHz, $CDCl_3$) δ 5.54 (ddd, *J* = 28.4, 11.0, 2.4 Hz, 1H), 5.42 (dd, *J* = 5.0, 2.0 Hz, 1H), 5.14 (ddd, *J* = 7.3, 5.0, 2.5 Hz, 1H), 4.94 (dd, *J* = 49.2, 2.5 Hz, 1H), 4.63 – 4.55 (m, 2H), 4.51 (d, *J* = 9.1 Hz, 1H), 4.28 – 4.16 (m, 2H), 4.03 (d, *J* = 10.2 Hz, 1H), 3.84 (s, 3H), 3.13 (dq, *J* = 12.8, 7.2 Hz, 2H), 2.18 (s, 6H), 2.11 (s, 3H), 2.05 (s, 3H), 2.03 (s, 3H), 1.10 (t, *J* = 7.2 Hz, 3H). **^{13}C NMR** (126 MHz, $CDCl_3$) δ 170.77, 170.67, 170.61, 170.46, 167.30, 165.27, 156.82, 95.33, 95.10, 88.00, 86.53, 72.51, 71.51, 69.06, 68.92, 68.25, 62.25, 53.45, 46.45, 35.39, 20.89, 20.83, 20.75, 20.71, 20.56, 15.31. **^{19}F NMR** (470 MHz, $CDCl_3$) δ -208.73 (dd, *J* = 48.9, 28.4 Hz). **HRMS** (ESI-TOF) (*m/z*): $[M+Na]^+$ calcd. for $C_{23}H_{33}FN_2O_{14}Na$: 603.1814; found, 603.1806. **TLC** (EtOAc/Heptane, 5/5, v/v) R_f = 0.2

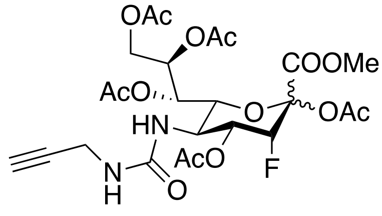

Methyl (5-propargylureido-4,7,8,9-tetra-O-acetyl-3-dehydro-3,5-dideoxy-3-fluoro-5-β-D-glycero-D-galacto)onate (7)

Using general method A with 4-nitrophenyl N-propargyl carbamate[3] afforded **7** (12 mg, 27%)

^{1}H NMR (500 MHz, $CDCl_3$) δ 5.54 (ddd, *J* = 27.9, 11.0, 2.5 Hz, 1H), 5.40 (dd, *J* = 5.1, 2.0 Hz, 1H), 5.15 (ddd, *J* = 6.7, 5.0, 2.5 Hz, 1H), 4.94 (dd, *J* = 49.2, 2.5 Hz, 1H), 4.65 (dd, *J* = 6.2, 4.8 Hz, 1H), 4.58 (dd, *J* = 12.5, 2.5 Hz, 1H), 4.44 (d, *J* = 9.2 Hz, 1H), 4.24 – 4.18 (m, 2H), 4.06 – 4.02 (m, 1H), 4.00 (dd, *J* = 6.2, 2.5 Hz, 1H), 3.84 (s, 3H), 2.25 – 2.20 (m, 2H), 2.18 (s, 3H), 2.18 (s, 3H), 2.13 (s, 3H), 2.05 (s, 3H), 2.04 (s, 4H).
^{13}C NMR (126 MHz, $CDCl_3$) δ 170.82, 170.73, 170.60, 170.40, 167.23, 165.18, 156.09, 95.30, 95.07, 87.95, 86.48, 72.36, 71.44, 71.36, 68.71, 68.64, 68.22, 62.21, 53.47, 46.71, 30.23, 20.91, 20.78, 20.76, 20.60. **HRMS** (ESI-TOF) (*m/z*): $[M+Na]^+$ calcd. for $C_{24}H_{3}$`$FN_2O_{14}Na$: 613.1657; found, 613.1680**.** **TLC** (EtOAc/Heptane, 5/5, v/v) R_f = 0.2

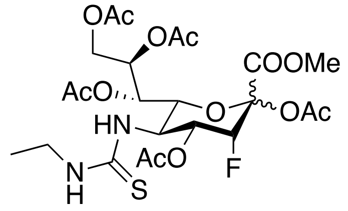

Methyl (5-ethylthioureido-4,7,8,9-tetra-O-acetyl-3-dehydro-3,5-dideoxy-3-fluoro-5-β-D-glycero-D-galacto)onate (8)

Using general method A with ethyl isothiocyanate afforded **8** (6.0 mg, 20%)

^{1}H NMR (500 MHz, $CDCl_3$) δ 6.17 – 6.10 (m, 1H), 6.04 – 5.98 (m, 0H), 5.49 – 5.24 (m, 2H), 5.24 – 5.12 (m, 1H), 4.97 (dd, *J* = 49.1, 2.4 Hz, 1H), 4.57 (d, *J* = 12.6 Hz, 1H), 4.23 (dd, *J* = 12.4, 6.6 Hz, 1H), 4.19 – 4.10 (m, 0H), 3.87 (s, 2H), 3.52 – 3.36 (m, 3H), 2.22 (s, 2H), 2.20 (s, 3H), 2.15 (s, 2H), 2.09 (s, 3H), 2.05 (s, 3H), 1.25 (t, *J* = 7.2 Hz, 3H). **^{13}C NMR** (126 MHz, $CDCl_3$) δ 170.42, 167.00, 164.96, 87.95, 86.47, 68.44, 62.21, 53.33, 50.57, 21.14, 20.80, 20.67, 20.60, 20.48, 13.89. **HRMS** (ESI-TOF) (*m/z*): $[M+Na]^+$ calcd. for $C_{23}H_{33}FN_2O_{13}SNa$: 619.1585; found, 619.1569**. TLC** (EtOAc/Heptane, 7/3, v/v) R_f = 0.2

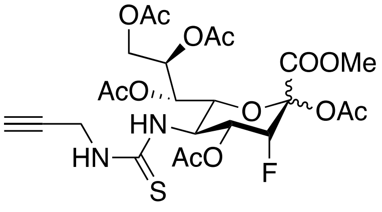

Methyl (5-propargylthioureido-4,7,8,9-tetra-O-acetyl-3-dehydro-3,5-dideoxy-3-fluoro-5-β-D-glycero-D-galacto)onate (9)

Using general method A with propargyl isothiocyanate afforded **9** (4.4 mg, 14%)

^{1}H NMR (500 MHz, $CDCl_3$) δ 6.58 (s, 1H), 5.87 (s, 1H), 5.55 – 5.32 (m, 2H), 5.23 – 5.06 (m, 2H), 5.02 – 4.85 (m, 1H), 4.56 (dd, *J* = 12.4, 2.7 Hz, 1H), 4.39 (m, 1H), 4.27 – 4.18 (m, 3H), 3.86 (s, 3H), 2.32 (t, *J* = 2.6 Hz, 1H), 2.21 (s, 3H), 2.18 (s, 3H), 2.13 (s, 3H), 2.08 (s, 3H), 2.04 (s, 3H).**^{13}C NMR** (126 MHz, DMSO) δ 170.29, 169.90, 169.57, 169.55, 169.46, 167.55, 167.52, 164.94, 125.32, 95.28, 95.05, 88.25, 86.80, 73.63, 72.55, 70.23, 68.11, 62.07, 62.01, 53.46, 34.79, 21.05, 20.95, 20.86, 20.84, 20.62. **HRMS** (ESI-TOF) (*m/z*): $[M+Na]^+$ calcd. for $C_{24}H_{31}FN_2O_{13}SNa$: 629.1429; found, 629.1433**. TLC** (EtOAc/Heptane, 7/3, v/v) R_f = 0.2

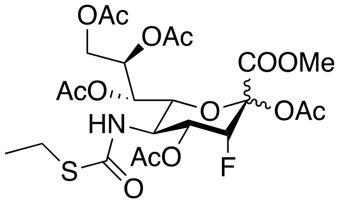

Methyl (5-S-ethylthiocarbamado-4,7,8,9-tetra-O-acetyl-3-dehydro-3,5-dideoxy-3-fluoro-5-β-D-glycero-D-galacto)onate (10)

Using general method A with S-ethylchlorothioformate afforded **10** (10mg, 23%) .

^{1}H NMR (500 MHz, $CDCl_3$) δ 5.54 (dd, *J* = 27.8, 10.6 Hz, 1H), 5.41 (d, *J* = 8.9 Hz, 1H), 5.34 (dd, *J* = 5.5, 1.9 Hz, 1H), 5.17 (td, *J* = 6.0, 2.5 Hz, 1H), 4.94 (dd, *J* = 49.1, 2.5 Hz, 1H), 4.53 (dd, *J* = 12.4, 2.6 Hz, 1H), 4.26 – 4.04 (m, 3H), 3.84 (s, 3H), 2.86 (q, *J* = 7.3 Hz, 2H), 2.18 (s, 3H), 2.17 (s, 3H), 2.12 (s, 3H), 2.05 (s, 3H), 2.04 (s, 3H), 1.27 (t, *J* = 7.3 Hz, 3H). **^{13}C NMR** (126 MHz, $CDCl_3$) δ 170.60, 170.33, 170.19, 167.11, 165.03, 95.22, 94.99, 87.86, 86.38, 71.77, 70.95, 68.11, 62.09, 53.49, 47.24, 24.58, 20.84, 20.75, 20.64, 20.55, 15.53. **^{19}F NMR** (470 MHz, $CDCl_3$) δ -209.00 (dd, *J* = 49.0, 28.2 Hz). **HRMS** (ESI-TOF) (*m/z*): $[M+Na]^+$ calcd. for $C_{23}H_{33}FNO_{14}SNa$: 620.1425; found, 620.1415. **TLC** (EtOAc/Heptane, 7/3, v/v) R_f = 0.5

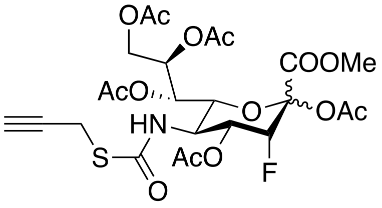

Methyl (5-S-propargylthiocarbamado-4,7,8,9-tetra-O-acetyl-3-dehydro-3,5-dideoxy-3-fluoro-5-β-D-glycero-D-galacto)onate (11)

Sialic acid amine **1** was dissolved in dry DCM (0.1M). DIPEA (5 eq.) and triphosgene (1 eq.) were added. After MS showed consumption of the free amine, a solution of propargyl thiol (2 eq.) in MeOH was added. The mixture was stirred overnight, concentrated and purified using flash column chromatography (0-60% EtOAc in heptane) affording the product (7.5 mg, 14%)

^{1}H NMR (500 MHz, $CDCl_3$) δ 5.63 – 5.49 (m, 2H), 5.33 (dd, *J* = 5.6, 1.9 Hz, 1H), 5.17 (td, *J* = 5.9, 2.5 Hz, 1H), 4.95 (dd, *J* = 49.1, 2.5 Hz, 1H), 4.53 (dd, *J* = 12.5, 2.6 Hz, 1H), 4.27 – 4.16 (m, 2H), 4.16 – 4.05 (m, 1H), 3.84 (s, 3H), 3.73 (dd, *J* = 16.6, 2.7 Hz, 1H), 3.55 (dd, *J* = 16.6, 2.7 Hz, 1H), 2.20 (t, *J* = 2.6 Hz, 1H), 2.18 (s, 3H), 2.17 (s, 3H), 2.14 (s, 3H), 2.06 (s, 3H), 2.05 (s, 3H). **^{13}C NMR** (126 MHz, $CDCl_3$) δ 170.67, 170.58, 170.41, 170.26, 167.13, 166.27, 164.98, 95.17, 94.94, 87.80, 86.32, 79.10, 71.50, 71.21, 70.85, 68.11, 68.03, 62.05, 53.53, 47.70, 20.86, 20.76, 20.67, 20.54, 18.55. **^{19}F NMR** (470 MHz, $CDCl_3$) δ -209.07 (dd, *J* = 49.2, 27.6 Hz). **HRMS** (ESI-TOF) (*m/z*): $[M+Na]^+$ calcd. for $C_{24}H_{30}FNO_{14}SNa$: 630.1269; found, 630.1255. **TLC** (EtOAc/Heptane, 7/3, v/v) R_f = 0.5

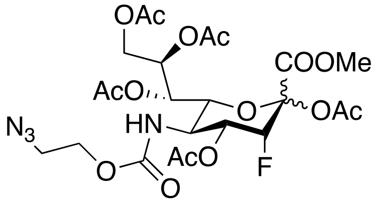

Methyl (5-azidoethylcarbamado-4,7,8,9-tetra-O-acetyl-3-dehydro-3,5-dideoxy-3-fluoro-5-β-D-glycero-D-galacto)onate(12)

Using general method A with 2-azidoethyl succinimidyl carbonate afforded **12** (19 mg, 43%)

^{1}H NMR (500 MHz, $CDCl_3$) δ 5.53 (dd, *J* = 28.0, 11.3 Hz, 1H), 5.40 (dt, *J* = 6.2, 3.1 Hz, 1H), 5.21 – 5.13 (m, 1H), 4.95 (dd, *J* = 49.1, 2.5 Hz, 1H), 4.86 (d, *J* = 9.3 Hz, 1H), 4.54 (dd, *J* = 12.5, 2.5 Hz, 1H), 4.34 – 4.03 (m, 6H), 3.97 – 3.85 (m, 1H), 3.84 (s, 3H), 3.58 – 3.45 (m, 2H), 3.40 – 3.30 (m, 1H), 2.18 (s, 3H), 2.17 (s, 3H), 2.13 (s, 3H), 2.04 (s, 3H), 2.04 (s, 3H). **^{13}C NMR** (126 MHz, $CDCl_3$) δ 170.61, 170.09, 167.10, 165.02, 154.90, 95.18, 94.95, 87.77, 86.30, 72.00, 71.89, 70.99, 68.03, 67.99, 64.19, 61.98, 53.50, 50.00, 47.08, 20.83, 20.74, 20.64, 20.55. **^{19}F NMR** (470 MHz, $CDCl_3$) δ -209.08 (dd, *J* = 49.0, 27.8 Hz). **HRMS** (ESI-TOF) (*m/z*): $[M+Na]^+$ calcd. for $C_{23}H_{31}FN_4O_{15}Na$: 645.1668; found, 645.1654 **TLC** (EtOAc) R_f = 0.4

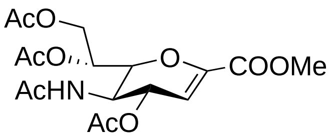

Methyl (5-acetemido-4,7,8-tri-O-acetyl-3-dehyrdo-2,3,5-trideoxy-5-D-galacto)onate (14)

To a solution of sialic acid glucal (**13**) (794 mg, 2.60 mmol) in anhydrous MeOH (0.1M), $NaIO_4$ (1.0 eq.) was added. The reaction mixture was stirred at rt overnight and was filtered through a celite pad. The pad was washed with MeOH and the filtrate was evaporated under reduced pressure. The residue was dissolved in anhydrous MeOH (0.1M), cooled to 0°C and $NaBH_4$ (2.4 eq.) was added. The reaction mixture was stirred for 1 h after which it was filtered through a celite pad. The pad was washed with MeOH and filtrate was evaporated under reduced pressure. The product was purified using flash column chromatography (0 to 20% MeOH in DCM) giving the product, which was dissolved in pyridine (44 eq.), after which Ac_2O (22 eq.) was added. The mixture

was stirred overnight at rt. The solvent was concentrated, the residue was dissolved in EtOAc and washed with 1M HCl (3x). The aqueous layer was washed with DCM, and the organic layers were combined, dried with $MgSO_4$ and concentrated. The crude product was purified using flash column chromatography (0 to 100% EtOAc in heptane), yielding the product (38% over three steps)

^{1}H NMR (400 MHz, $CDCl_3$) δ 6.03 (d, *J* = 3.3 Hz, 1H, H-3), 5.48 – 5.41 (m, 2H, H-4; H-7), 4.46 (dd, *J* = 12.0, 4.2 Hz, 1H, H-8), 4.42 – 4.38 (m, 1H, H-5), 4.34 (ddd, *J* = 7.9, 4.4, 0.8 Hz, 1H, H-6), 4.25 (dd, *J* = 11.9, 6.7 Hz, 1H, H-8), 3.81 (s, 3H, OCH_3), 2.12 (s, 3H, CH_3, Ac), 2.08 (s, 3H, CH_3, Ac), 2.07 (s, 3H, CH_3, Ac), 1.94 (s, 3H, CH_3, Ac). **^{13}C NMR** (101 MHz, $CDCl_3$) δ 170.71 (CO, Ac), 170.50 (CO, Ac), 170.46 (CO, Ac), 169.93 (CO, Ac), 161.71 (C-1), 145.07 (C-2), 107.61 (C-3), 76.55 (C-6), 67.39 (C-4/C-7), 66.90 (C-4/C-7), 62.67 (C-8), 52.61 (OCH_3), 46.52 (C-5), 23.18 (CH_3, Ac), 20.91 (CH_3, Ac), 20.87 (CH_3, Ac), 20.74 (CH_3, Ac). **HRMS** (ESI-TOF) (*m/z*): $[M+Na]^+$ calcd. For $C_{17}H_{23}NO_{10}Na$, 424.12196 found 424.12213. **TLC** (EtOAc) R_f = 0.4

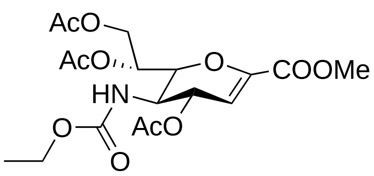

Methyl (5-ethylcarbamado-4,7,8-tri-O-acetyl-3-dehyrdo-2,3,5-trideoxy-5-D-galacto)onate (15)

14 (64.2 mg, 160 μmol) was dissolved in anhydrous DCM (0.1M) and cooled to 0°C under inert atmosphere. 2-fluorpyridine (4 eq.) was added, followed by Tf_2O (1.5 eq.). After 1 h, the mixture was warmed to rt and 1,2-propanediol (4 eq.) was added. After 1h, the mixture was cooled to 0°C, followed by addition of DIPEA (3 eq.) and ethyl chloroformate (3 eq.). The reaction mixture was stirred overnight. The solvent was evaporated under reduced pressure and the crude product was purified using flash column chromatography (0 to 60% EtOAc in Heptane) giving the desired product (68%)

^{1}H NMR (400 MHz, $CDCl_3$) δ 6.01 (d, *J* = 3.2 Hz, 1H), 5.58 – 5.34 (m, 2H), 5.05 (d, *J* = 9.5 Hz, 1H), 4.46 (dd, *J* = 11.8, 4.4 Hz, 1H), 4.37 – 4.22 (m, 2H), 4.19 – 4.00 (m, 3H), 3.80 (s, 3H), 2.12 (s, 3H), 2.09 (s, 3H), 2.06 (s, 3H), 1.24 – 1.17 (m, 3H). **^{13}C NMR** (101 MHz, $CDCl_3$) δ 170.76, 170.59, 170.43, 170.36, 161.75, 155.79, 107.82, 76.72, 68.09, 67.05, 62.63, 61.50, 52.55, 48.16, 20.89, 20.84, 20.71, 14.44. **HRMS** (ESI-TOF) (*m/z*): $[M+Na]^+$ calcd. For $C_{18}H_{25}NO_{11}Na$, 454.1325; Found 454.1321 **TLC** (EtOAc) R_f=0.8

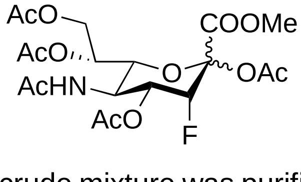

Methyl (5-acetamido-2,4,7,8-tetra-*O*-acetyl-3-fluoro-3,5-dideoxy-5-D-galacto)onate (16)

14 (90 mg, 226 μmol) was dissolved in DMF (0.5 mL) and H_2O (0.5 mL). Selectfluor (4.2 eq.) was added, and the mixture was stirred overnight at 50°C. The solvent was concentrated, after which the crude mixture was purified using flash column chromatography (0 to 100% EtOAc in heptane). Fractions containing the product were concentrated and the product was dissolved in pyridine (2 mL). Ac_2O (1 mL) was added, and the mixture was stirred overnight. The solvent was concentrated, the residue was dissolved in EtOAc and washed with 1M HCl (3x). The aqueous layer was washed with DCM, and the organic layers were combined, dried with $MgSO_4$ and concentrated. The crude product was purified using flash column chromatography (0 to 100% EtOAc in heptane), yielding the product (21% over two steps)

^{1}H NMR (500 MHz, $CDCl_3$) δ 5.52 – 5.45 (m, 1H, H-4), 5.27 (ddd, *J* = 7.7, 5.6, 2.2 Hz, 1H, H-7), 4.93 (dd, *J* = 49.0, 2.6 Hz, 1H, H-3), 4.37 (q, *J* = 10.5 Hz, 1H, H-5), 4.30 – 4.17 (m, 2H, H-8; H-8), 4.11 – 4.08 (m, 1H, H-6), 3.85 (s, 3H, OCH_3), 2.17 (s, 3H, CH_3, Ac), 2.17 (s, 3H, CH_3, Ac), 2.13 (s, 3H, CH_3, Ac), 2.01 (s, 3H, CH_3, Ac), 1.92 (s, 3H, CH_3, Ac). **^{13}C NMR** (126 MHz, $CDCl_3$) δ 170.94 (CO, Ac), 170.76 (CO, Ac), 170.56 (CO, Ac), 170.19 (CO, Ac), 166.92

(CO, Ac), 165.01 (C-1), 95.28 (d, J = 28.8 Hz, C-2), 87.02 (d, J = 185.7 Hz, C-3), 71.98 (C-7), 68.59 (d, J = 17.3 Hz, C-4), 67.18 (C-7), 62.30 (C-8), 53.52 (OCH_3), 45.51 (H-5), 20.97 (CH_3, Ac), 20.69 (CH_3, Ac), 20.66 (CH_3 ,Ac), 20.53 (CH_3, Ac). **^{19}F NMR** (470 MHz, $CDCl_3$) δ _-208.41 (dd, J = 48.8, 27.7 Hz). **HRMS** (ESI-TOF) (*m/z*): $[M+Na]^+$ calcd. For $C_{19}H_{26}FNO_{12}$, 502.13367 found 502.13303. **TLC** (EtOAc) R_f = 0.5

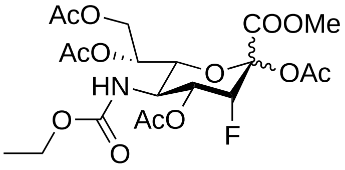

Methyl (5-ethylcarbamado-2,4,7,8-tetra-*O*-acetyl-3-fluoro-3,5-dideoxy-5-D-galacto)onate (17)

15 (47.1 mg, 109 µmol) was dissolved in DMF (1 mL) and H_2O (0.3 mL). Selectfluor (4 eq.) was added, and the mixture was stirred overnight at 50°C. The solvent was concentrated, after which the crude mixture was dissolved in pyridine (2 mL). Ac_2O (1 mL) was added, and the mixture was stirred overnight. The solvent was concentrated, the residue was dissolved in EtOAc and washed with 1M HCl (3x). The aqueous layer was washed with DCM, and the organic layers were combined, dried with $MgSO_4$ and concentrated. The crude product was purified using flash column chromatography (0 to 100% EtOAc in heptane), yielding the product (21% over two steps)

^{1}H NMR (500 MHz, $CDCl_3$) δ 5.61 – 5.40 (m, 2H), 5.34 (ddd, J = 7.6, 5.8, 2.0 Hz, 1H), 5.04 – 4.89 (m, 1H), 4.31 (dd, J = 11.4, 5.7 Hz, 1H), 4.23 (dd, J = 11.4, 7.1 Hz, 1H), 4.16 – 4.05 (m, 4H), 3.87 (s, 3H), 2.19 (s, 3H), 2.18 (s, 3H), 2.15 (s, 3H), 2.03 (s, 3H), 1.26 – 1.21 (m, 3H). **^{13}C NMR** (126 MHz, $CDCl_3$) δ 170.53, 170.52, 170.47, 166.97, 165.04, 95.35, 95.12, 87.82, 86.35, 72.19, 68.62, 68.47, 68.06, 67.33, 53.51, 46.82, 20.95, 20.76, 20.66, 20.55, 14.45. **^{19}F NMR** (377 MHz, $CDCl_3$) δ -208.58 (dd, J = 49.1, 27.9 Hz). **HRMS** (ESI-TOF) (*m/z*): $[M+Na]^+$ calcd. for $C_{20}H_{28}FNO_{13}S$: 532.14424; found, 532.14500. **TLC** (EtOAc/Heptane, 5/5, v/v) R_f = 0.1

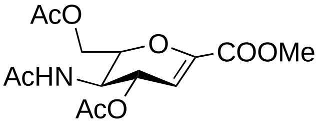

Methyl (5-acetemido-4,7-di-O-acetyl-3-dehyrdo-2,3,5-trideoxy-5-D-galacto)onate (18)

To a solution of sialic acid glucal (**13**) (132.9 mg, 436 µmol) in anhydrous MeOH (0.05M), $NaIO_4$ (2.6 eq.) was added. The reaction mixture was stirred at rt overnight and was filtered through a celite pad. The pad was washed with MeOH and the filtrate was evaporated under reduced pressure. The residue was dissolved in anhydrous MeOH (0.05M), cooled to 0°C and $NaBH_4$ (5.5 eq.) was added. The reaction mixture was stirred for 1 h after which it was filtered through a celite pad. The pad was washed with MeOH and filtrate was evaporated under reduced pressure. The product was purified using flash column chromatography (0 to 20% MeOH in DCM) giving the product, which was dissolved in pyridine (44 eq.), after which Ac_2O (22 eq.) was added. The mixture was stirred overnight at rt. The solvent was concentrated, the residue was dissolved in EtOAc and washed with 1M HCl (3x). The aqueous layer was washed with DCM, and the organic layers were combined, dried with MgSO4 and concentrated. The crude product was purified using flash column chromatography (0 to 100% EtOAc in heptane), yielding the product (66% over three steps)

^{1}H NMR (400 MHz, $CDCl_3$) δ 6.08 (dd, J = 4.0, 0.6 Hz, 1H), 5.86 (d, J = 8.6 Hz, 1H), 5.30 (ddd, J = 5.0, 4.0, 0.8 Hz, 1H), 4.44 (dq, J = 6.4, 0.9 Hz, 2H), 4.36 (dt, J = 8.7, 5.2 Hz, 1H), 4.27 – 4.19 (m, 1H), 3.81 (s, 3H), 2.09 (s, 3H, CH3), 2.06 (s, 3H, CH_3), 1.97 (s, 3H, CH_3). **^{13}C NMR** (400 MHz, $CDCl_3$) δ 170.71, 170.18, 169.87, 161.97, 144.64, 106.94, 76.25, 66.12, 61.49, 52.70, 46.90, 23.13, 20.86, 20.81. **HRMS** (ESI-TOF) (m/z): [M+Na]+ calcd. For $C_{14}H_{19}NO_8Na$, 352.10084 found 352.10093. **TLC** (EtOAc) R_f = 0.6

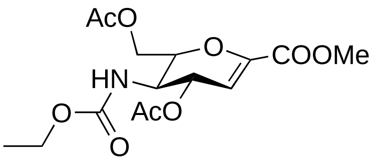

Methyl (5- ethylcarbamado-4,7-di-*O*-acetyl-3-dehyrdo-2,3,5 trideoxy-5-L-arabino)onate (19)

18 (269 mg, 817 µmol) was dissolved in anhydrous DCM (0.1M) and cooled to 0°C under inert atmosphere. 2-fluorpyridine (4 eq.) was added, followed by Tf_2O (1.5 eq.). After 1 h, the mixture was warmed to rt and 1,2-propanediol (4 eq.) was added. After 1h, the mixture was cooled to 0°C, followed by addition of DIPEA (3 eq.) and ethyl chloroformate (3 eq.). The reaction mixture was stirred overnight. The solvent was evaporated under reduced pressure and the crude product was purified using flash column chromatography (0 to 60% EtOAc in Heptane) giving the desired product (39%)

^{1}H NMR (500 MHz, $CDCl_3$) δ 6.01 (d, *J* = 3.7 Hz, 1H, H-3), 5.37 (dd, *J* = 6.0, 3.7 Hz, 1H, H-4), 5.12 (d, *J* = 8.9 Hz, 1H, NH), 4.42 (d, *J* = 8.8 Hz, 2H, H-6; H-7), 4.26 (t, *J* = 7.4 Hz, 1H, H-7), 4.12 – 4.00 (m, 3H, CH_2, Etoc), H-5), 3.78 (s, 3H, OCH_3), 2.06 (s, 3H, CH_3, Ac), 2.04 (s, 3H, CH_3,Ac),1.20–1.15(m,3H,CH_3,Etoc).**^{13}C NMR** (126 MHz, $CDCl_3$) δ 170.70 (CO, Ac), 170.24 (CO, Ac), 161.92 (C-1) , 155.76 (CO, Poc), 144.76 (C-2), 107.14 (C-3), 76.24 (C-6), 66.83 (C-4), 61.67 (C-7), 61.44 (CH_2, Etoc), 52.61 (OCH_3), 48.55 (C-5), 20.83 (CH_3, Ac), 20.76 (CH_3, Ac), 14.45 (CH_3, Etoc). **HRMS** (ESI-TOF) (*m/z*): $[M+Na]^+$ calcd. For $C_{15}H_{21}NO_9$, 382.11140. Found 382.11248. **TLC** (EtOAc) R_f =0.8

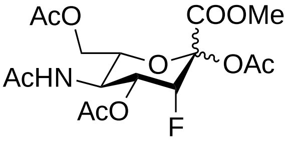

Methyl (5-acetamido-2,4,7-tri-*O*-acetyl-3-fluoro-3,5-dideoxy-5-L-arabino)onate (20)

18 (115 mg, 349 µmol) was dissolved in DMF (2.5 mL) and H_2O (0.8 mL). Selectfluor (4 eq.) was added, and the mixture was stirred overnight at 50°C. The solvent was concentrated, after which the crude mixture was dissolved in pyridine (2 mL). Ac_2O (1 mL) was added, and the mixture was stirred overnight. The solvent was concentrated, the residue was dissolved in EtOAc and washed with 1M HCl (3x). The aqueous layer was washed with DCM, and the organic layers were combined, dried with $MgSO_4$ and concentrated. The crude product was purified using flash column chromatography (0 to 100% EtOAc in heptane), yielding the product (33% over two steps)

^{1}H NMR (500 MHz, $CDCl_3$) δ 5.55 (d, *J* = 9.1 Hz, 1H), 5.45 (ddd, *J* = 28.0, 11.2, 2.6 Hz, 1H), 4.93 (dd, J = 49.0, 2.6 Hz, 1H), 4.36 – 4.21 (m, 3H), 4.08 – 4.02 (ddd, J = 10.4, 5.4, 2.1 Hz, 1H), 3.86 (s, 3H), 2.16 (s, 3H), 2.14 (s, 3H), 2.09 (s, 3H), 1.96 (s, 3H). **^{13}C NMR** (500 MHz, $CDCl_3$) 171.19, 171.02, 170.40, 167.08, 165.18, 95.26, 87.11, 72.57, 68.28, 62.74, 53.61, 46.55, 20.85, 20.81, 20.73, 20.55. **^{19}F NMR** (470 MHz, $CDCl_3$) δ _-208.04 (dd, *J* = 49.0, 28.0 Hz). **HRMS** (ESI-TOF) (*m/z*): $[M+Na]^+$ calcd. For $C_{16}H_{22}FNO_{10}$: 430.11254; found 430.11428. **TLC** (DCM/MeOH, 8/2, v/v) R_f = 0.2

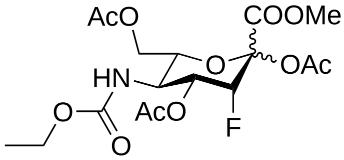

Methyl (5-ethylcarbamado-2,4,7-tri-*O*-acetyl-3-fluoro-3,5-dideoxy-5-L-arabino)onate (21)

19 (41.7 mg, 116 µmol) was dissolved in DMF (2.5 mL) and H_2O (0.8 mL). Selectfluor (4 eq.) was added, and the mixture was stirred overnight at 50°C. The solvent was concentrated, after which the crude mixture was dissolved in pyridine (2 mL). Ac_2O (1 mL) was added, and the mixture was stirred overnight. The solvent was concentrated, the residue was dissolved in EtOAc and washed with 1M HCl (3x). The aqueous layer was washed with DCM, and the

organic layers were combined, dried with $MgSO_4$ and concentrated. The crude product was purified using flash column chromatography (0 to 100% EtOAc in heptane), yielding the product (14% over two steps)
^{1}H NMR (500 MHz, $CDCl_3$) δ 5.45 (dd, *J* = 27.9, 10.3 Hz, 1H, H-4), 4.95 (dd, *J* = 49.1, 2.6 Hz, 1H, H-3), 4.40 – 4.27 (m, 2H, H-7; H-7), 4.14 – 4.02 (m, 4H, H-5; H-6; CH_2, Etoc), 3.85 (s, 3H, OCH_3), 2.17 (s, 3H, CH_3, Ac), 2.14 (s, 3H, CH_3, Ac), 2.09 (s, 3H, CH_3, Ac), 1.23 (t, *J* = 7.0 Hz, 3H, CH_3, Etoc). **^{13}C NMR** (126 MHz, $CDCl_3$) δ 87.21 (d, *J* = 184.7 Hz, C-3), 72.40 (C-6), 68.23 (d, *J* = 21.4 Hz, C-4), 62.63 (C-7), 61.52 (CH_2, Etoc), 53.59 (OCH_3), 47.78 (C-5), 20.82 (CH_3, Ac), 20.70 (CH_3, Ac), 20.58 (CH_3, Ac), 14.47 (CH_3, Etoc). **^{19}F NMR** (470 MHz, $CDCl_3$) δ _ -208.33 (dd, *J* = 49.5, 27.8 Hz). **HRMS** (ESI-TOF) (*m/z*): $[M+Na]^+$ calcd. For $C_{17}H_{24}FNO_{11}$, 460.12311 found 460.12345
TLC (Toluene/EtOAc, 5/5, v/v) R_f = 0.4

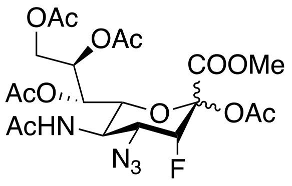

Methyl (5-acetamido-4-azido-7,8,9-tri-O-acetyl-3-dehydro-3,5-dideoxy-3-fluoro-5-β-D-glycero-D-galacto)onate (23)

22 (937 mg, 2.05 mmol) was dissolved in DMF (15 mL) and H_2O (5 mL). Selectfluor (4 eq.) was added, and the mixture was stirred overnight at 50°C. The solvent was concentrated, after which the crude mixture was dissolved in pyridine (7.3 mL). Ac_2O (1 mL) was added, and the mixture was stirred overnight. The solvent was concentrated, the residue was dissolved in EtOAc and washed with 1M HCl (3x). The aqueous layer was washed with DCM, and the organic layers were combined, dried with $MgSO_4$ and concentrated. The crude product was purified using flash column chromatography (0 to 100% EtOAc in heptane), yielding the product (593 mg, 54% over two steps)

^{1}H NMR (500 MHz, $CDCl_3$) δ 6.07 (d, *J* = 7.7 Hz, 1H), 5.32 (dd, *J* = 6.3, 1.7 Hz, 1H), 5.20 (td, *J* = 6.0, 2.4 Hz, 1H), 5.03 – 4.87 (m, 1H), 4.84 – 4.59 (m, 1H), 4.49 (dt, *J* = 11.5, 2.3 Hz, 2H), 4.24 (dd, *J* = 12.6, 5.9 Hz, 1H), 3.84 (s, 3H), 3.54 (dd, *J* = 16.9, 7.3 Hz, 1H), 2.18 (s, 3H), 2.17 (s, 3H), 2.05 – 2.02 (m, 9H). **^{13}C NMR** (126 MHz, $CDCl_3$) δ 171.47, 171.21, 170.53, 169.93, 167.12, 165.11, 94.71, 94.48, 89.14, 87.67, 70.42, 70.03, 68.25, 61.99, 56.72, 56.59, 53.50, 23.49, 20.80, 20.78, 20.69, 20.44. **^{19}F NMR** (470 MHz, $CDCl_3$) δ -208.21 (t, *J* = 39.8 Hz). **HRMS** (ESI-TOF) (*m/z*): $[M+Na]^+$ calcd. for $C_{20}H_{27}FN_4O_{12}Na$: 557.1507; found, 557.1510. **TLC** (EtOAc) R_f = 0.5

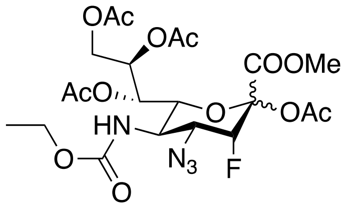

Methyl (5-ethylcarbamado-4-azido-7,8,9-tri-O-acetyl-3-dehydro-3,5-dideoxy-3-fluoro-5-β-D-glycero-D-galacto)onate (24)

23 (39 mg, 73 μmol) was dissolved in anhydrous DCM (0.1M) and cooled to 0°C under inert atmosphere. 2-fluorpyridine (4 eq.) was added, followed by Tf_2O (1.5 eq.). After 1 h, the mixture was warmed to rt and 1,2-propanediol (4 eq.) was added. After 1h, the mixture was cooled to 0°C, followed by addition of DIPEA (3 eq.) and ethyl chloroformate (3 eq.). The reaction mixture was stirred overnight. The solvent was evaporated under reduced pressure and the crude product was purified using flash

column chromatography (0 to 60% EtOAc in Heptane) giving the desired product (11.7 mg, 28%)

^{1}H NMR (500 MHz, $CDCl_3$) δ 5.38 – 5.33 (m, 1H), 5.23 (dd, *J* = 6.0, 2.4 Hz, 1H), 5.16 (d, *J* = 7.8 Hz, 1H), 5.03 – 4.88 (m, 1H), 4.62 – 4.46 (m, 2H), 4.33 (d, *J* = 10.3 Hz, 1H), 4.25 (dd, *J* = 12.6, 6.0 Hz, 1H), 4.20 – 4.06 (m, 3H), 3.84 (s, 3H), 3.44 (q, *J* = 10.0 Hz, 1H), 2.18 – 2.16 (m, 6H), 2.05 (s, 3H), 2.04 (s, 3H), 1.27 (t, *J* = 7.2 Hz, 3H).
^{13}C NMR (126 MHz, $CDCl_3$) δ 170.99, 170.54, 169.87, 167.09, 165.14, 155.69, 94.69, 94.46, 89.27, 87.79, 70.67, 70.42, 68.33, 62.10, 61.59, 57.11, 56.97, 53.51, 47.90, 20.79, 20.77, 20.72, 20.50, 14.47. **^{19}F NMR** (470 MHz, $CDCl_3$) δ -207.81 (dd, *J* = 48.2, 30.2 Hz). **HRMS** (ESI-TOF) (*m/z*): $[M+Na]^+$ calcd. for $C_{21}H_{29}FN_4O_{13}Na$: 587.1613; found, 587.1601. **TLC** (EtOAc/Heptane, 7/3, v/v) R_f = 0.5

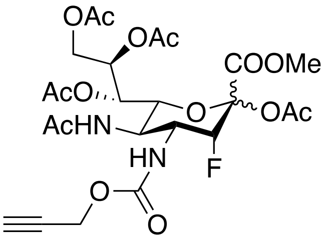

Methyl (5-acetamido-4-propargylcarbamado-7,8,9-tri-O-acetyl-3-dehydro-3,5-dideoxy-3-fluoro-5-β-D-glycero-D-galacto)onate (25)

23 (30 mg, 56 µmol) was dissolved in THF (0.1 M), followed by the addition of PMe_3 (1M in THF, 1 eq.) after TLC showed consumption of the starting material, propargyl chloroformate (1.5 eq.) was added. After 1h, water was added and the aqueous layer was extracted with DCM. The organic layer was dried over $MgSO_4$ and concentrated *in vacuo*. The crude product was purified using flash column chromatography (0-70% EtOAc in heptane) affording the product (7.9 mg, 24%).

^{1}H NMR (500 MHz, $CDCl_3$) δ 5.55 (d, *J* = 9.4 Hz, 1H), 5.44 – 5.36 (m, 2H), 5.08 (ddd, *J* = 6.9, 4.4, 2.5 Hz, 1H), 4.93 – 4.79 (m, 1H), 4.74 – 4.53 (m, 3H), 4.35 – 4.02 (m, 4H), 3.84 (s, 3H), 2.47 (t, *J* = 2.4 Hz, 1H), 2.14 (s, 3H), 2.06 – 2.02 (m, 12H).
^{13}C NMR (126 MHz, $CDCl_3$) δ 171.16, 170.99, 170.60, 170.48, 170.23, 167.04, 165.13, 155.90, 94.92, 94.69, 89.14, 87.70, 75.00, 73.07, 71.76, 68.03, 62.10, 53.51, 51.73, 45.01, 30.92, 23.15, 21.04, 20.92, 20.77, 20.71, 20.53. **^{19}F NMR** (470 MHz, $CDCl_3$) δ -207.20 (dd, *J* = 48.1, 30.9 Hz). **HRMS** (ESI-TOF) (*m/z*): $[M+Na]^+$ calcd. for $C_{24}H_{31}FN_2O_{14}Na$: 613.1657; found, 613.1656. **TLC** (Acetone/CH_2Cl_2, 4/6, v/v) R_f = 0.6

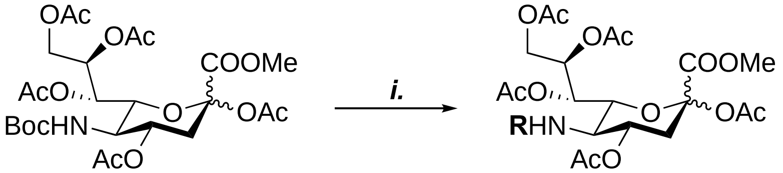

Scheme S1: Synthesis of alkyne derivatives **26** – **28**. *i.)* 1. TFA, H_2O, DCM; 2. For **26**: 4-pentynoic acid, DIC, DMAP, THF, 68% over two steps; For **27**: Compound has been described previously[2]. For **28**: 4-nitrophenyl N-propargyl carbamate, TEA, DCM, 48% over two steps.

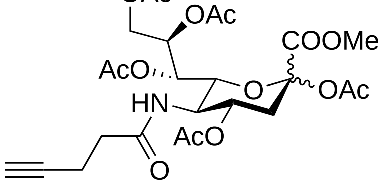

Methyl (5-*N*-4-pentynoyl-2,4,7,8,9-penta-*O*-acetyl-3,5-dideoxy-D-glycero-β-D-galacto-non-2-ulopyranoside)onate (26)

The compound was prepared analogous to **27** as described[2]. To the obtained amine was added a mixture of 4-pentynoic acid (2 eq.) and DIC (2 eq.) in dry THF (0.2M) after which DMAP (0.1 eq.) was added. After 4h, the mixture was diluted with DCM, washed with 1M HCl (2x), sat. $NaHCO_3$ (2x) and brine. The crude product was

purified using flash column chromatography (20% to 50% EtOAc in heptane) to yielding the product (33 mg, 57 µmol, 68%)

^{1}H NMR (500 MHz, $CDCl_3$) (major anomer) δ 5.52 (dd, *J*= 7.8, 2.0 Hz), 5.39 (dd, *J*= 7.3, 2.5 Hz, 1H), 5.19 (ddt, *J*= 7.2, 5.5, 3.3 Hz, 1H), 5.02 (ddd, *J*= 11.7, 10.2, 4.8 Hz, 1H), 4.71 (dd, *J*= 10.7, 2.3 Hz, 1H), 4.34 (dd, *J*= 12.5, 2.6 Hz, 1H), 4.22 – 4.10 (m, 1H) 4.06 (dd, *J*= 12.5, 5.5 Hz, 1H), 3.76 (m, 3H), 2.66 – 2.40 (m, 3H), 2.38 – 2.24 (m, 2H), 2.20 – 1.92 (m, 16H) **^{13}C NMR** (126 MHz, $CDCl_3$) δ 171.16, 170.73, 170.71, 170.02, 169.83, 168.54, 168.21, 95.41, 73.78, 69.77, 69.50, 68.38, 67.37, 62.08, 52.91, 48.91, 36.92, 35.41, 20.96, 14.61. **HRMS** (ESI-TOF) (*m/z*): $[M+Na]^+$ calcd. for $C_{25}H_{33}N_1NaO_{14}$, 594.17987; found, 594.17772. **TLC** (EtOAc/Heptane, 5/5, v/v) R_f = 0.2

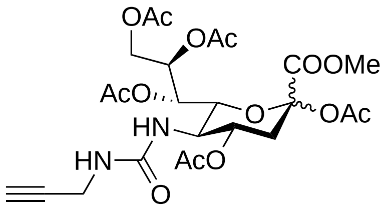

Methyl (5-*N*-propargylaminocarbonyl-2,4,7,8,9-penta-*O*-acetyl-3,5-dideoxy-D-glycero-β-D-galacto-non-2-ulopyranoside)onate (28)

The compound was prepared analogous to **27** as described[2]. The obtained amine was dissolved in DCM (0.04M), to which 4-nitrophenyl N-propargyl carbamate[3] (1.2 eq.) and TEA (2 eq.) were added. The mixture was stirred overnight, after which it was diluted with DCM, washed with 1M HCl (2x), sat. $NaHCO_3$ (2x) and brine. The crude product was purified using flash column chromatography (20% to 50% EtOAc in heptane) to yielding the product (23.3 mg, 41 µmol, 48%).

^{1}H NMR (500 MHz, $CDCl_3$) δ 5.45 (dd, *J*= 4.8, 2.3 Hz, 1H), 5.24 (ddd, *J*= 11.5, 10.2, 5.0 Hz, 1H), 5.08 (ddd, *J*= 7.2, 4.8, 2.6 Hz, 1H), 4.72 (dd, *J*= 6.5, 4.7 Hz, 1H), 4.53 (dd, *J*= 12.4, 2.6 Hz, 1H), 4.43 – 4.35 (m, 1H,), 4.19 – 3.93 (m, 2H), 3.80 (s, 3H), 3.78 – 3.74 (m, 1H) 2.54 (dd, *J*= 13.4, 5.0 Hz, 1H), 2.23 (t, 2.5 Hz), 2.20 – 2.14 (m, 6H), 2.14 – 2.01 (m, 10H). **^{13}C NMR** (126 MHz, $CDCl_3$) δ 171.32, 170.64, 170.43, 170.42, 168.34, 166.46, 156.39, 97.50, 73.33, 71.63, 71.35, 68.74, 68.08, 62.26, 53.18, 50.61, 36.10, 30.21, 20.96. **HRMS** (ESI-TOF) (*m/z*): $[M+Na]^+$ calcd. for $C_{24}H_{32}N_2NaO_{14}$, 595.17512; found, 595.17331. **TLC** (EtOAc/Heptane, 5/5, v/v) R_f = 0.2

Methyl (5-butylamido-4,7,8,9-tetra-O-acetyl-3-dehydro-3,5-dideoxy-3-fluoro-5-β-D-glycero-D-galacto)onate (2)

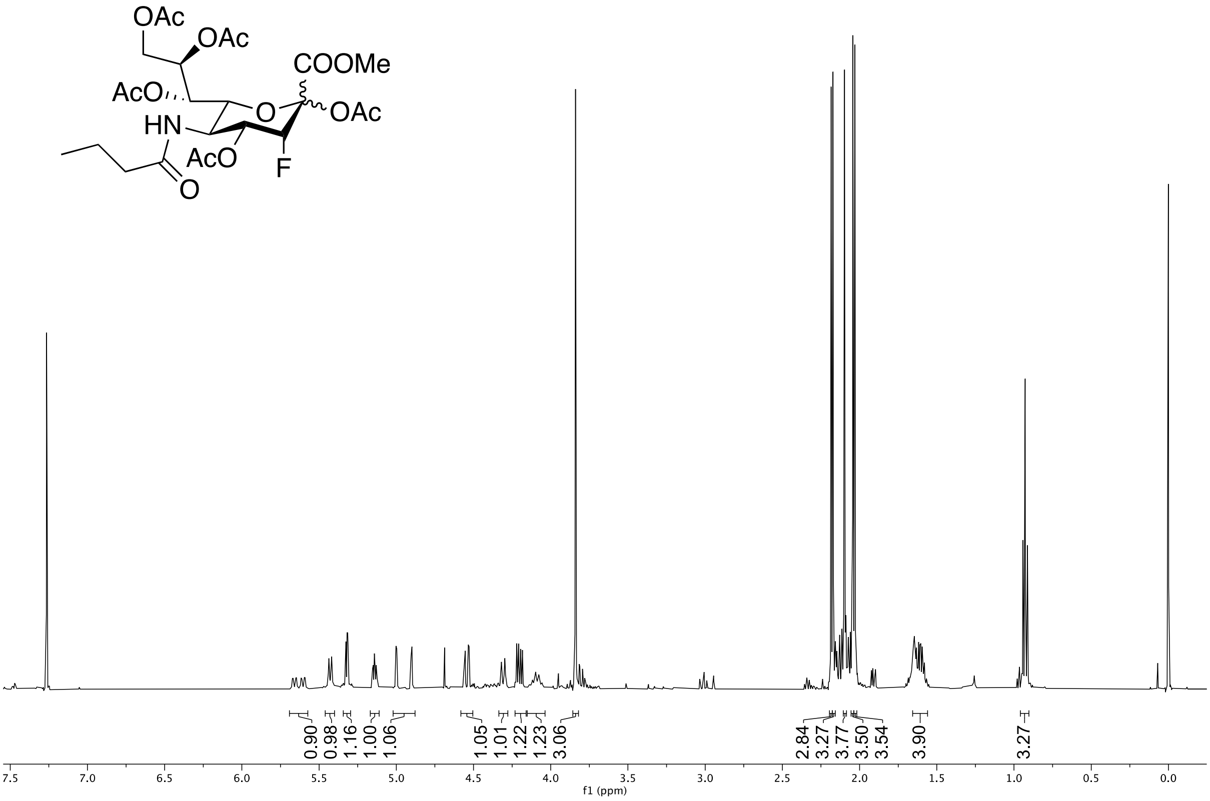

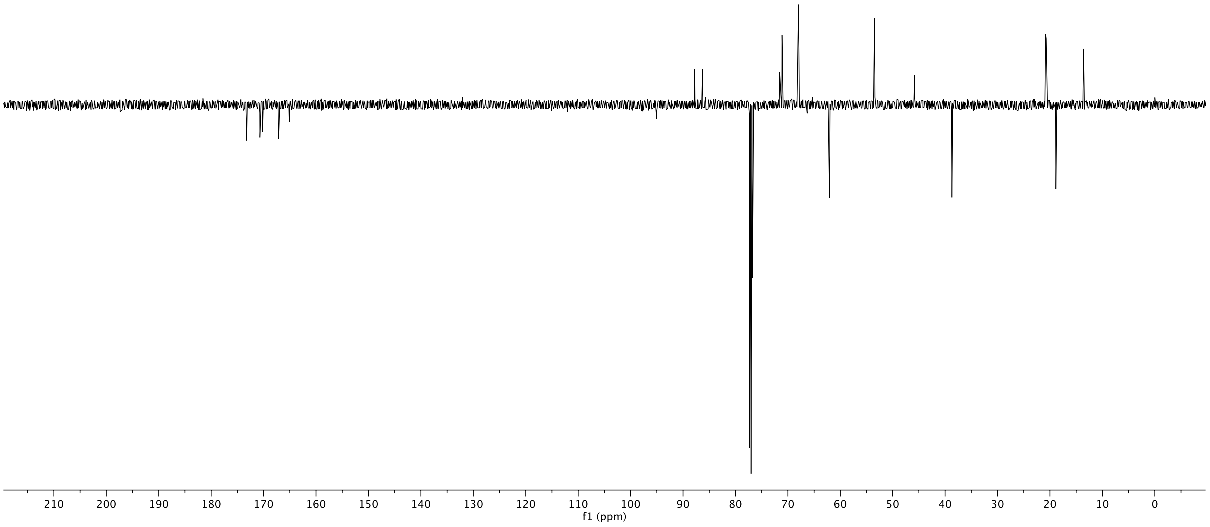

Methyl (5-ethylureido-4,7,8,9-tetra-O-acetyl-3-dehydro-3,5-dideoxy-3-fluoro-5-β-D-glycero-D-galacto)onate (6)

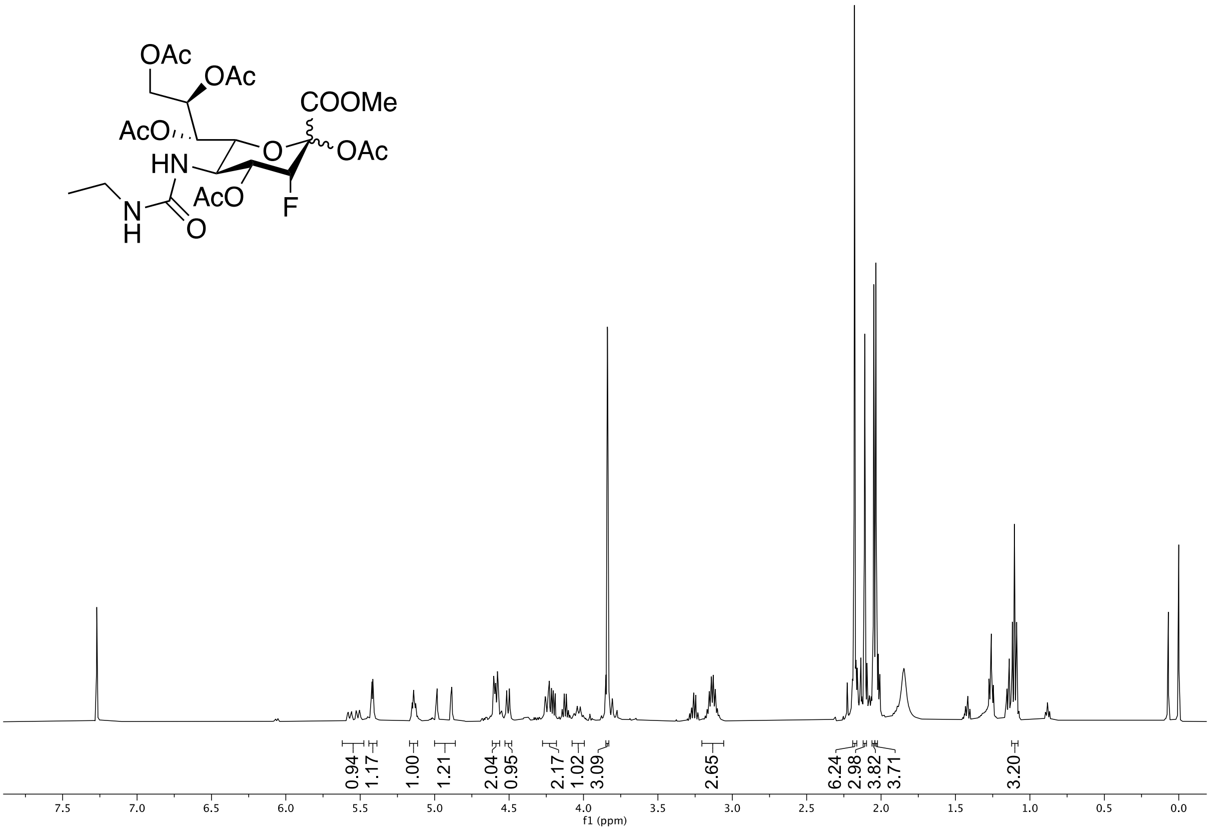

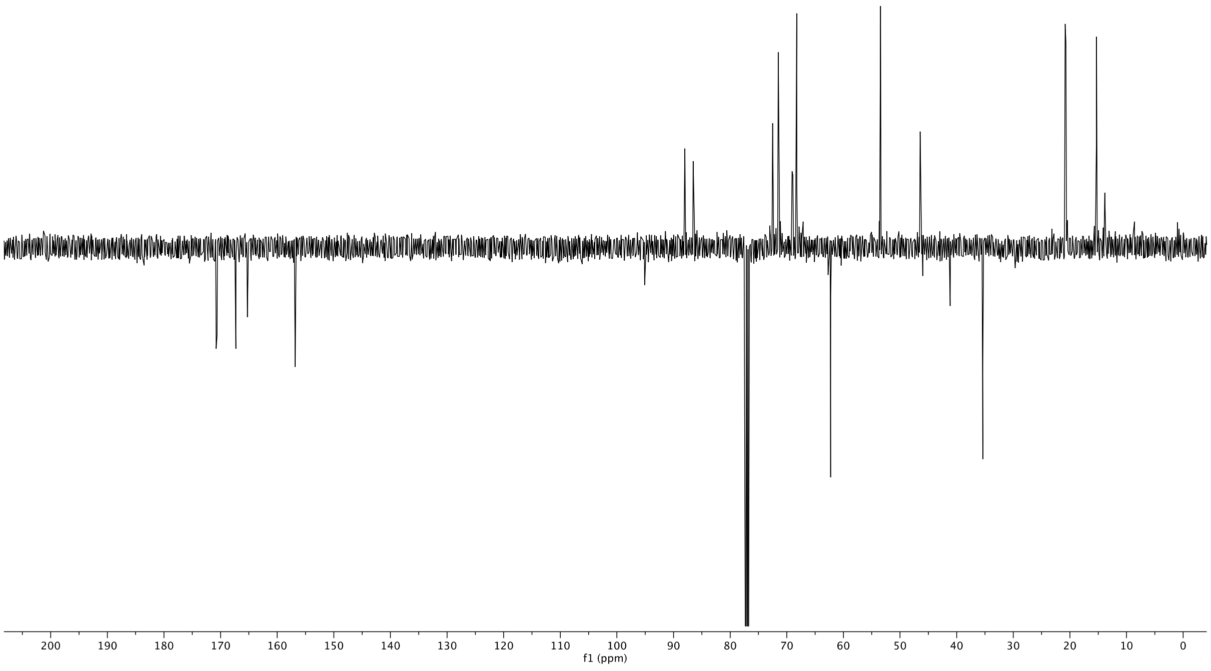

Methyl (5-propargylureido-4,7,8,9-tetra-O-acetyl-3-dehydro-3,5-dideoxy-3-fluoro-5-β-D-glycero-D-galacto)onate (7)

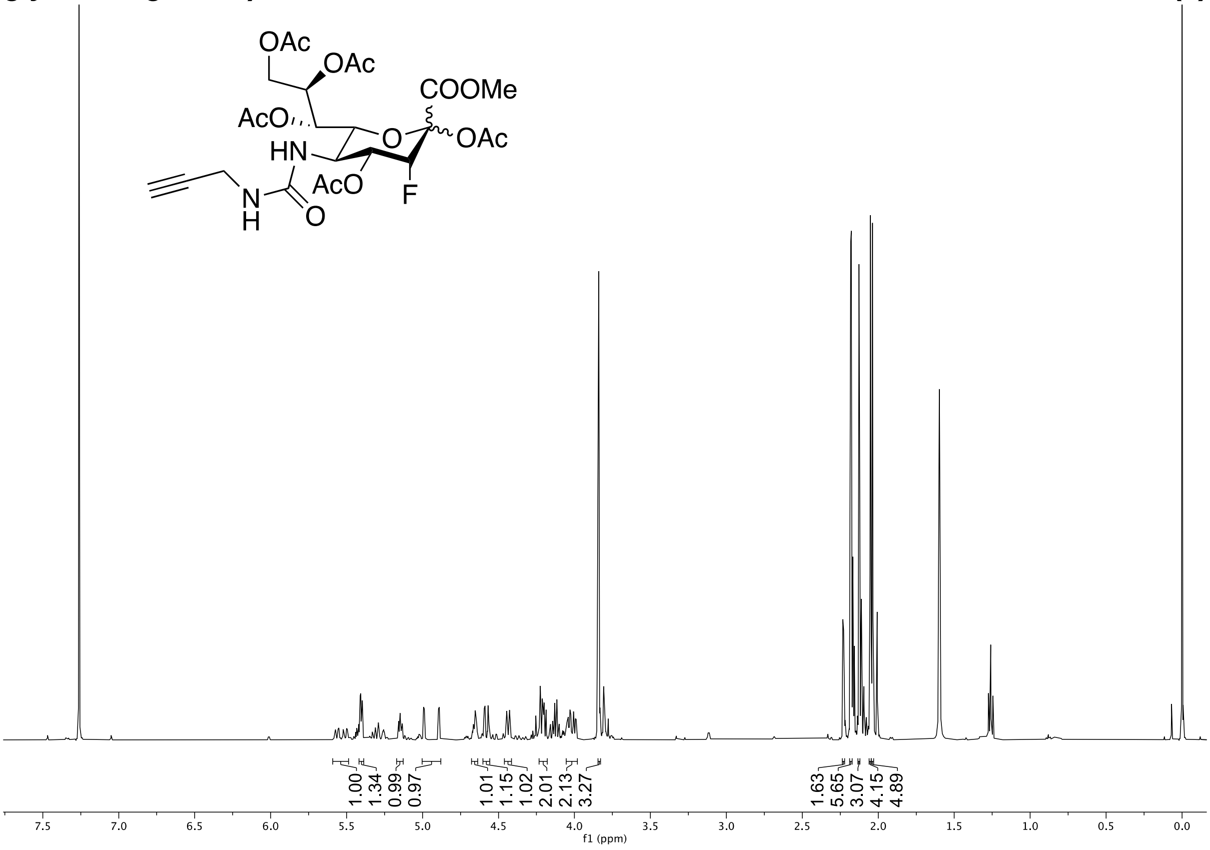

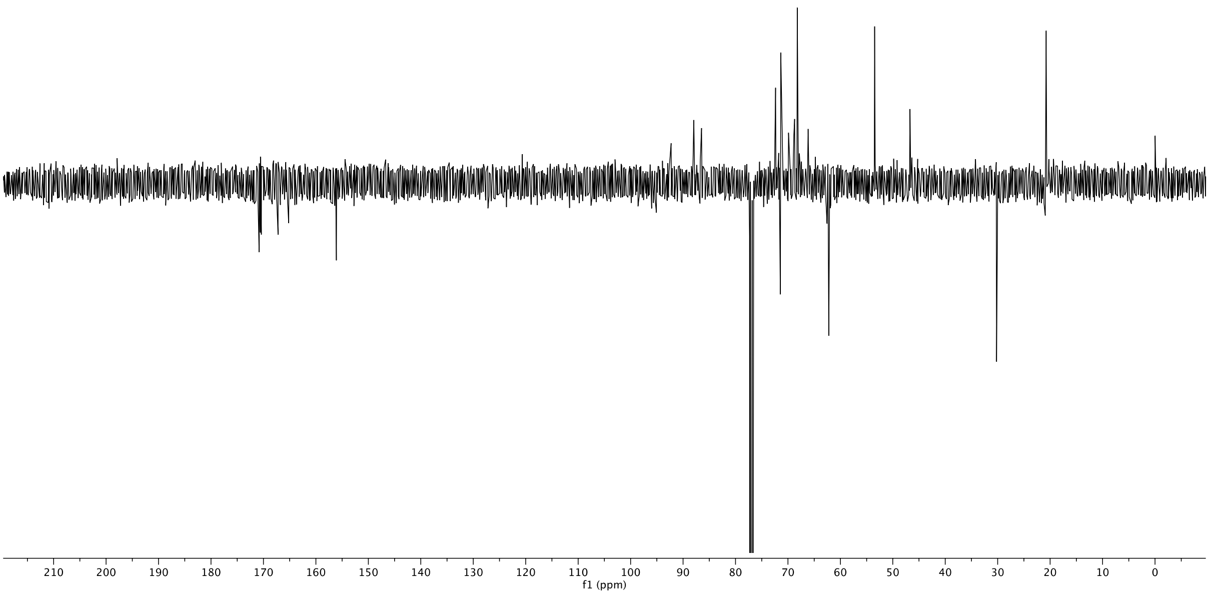

Methyl (5-ethylthioureido-4,7,8,9-tetra-O-acetyl-3-dehydro-3,5-dideoxy-3-fluoro-5-β-D-glycero-D-galacto)onate (8)

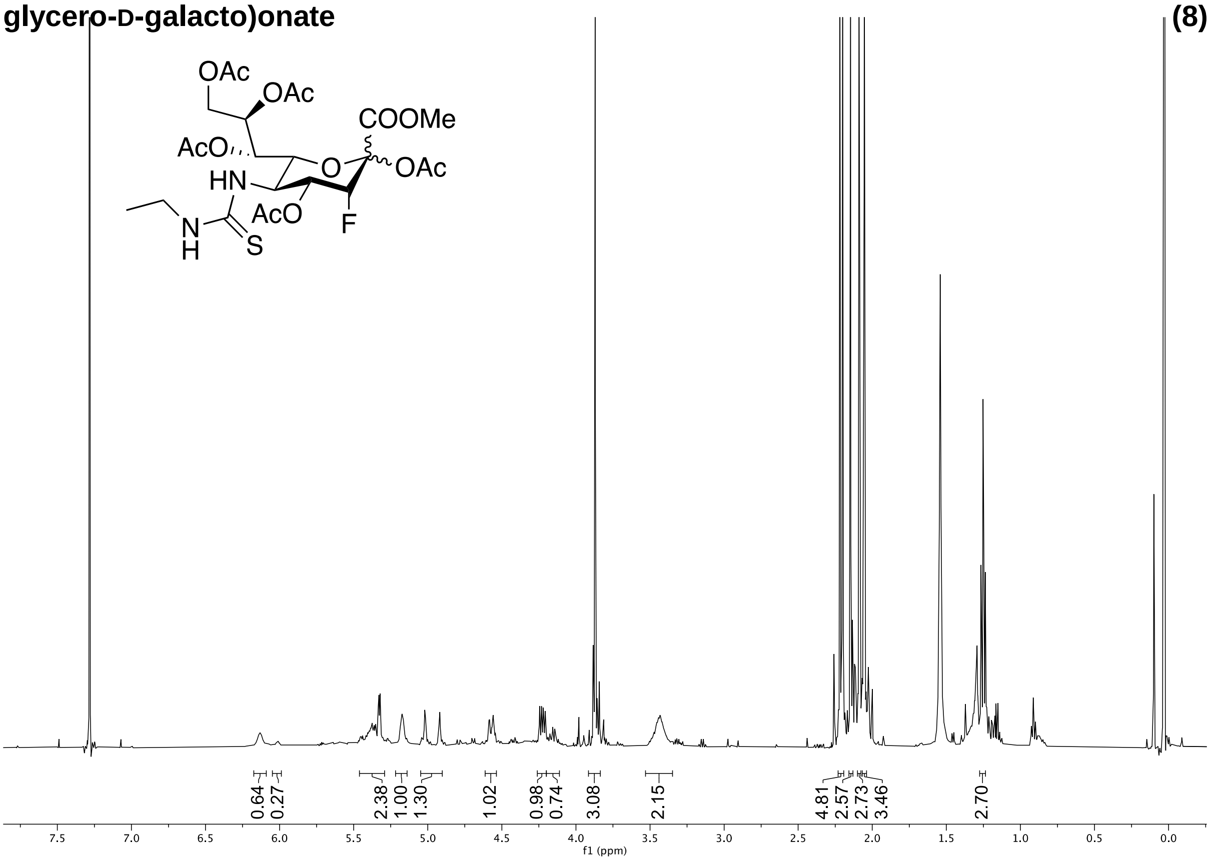

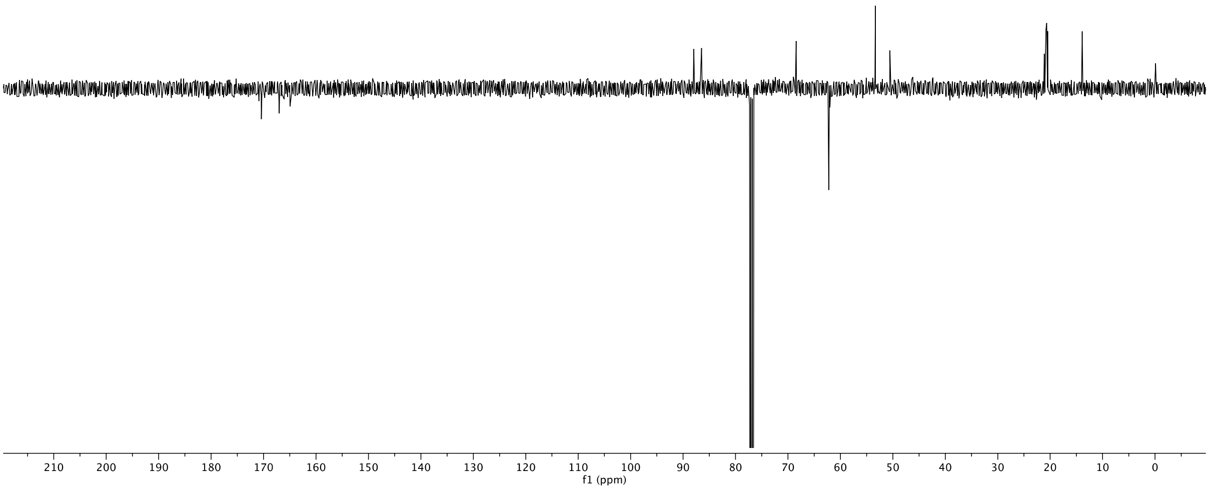

Methyl (5-propargylthioureido-4,7,8,9-tetra-O-acetyl-3-dehydro-3,5-dideoxy-3-fluoro-5-β-D-glycero-D-galacto)onate (9)

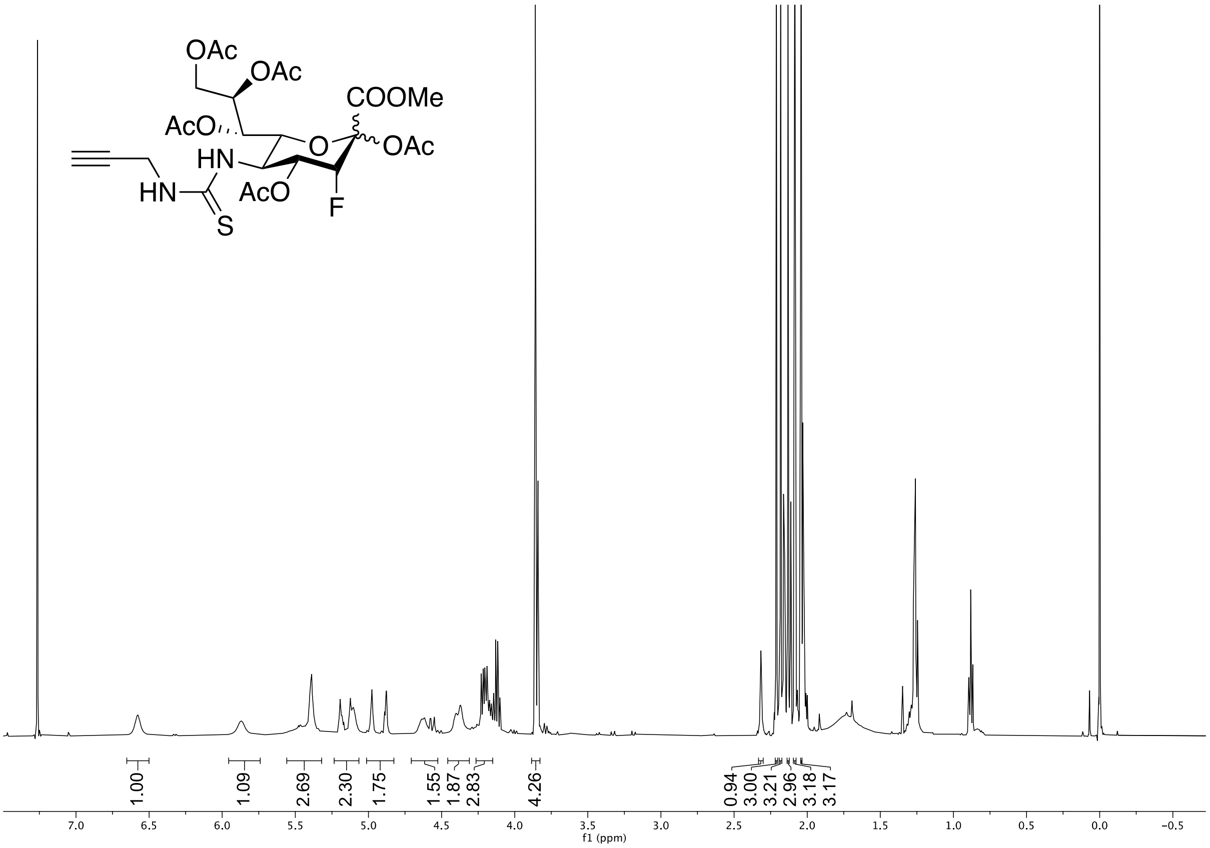

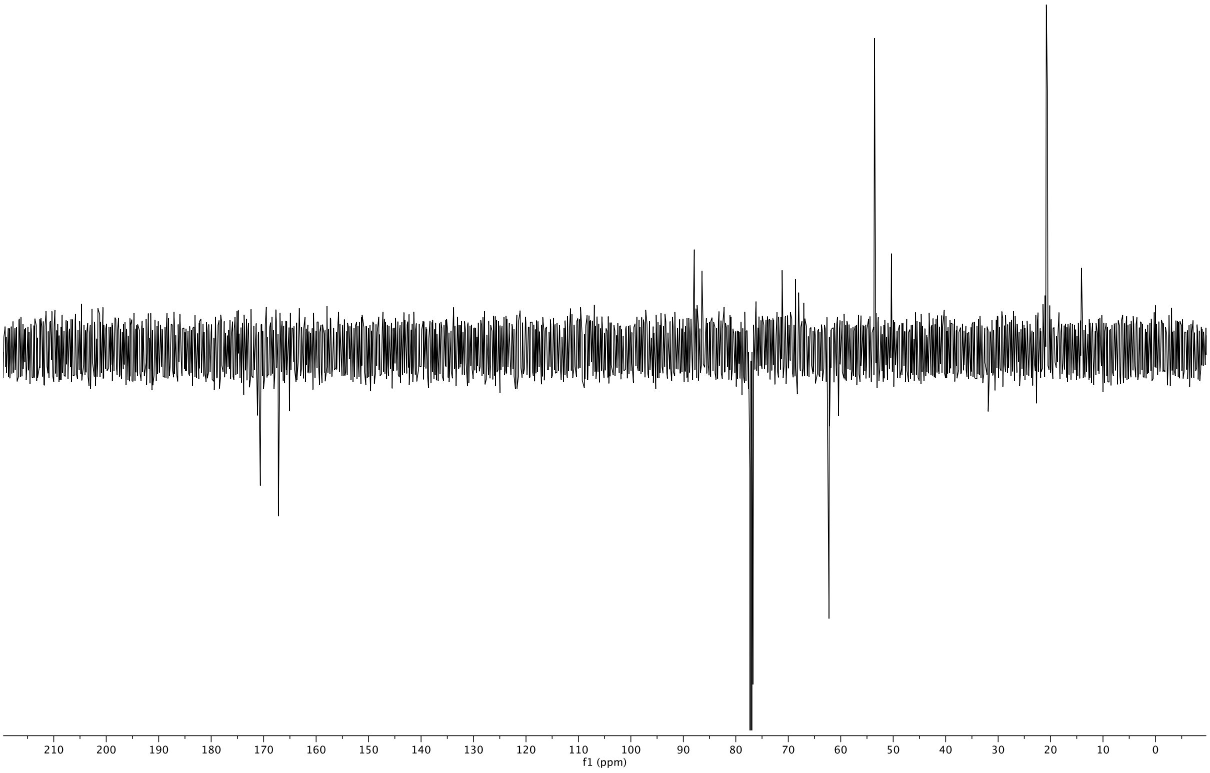

Methyl (5-S-ethylthiocarbamado-4,7,8,9-tetra-O-acetyl-3-dehydro-3,5-dideoxy-3-fluoro-5-β-D-glycero-D-galacto)onate (10)

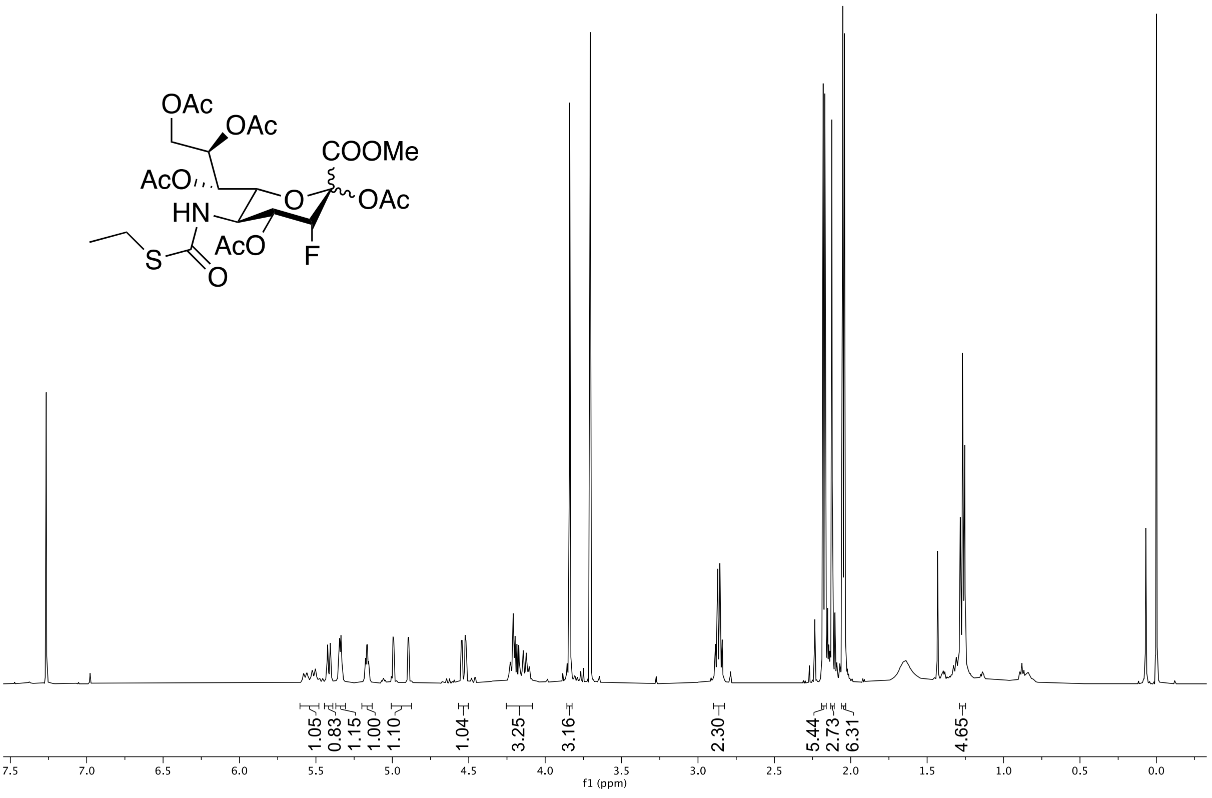

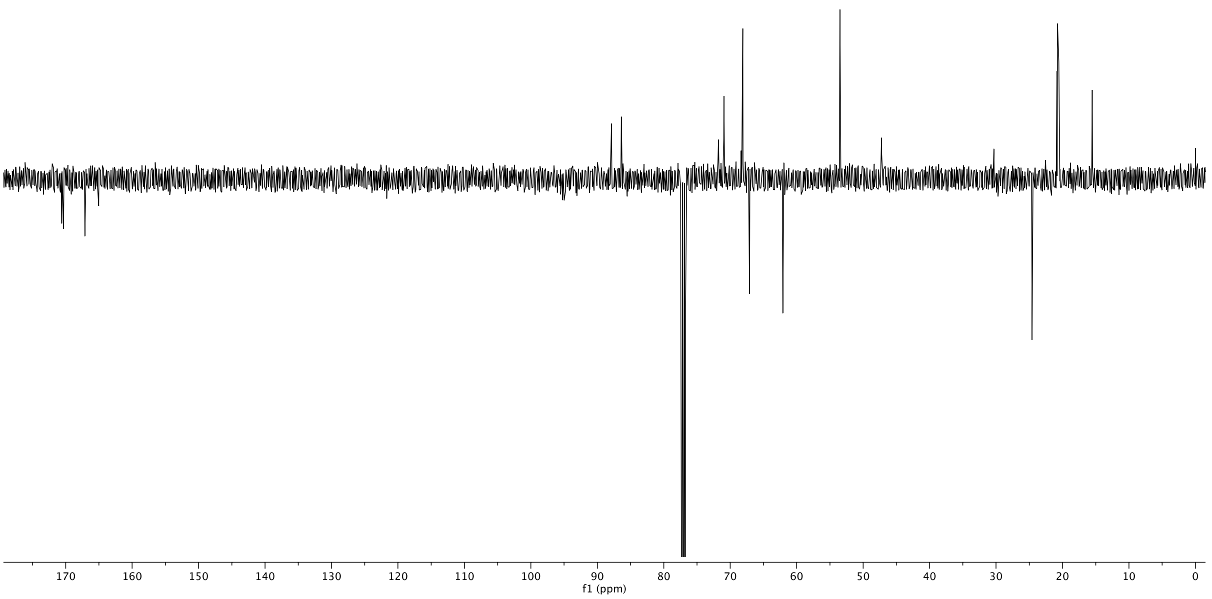

Methyl (5-S-propargylthiocarbamado-4,7,8,9-tetra-O-acetyl-3-dehydro-3,5-dideoxy-3-fluoro-5-β-D-glycero-D-galacto)onate (11)

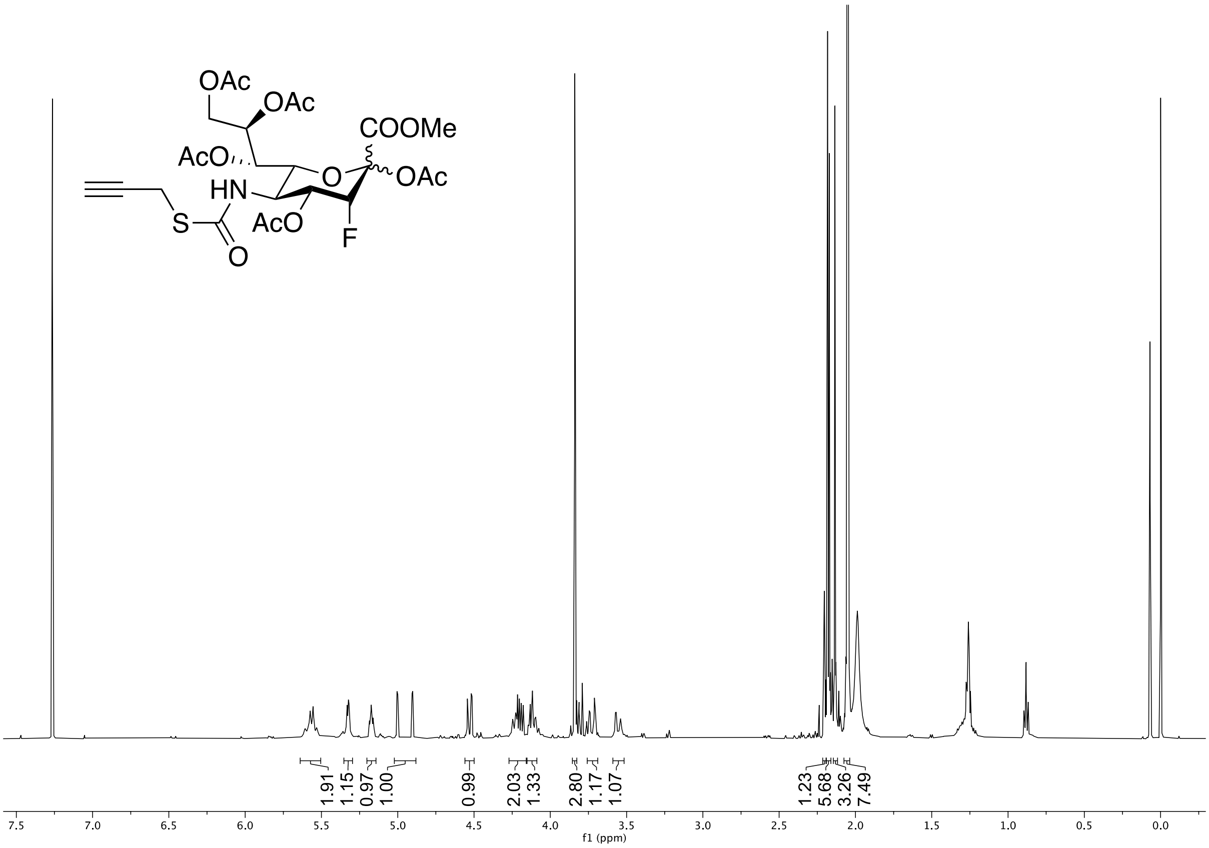

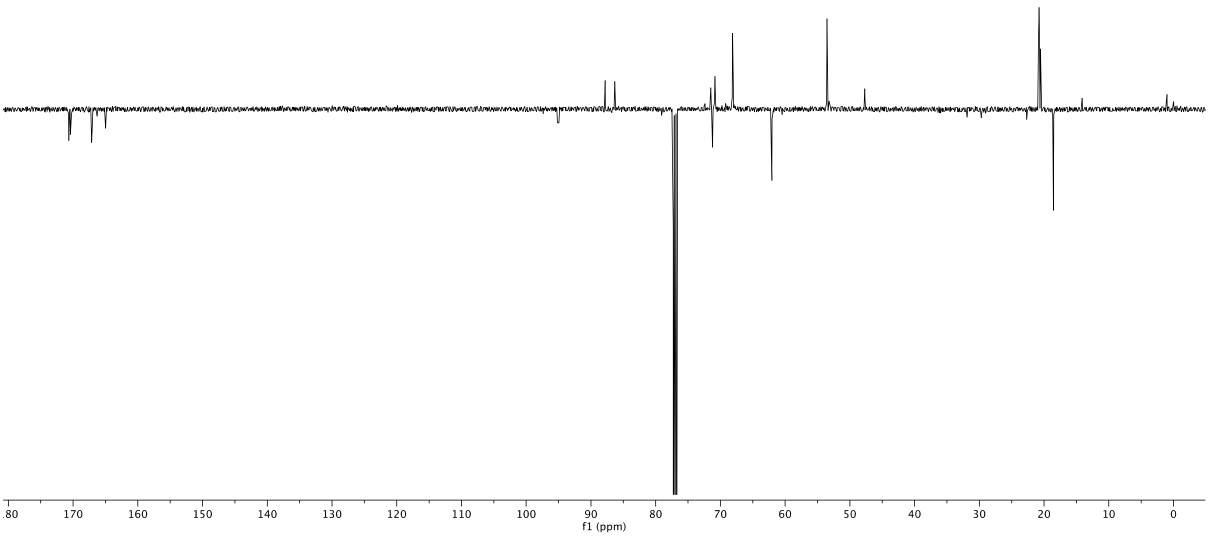

Methyl (5-azidoethylcarbamado-4,7,8,9-tetra-O-acetyl-3-dehydro-3,5-dideoxy-3-fluoro-5-β-D-glycero-D-galacto)onate(12)

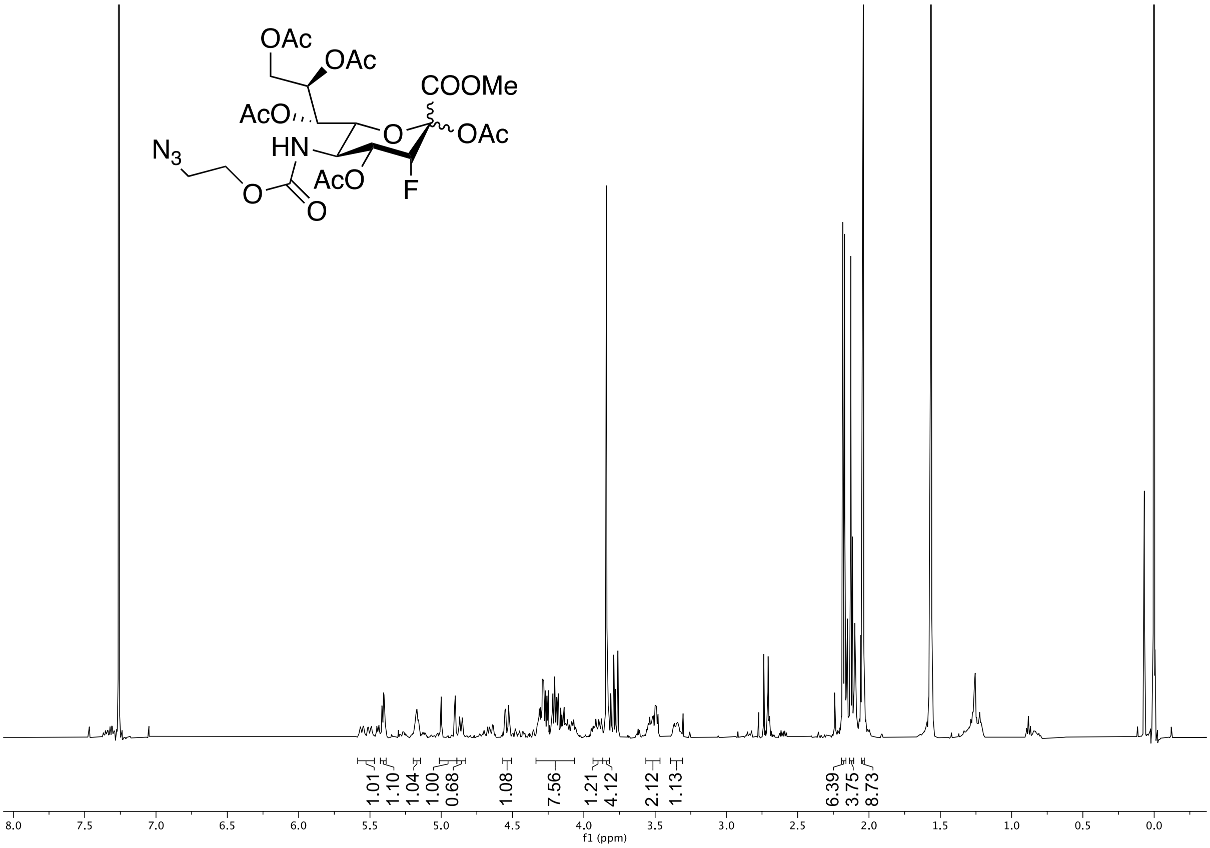

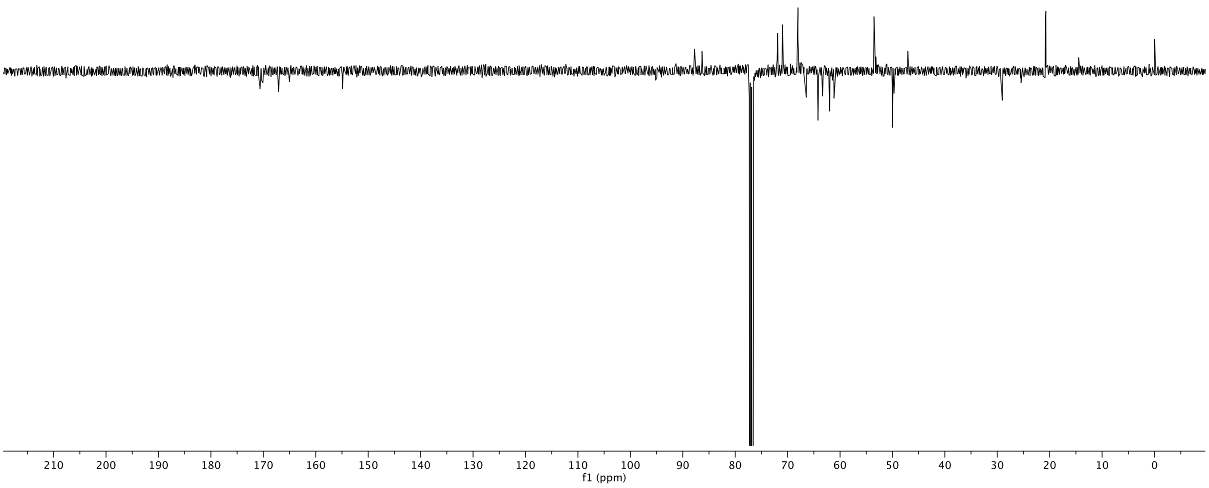

Methyl (5-acetemido-4,7,8-tri-O-acetyl-3-dehyrdo-2,3,5-trideoxy-5-D-galacto)onate (14)

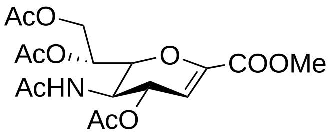

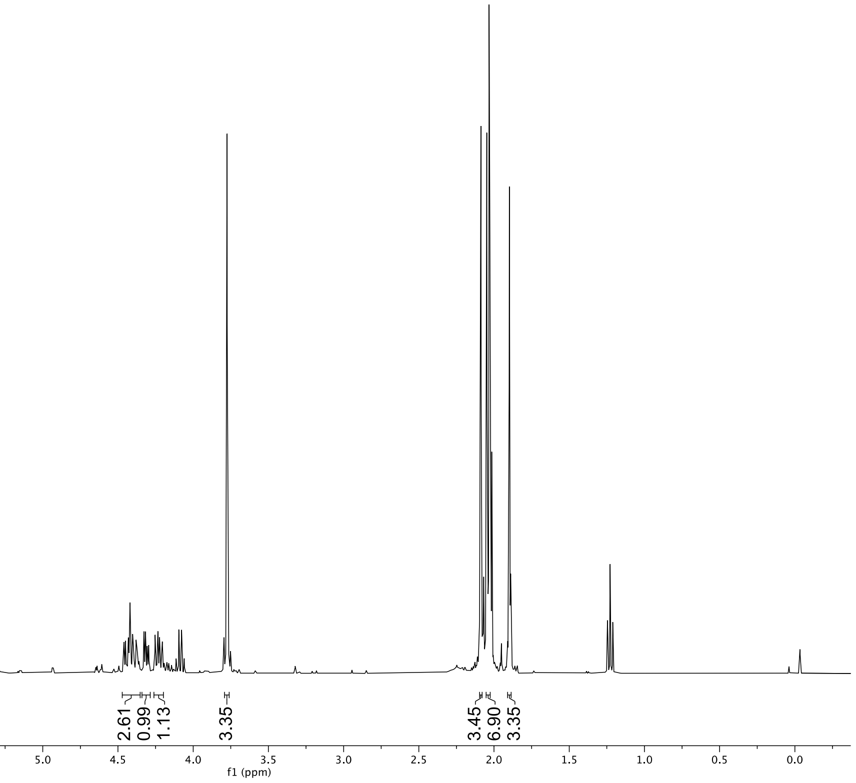

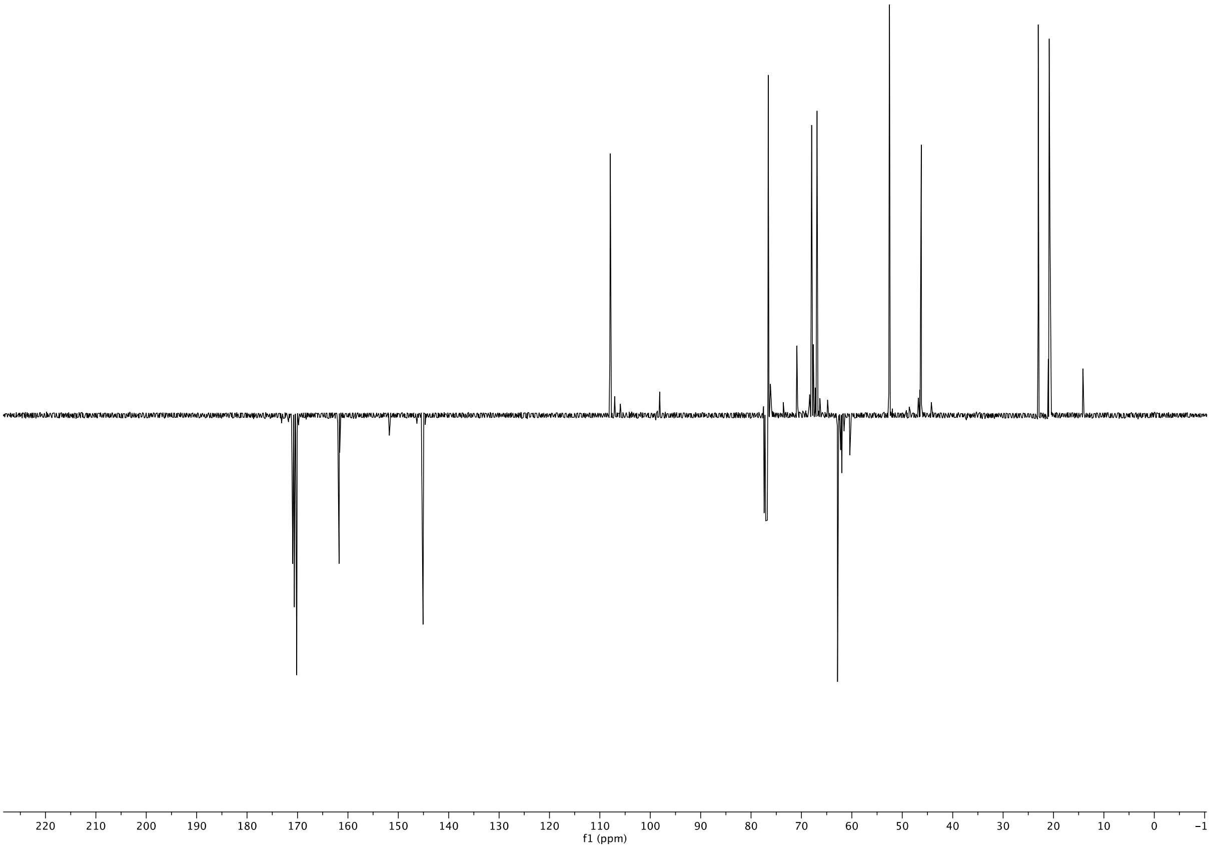

Methyl (5-ethylcarbamado-4,7,8-tri-O-acetyl-3-dehyrdo-2,3,5-trideoxy-5-D-galacto)onate (15)

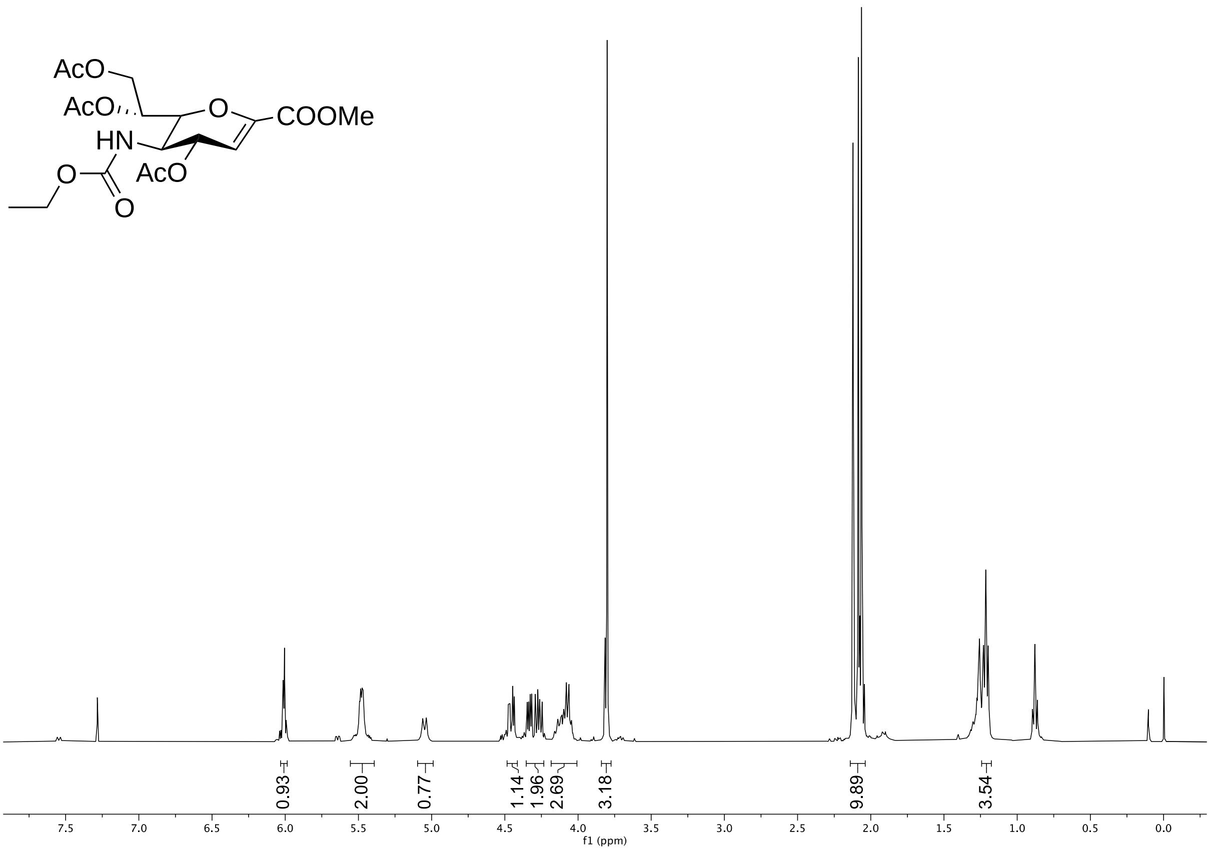

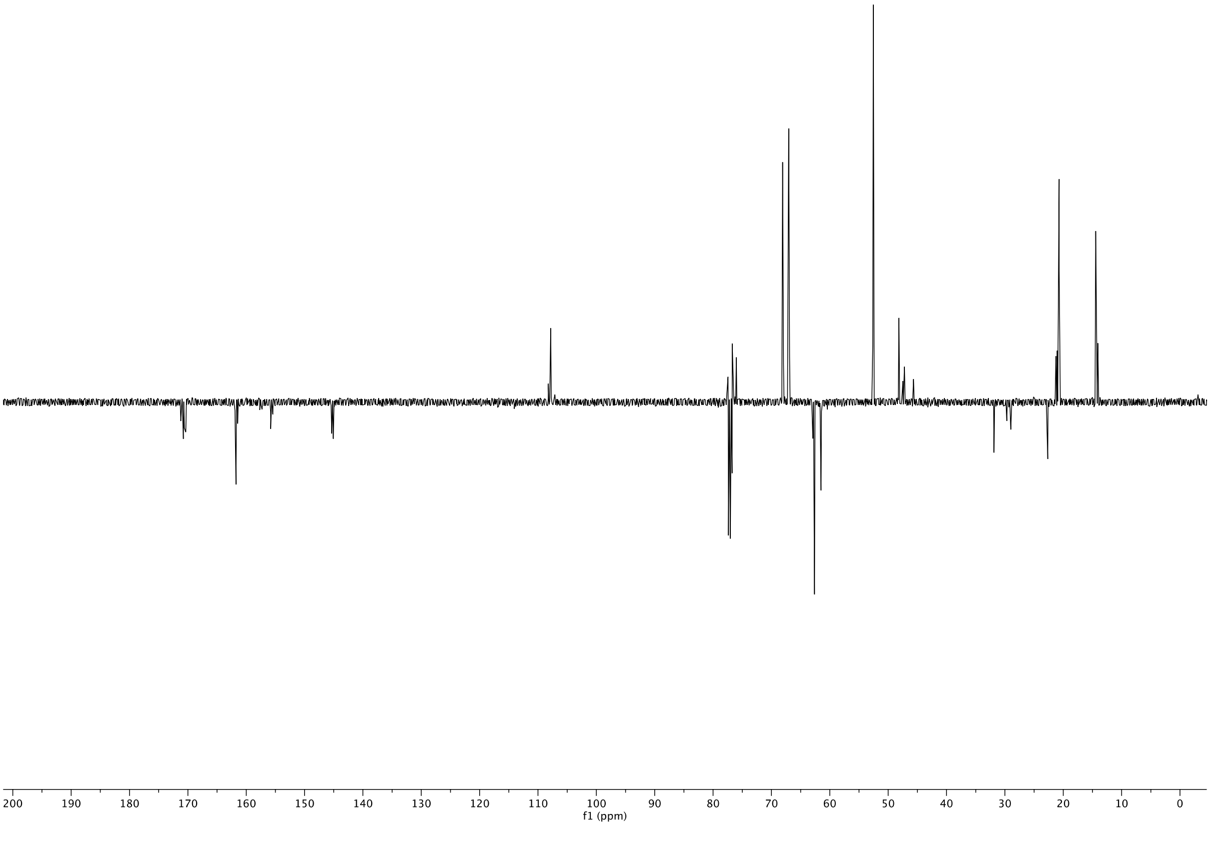

Methyl (5-acetamido-2,4,7,8-tetra-*O*-acetyl-3-fluoro-3,5-dideoxy-5-D-galacto)onate (16)

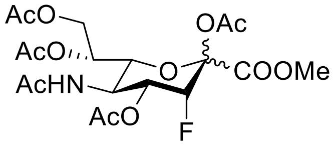

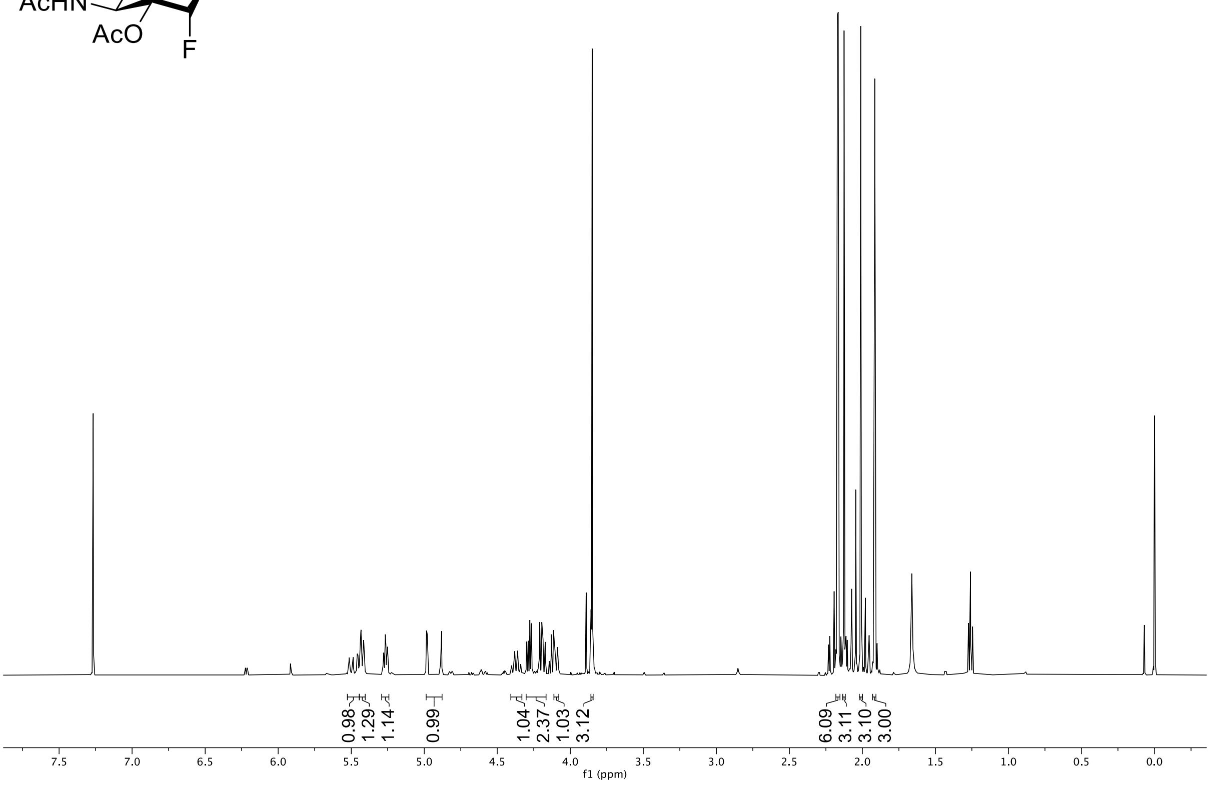

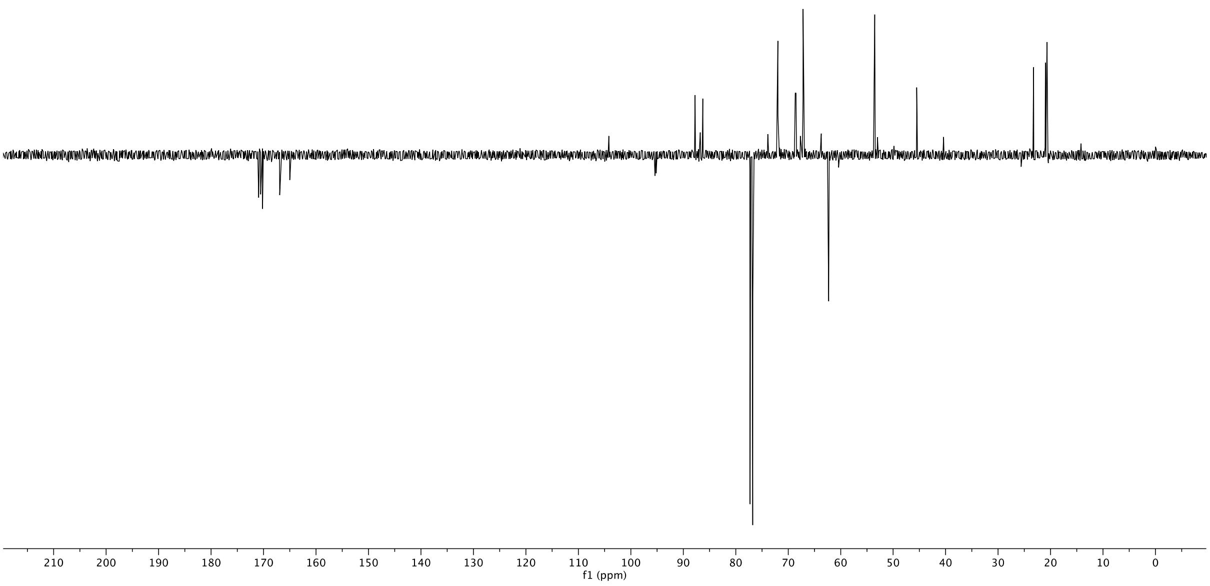

Methyl (5-ethylcarbamado-2,4,7,8-tetra-*O*-acetyl-3-fluoro-3,5-dideoxy-5-D-galacto)onate (17)

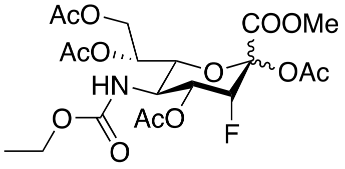

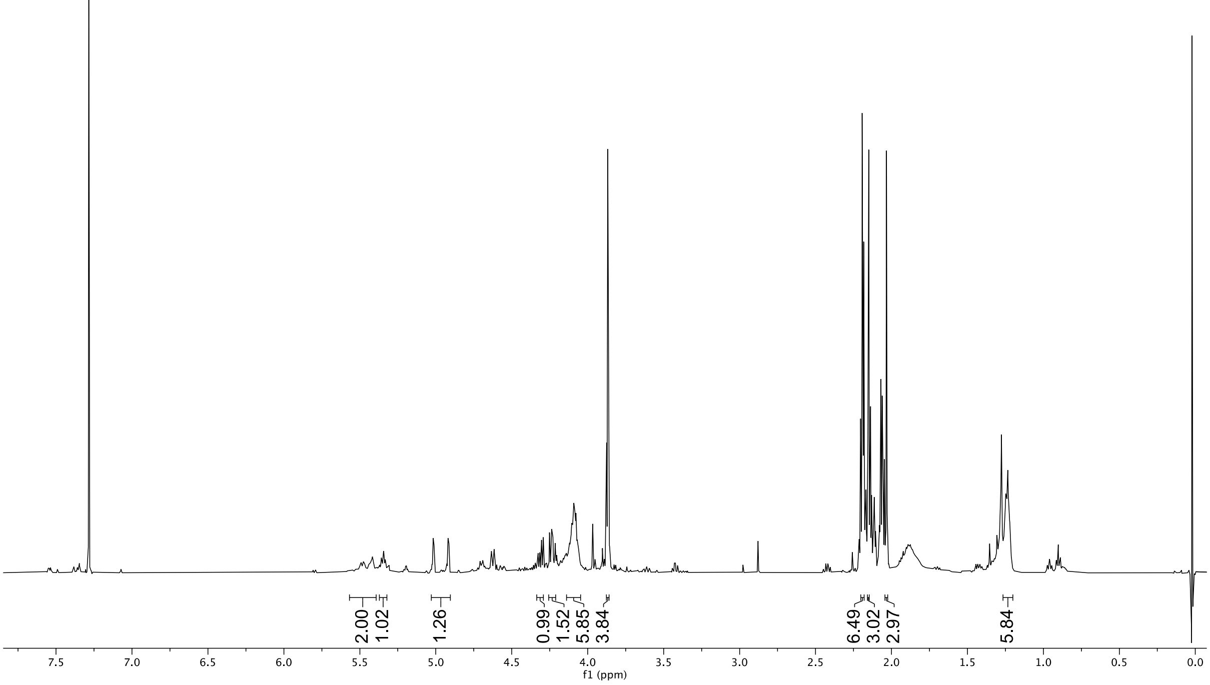

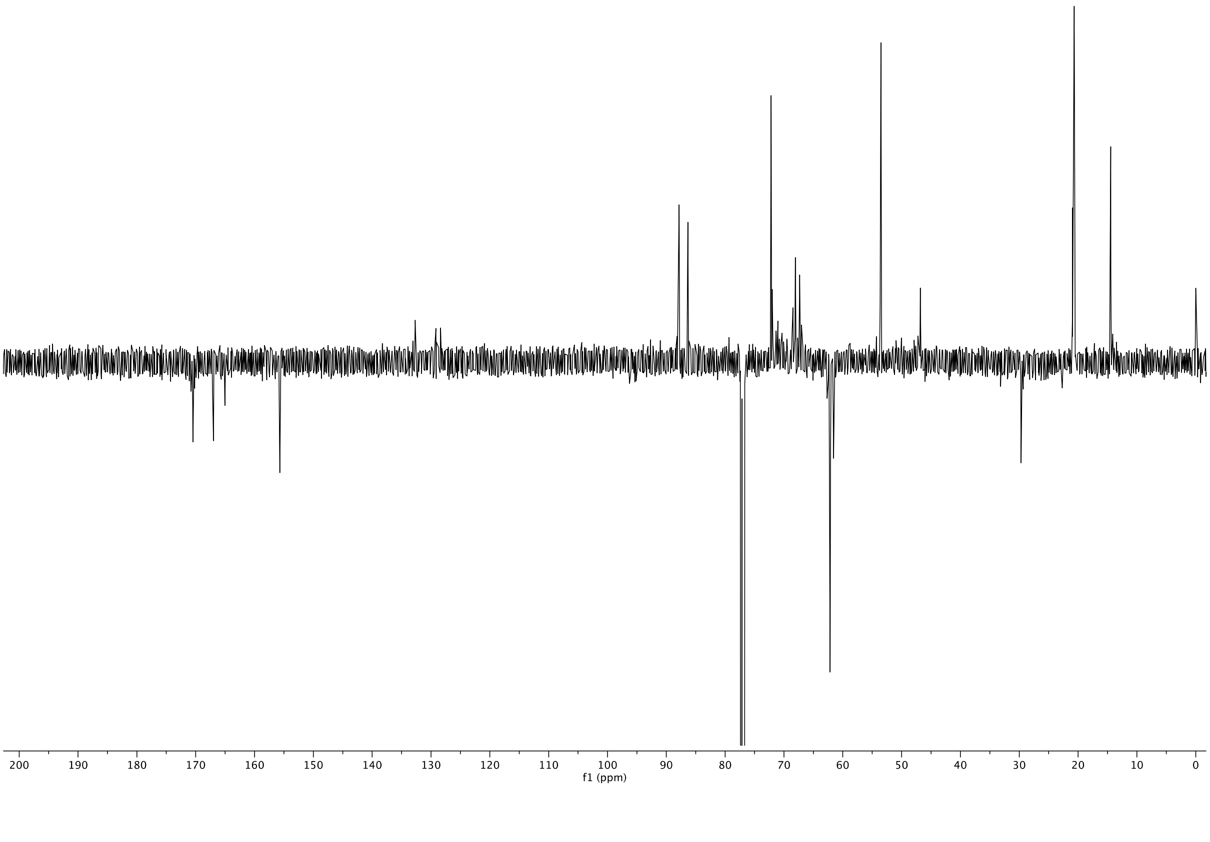

Methyl (5-acetemido-4,7-di-O-acetyl-3-dehyrdo-2,3,5-trideoxy-5-D-galacto)onate (18)

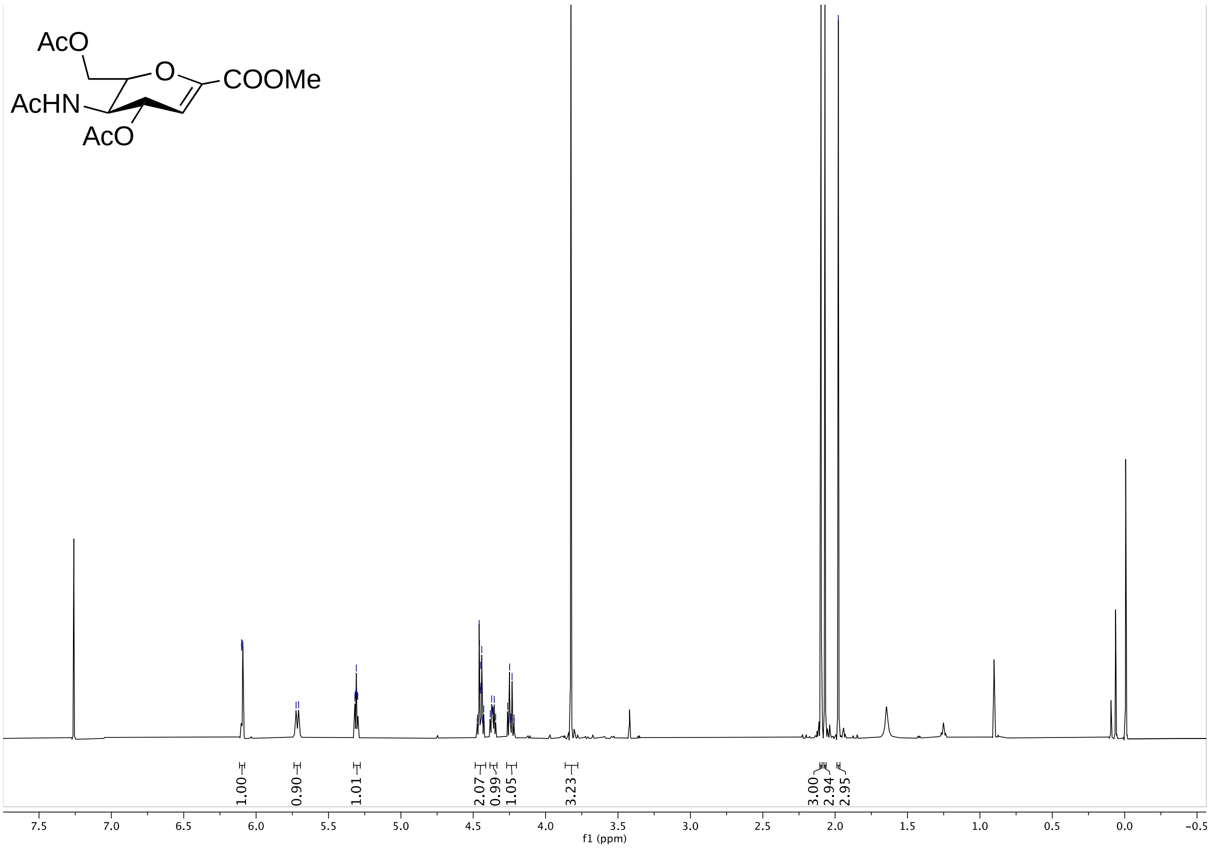

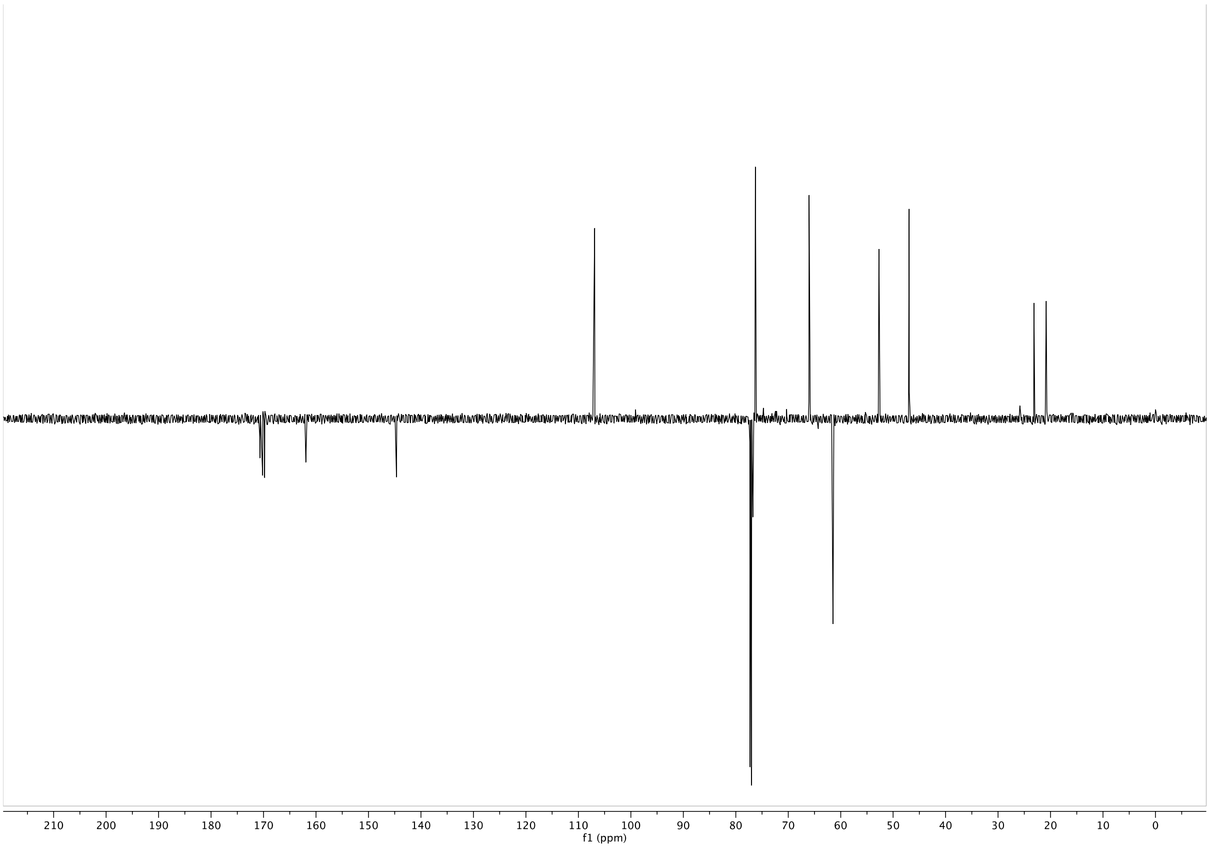

Methyl (5- ethylcarbamado-4,7-di-*O*-acetyl-3-dehyrdo-2,3,5 trideoxy-5-L-arabino)onate (19)

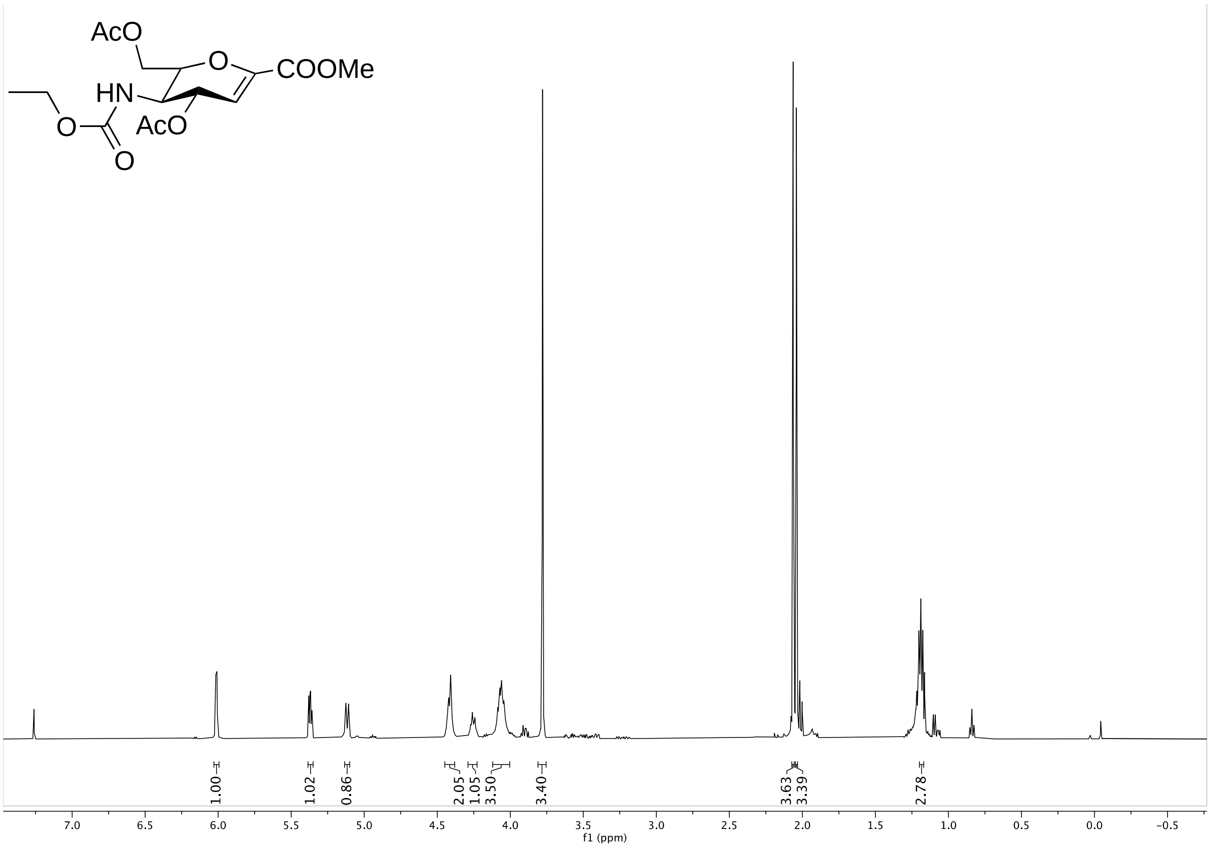

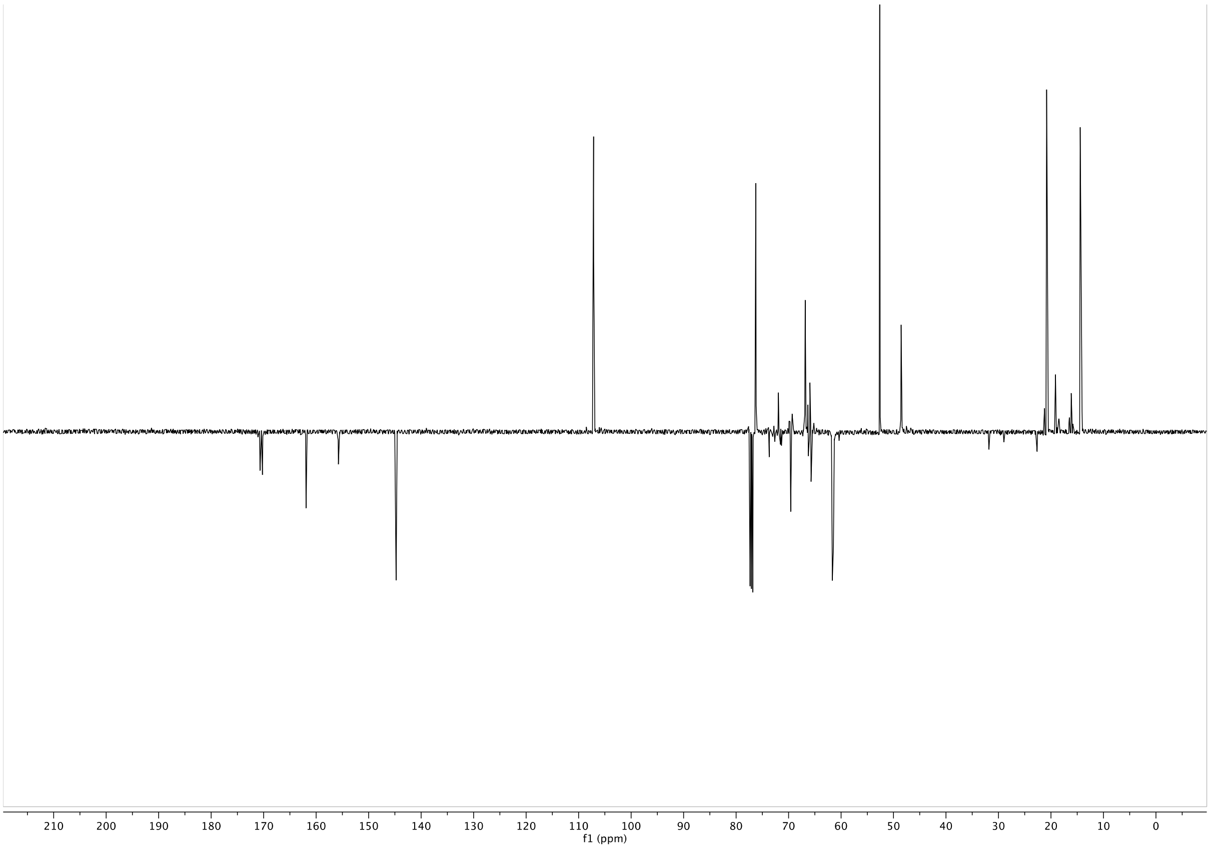

Methyl (5-acetamido-2,4,7-tri-*O*-acetyl-3-fluoro-3,5-dideoxy-5-L-arabino)onate (20)

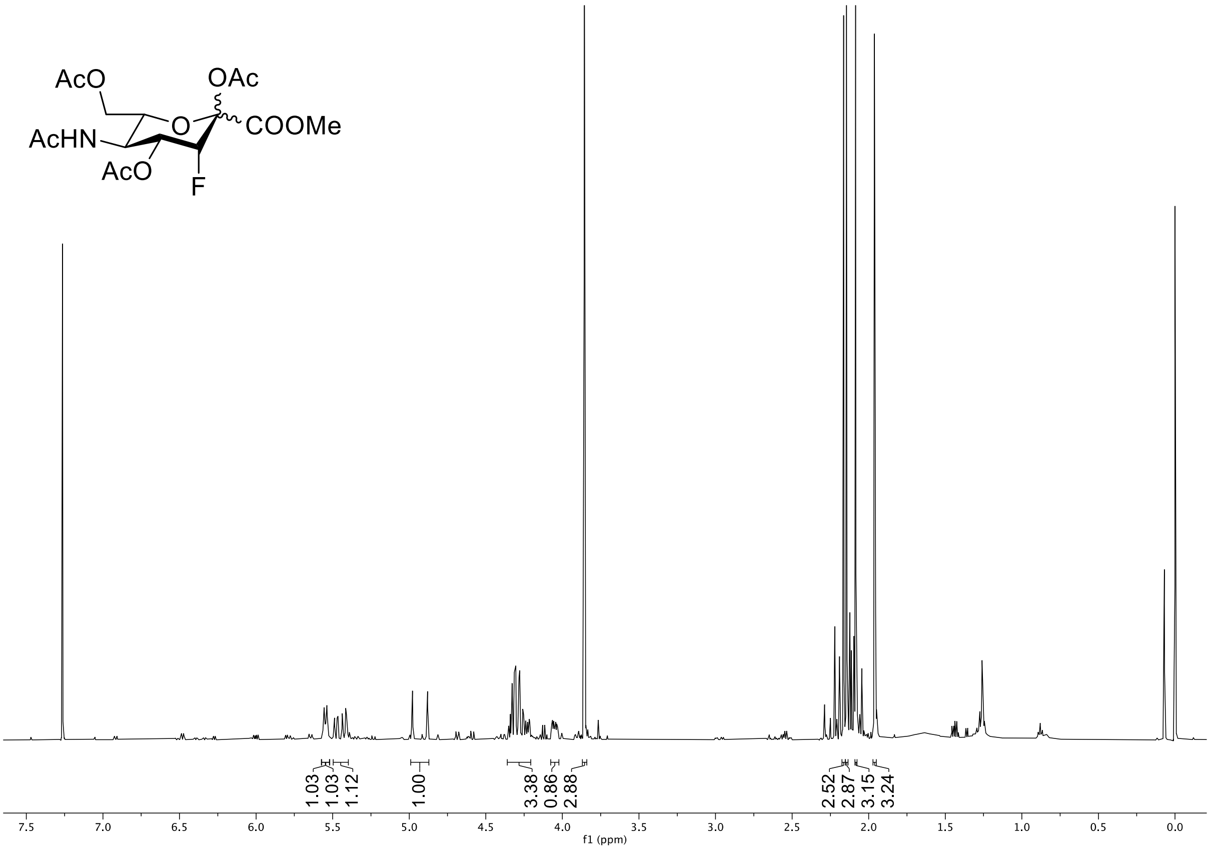

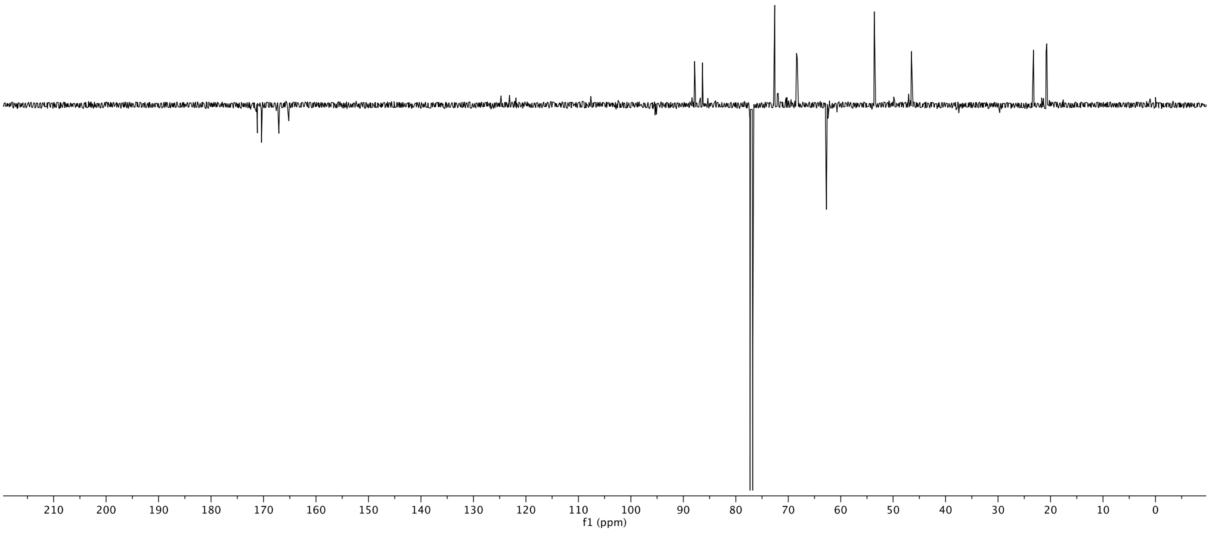

Methyl (5-ethylcarbamado-2,4,7-tri-*O*-acetyl-3-fluoro-3,5-dideoxy-5-L-arabino)onate (21)

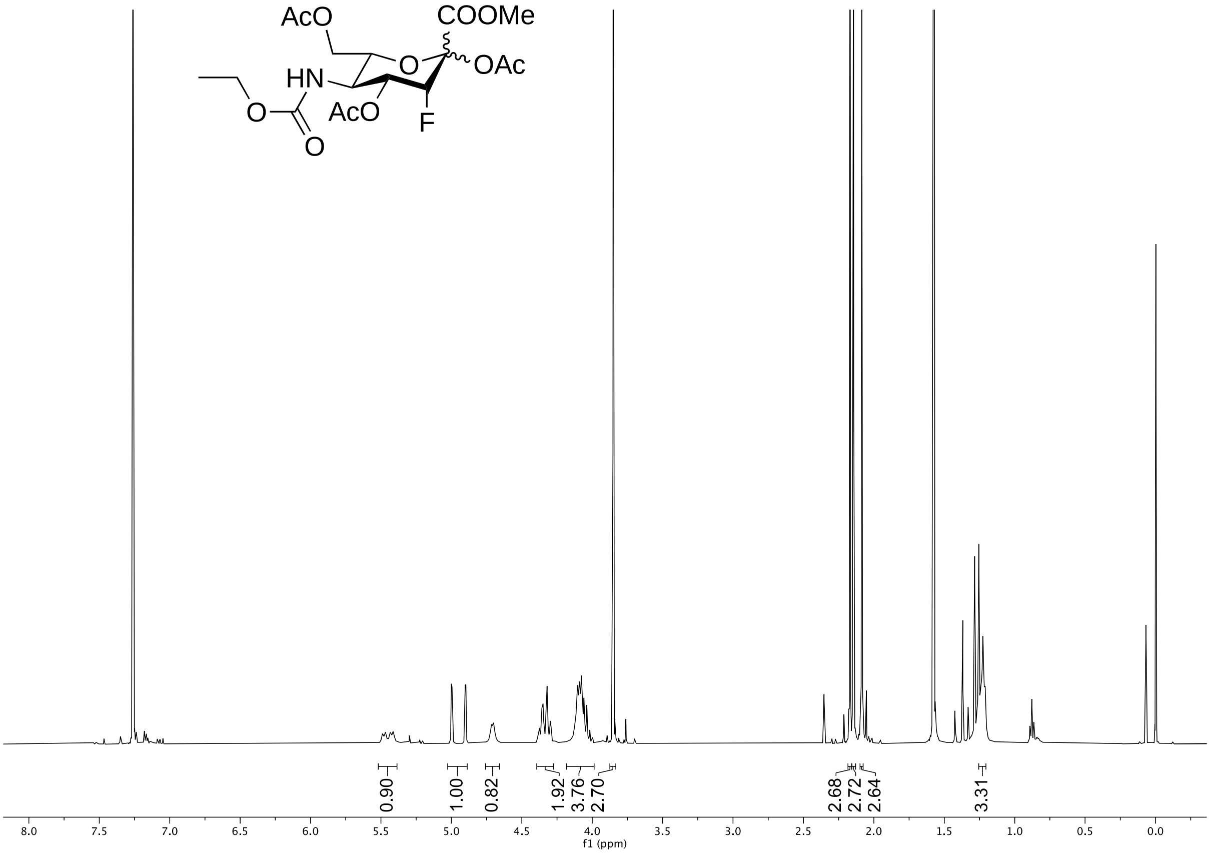

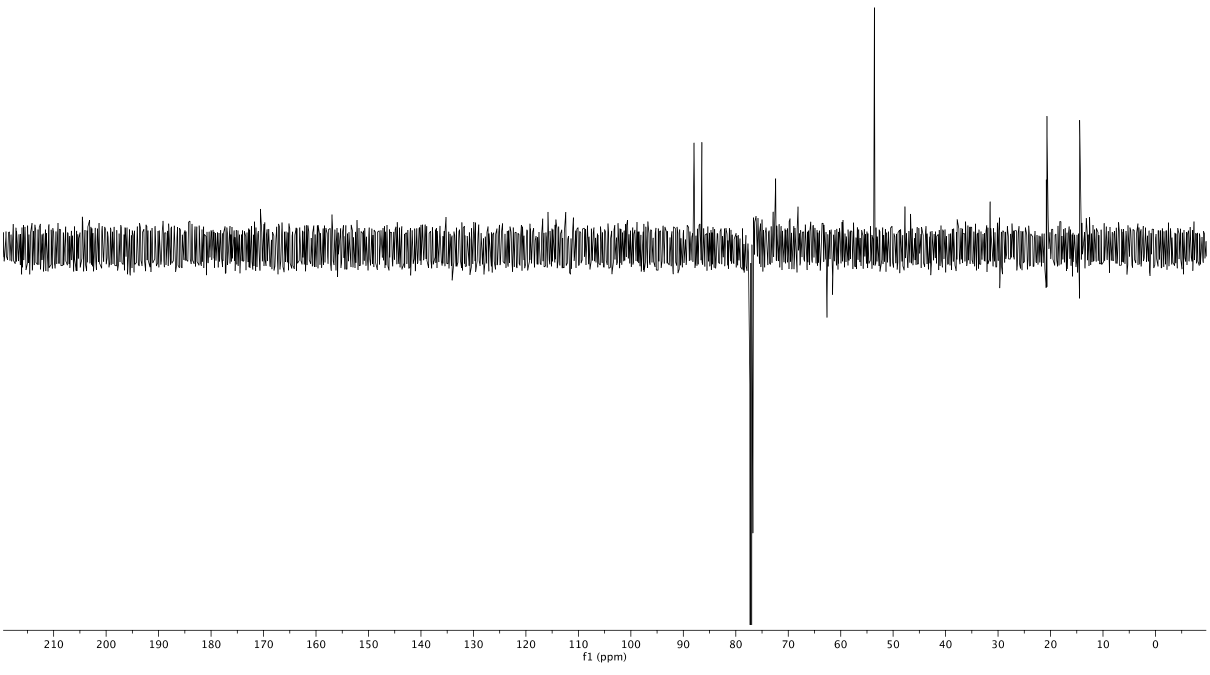

Methyl (5-acetamido-4-azido-7,8,9-tri-O-acetyl-3-dehydro-3,5-dideoxy-3-fluoro-5-β-D-glycero-D-galacto)onate (23)

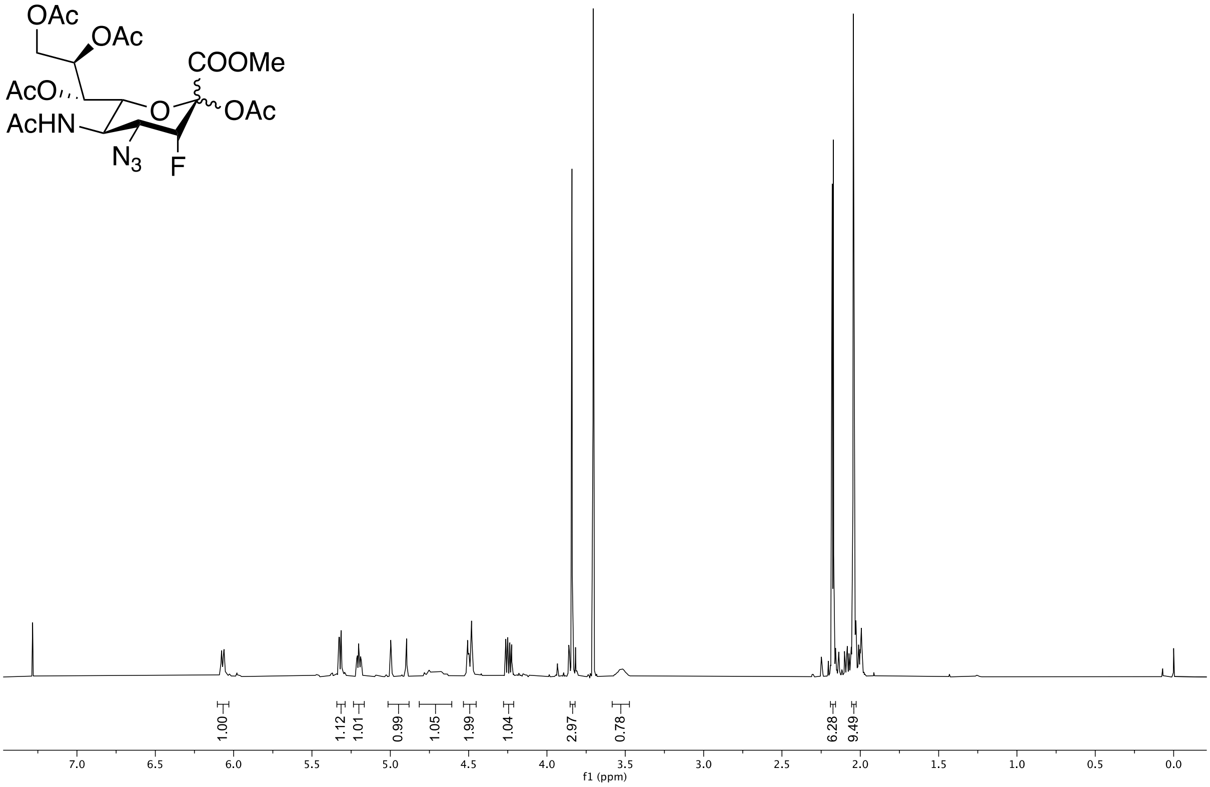

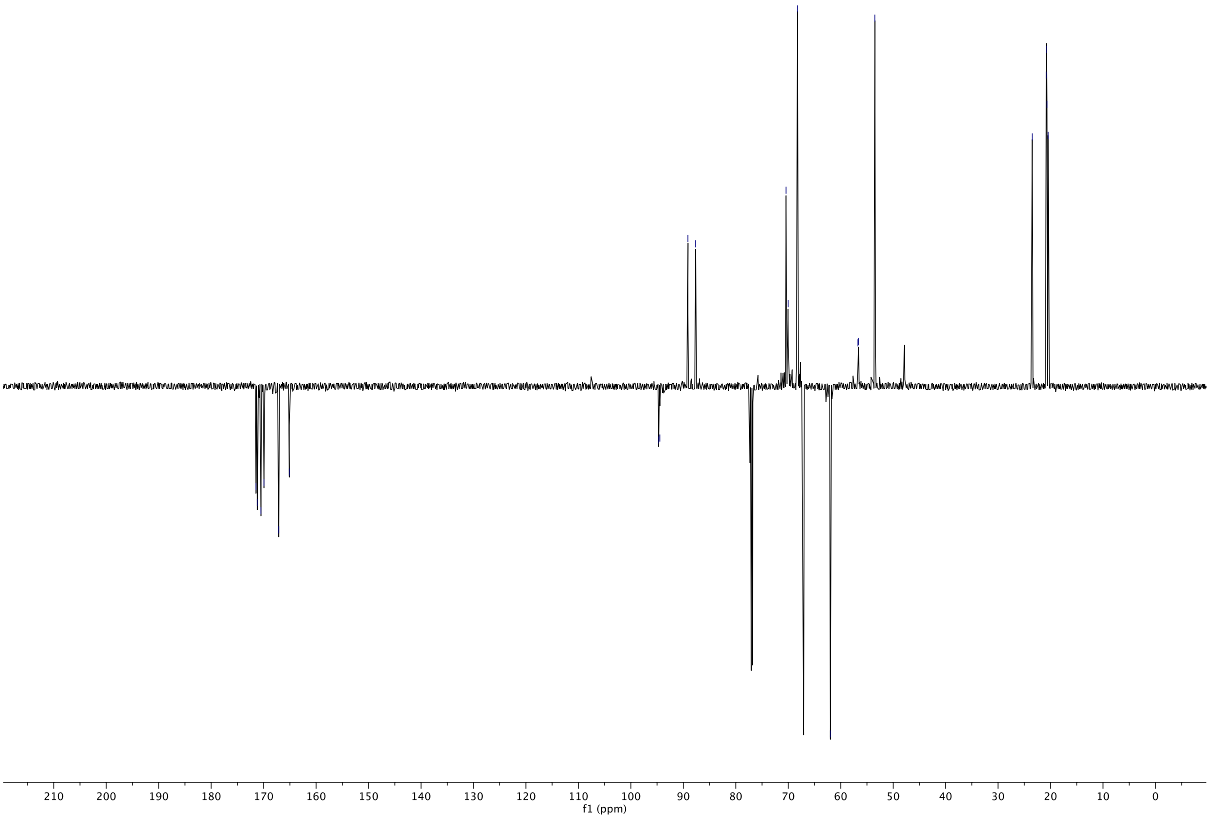

Methyl (5-ethylcarbamado-4-azido-7,8,9-tri-O-acetyl-3-dehydro-3,5-dideoxy-3-fluoro-5-β-D-glycero-D-galacto)onate (24)

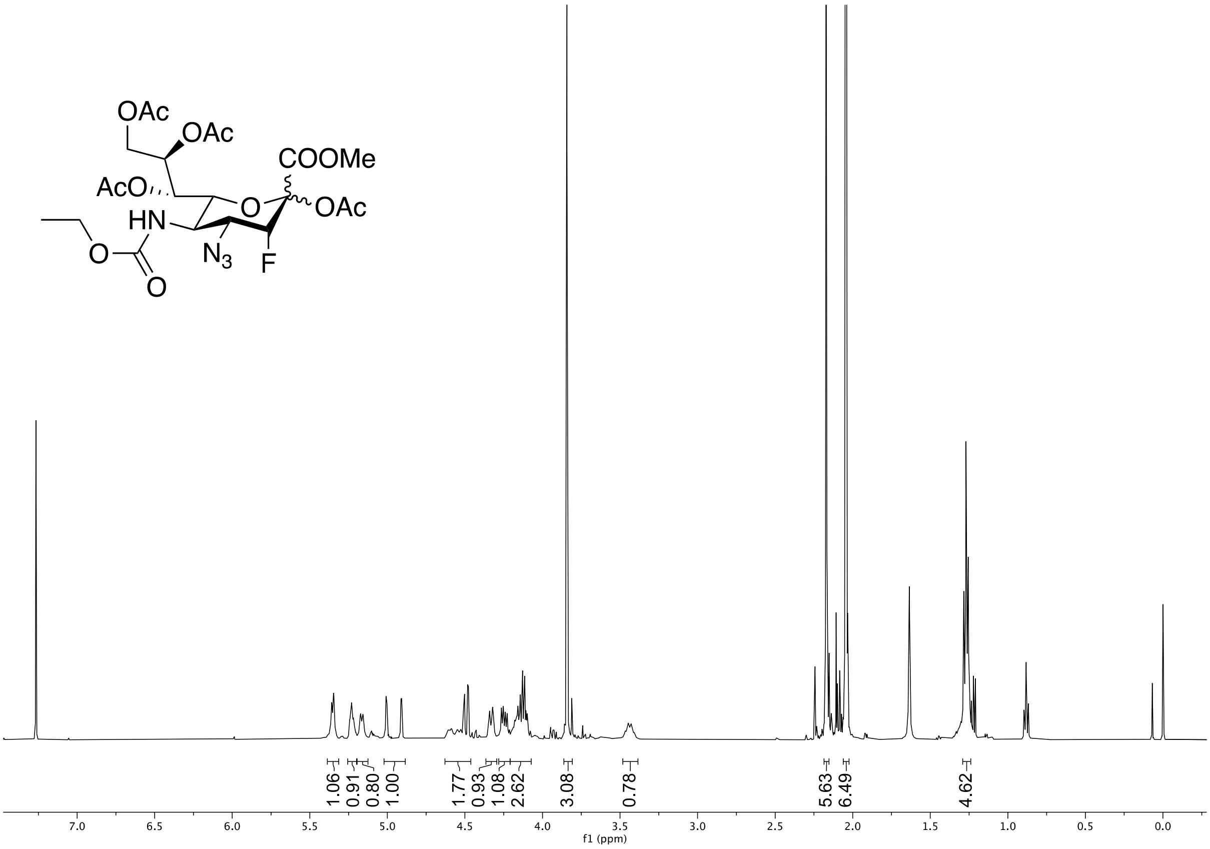

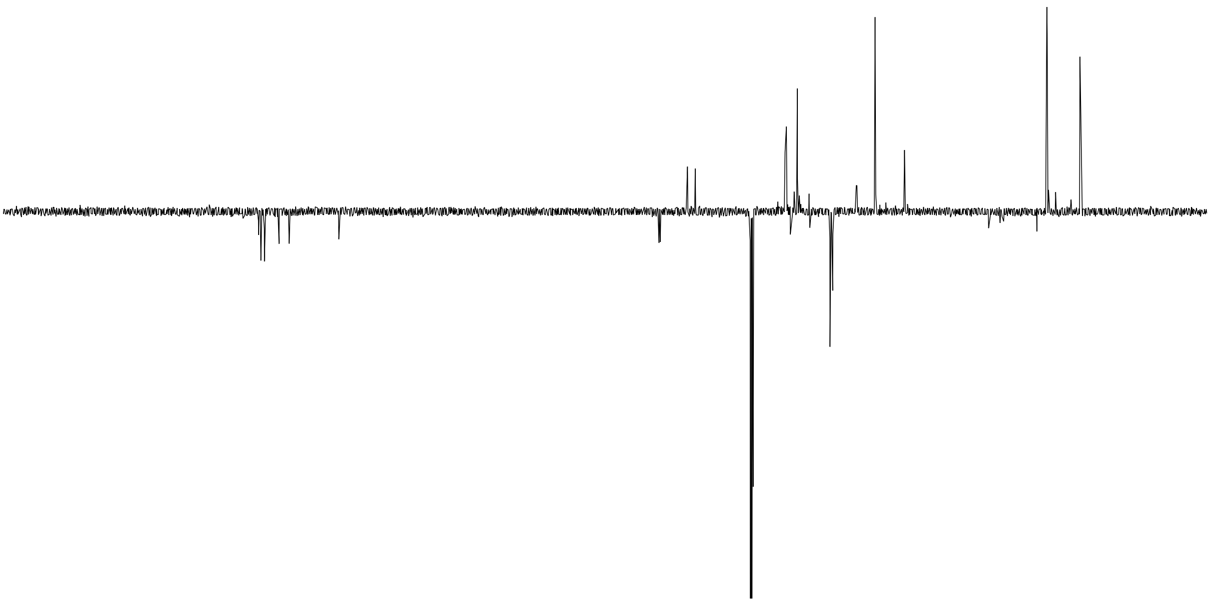

Methyl (5-acetamido-4-propargylcarbamado-7,8,9-tri-O-acetyl-3-dehydro-3,5-dideoxy-3-fluoro-5-β-D-glycero-D-galacto)onate (25)

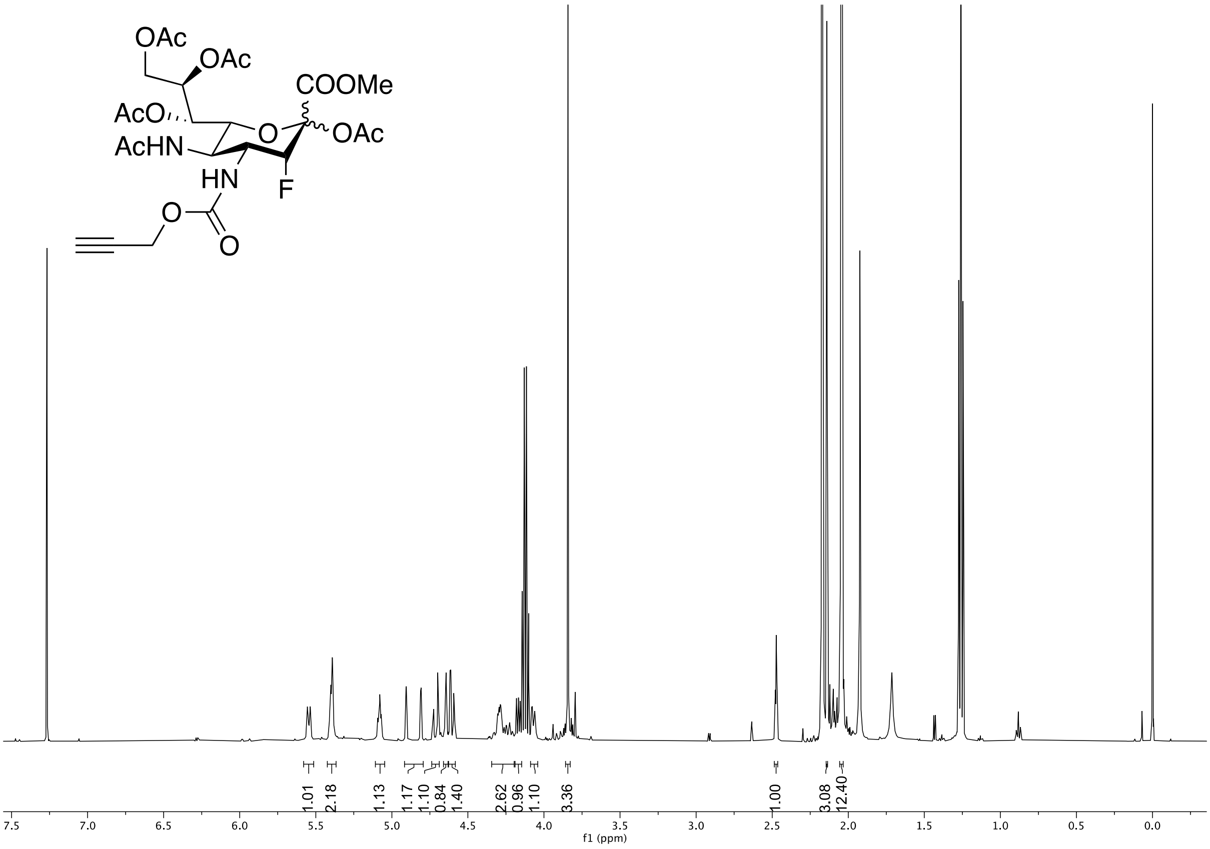

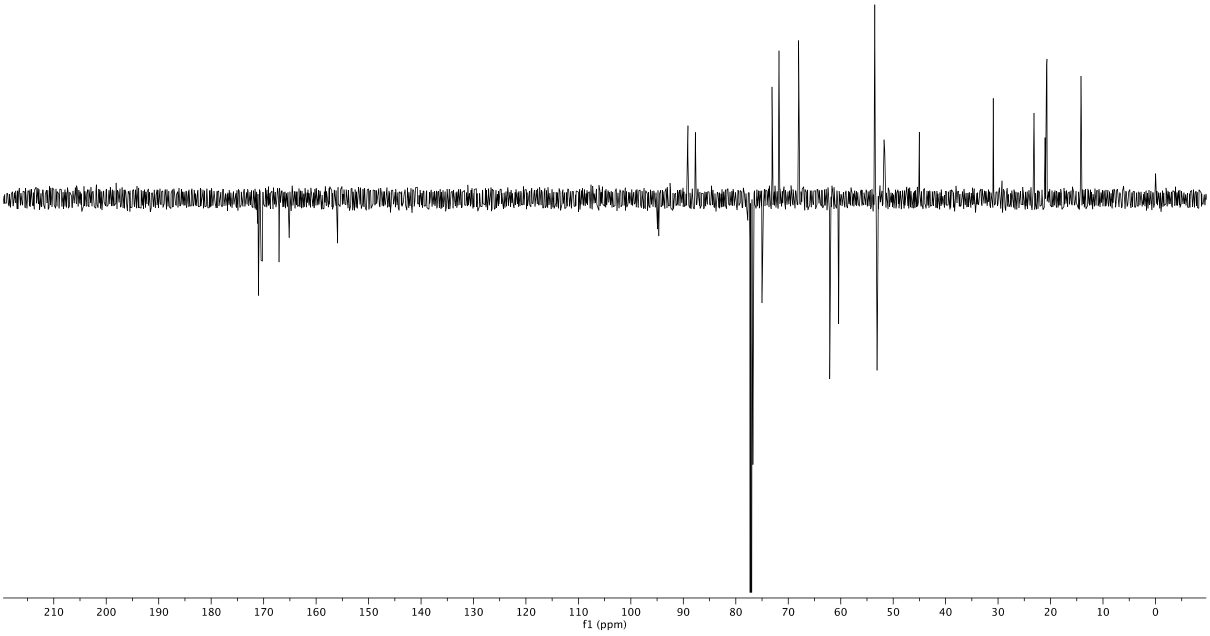

Methyl (5-*N*-4-pentynoyl-2,4,7,8,9-penta-*O*-acetyl-3,5-dideoxy-D-glycero-β-D-galacto-non-2-ulopyranoside)onate (26), mixture of anomers

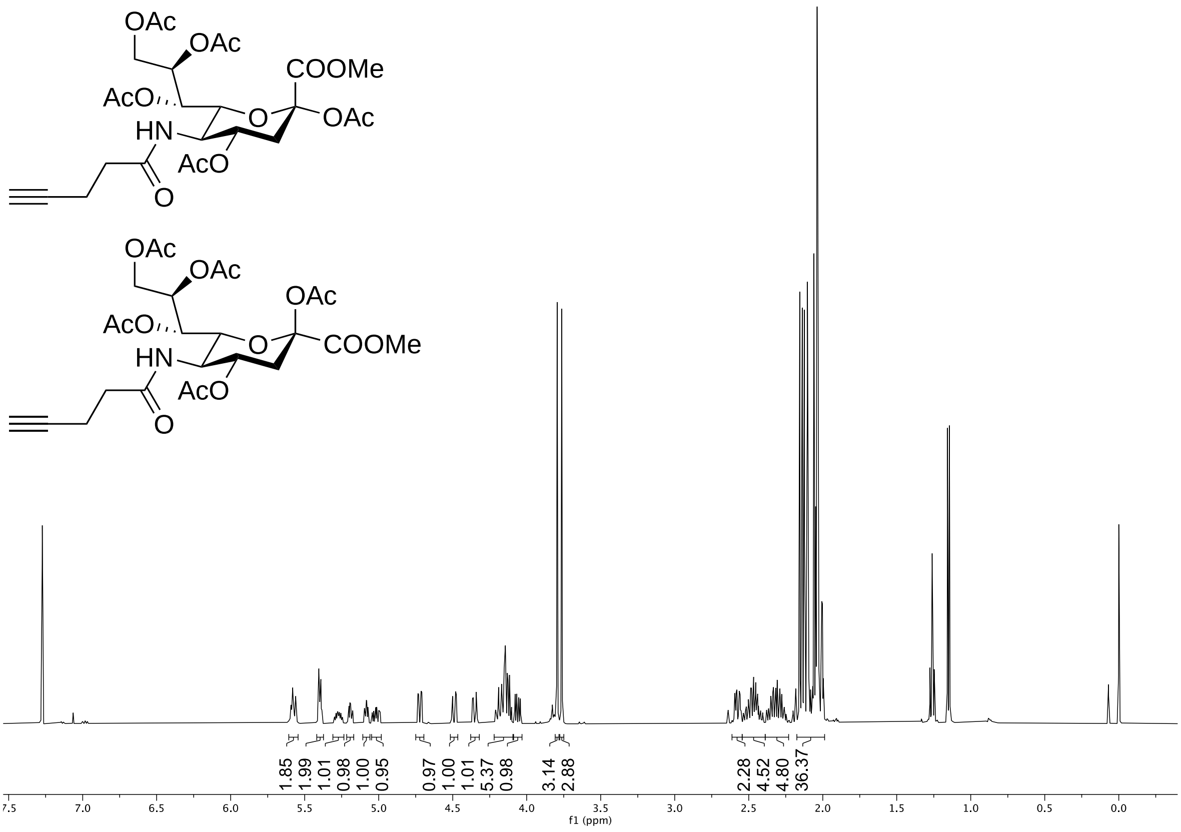

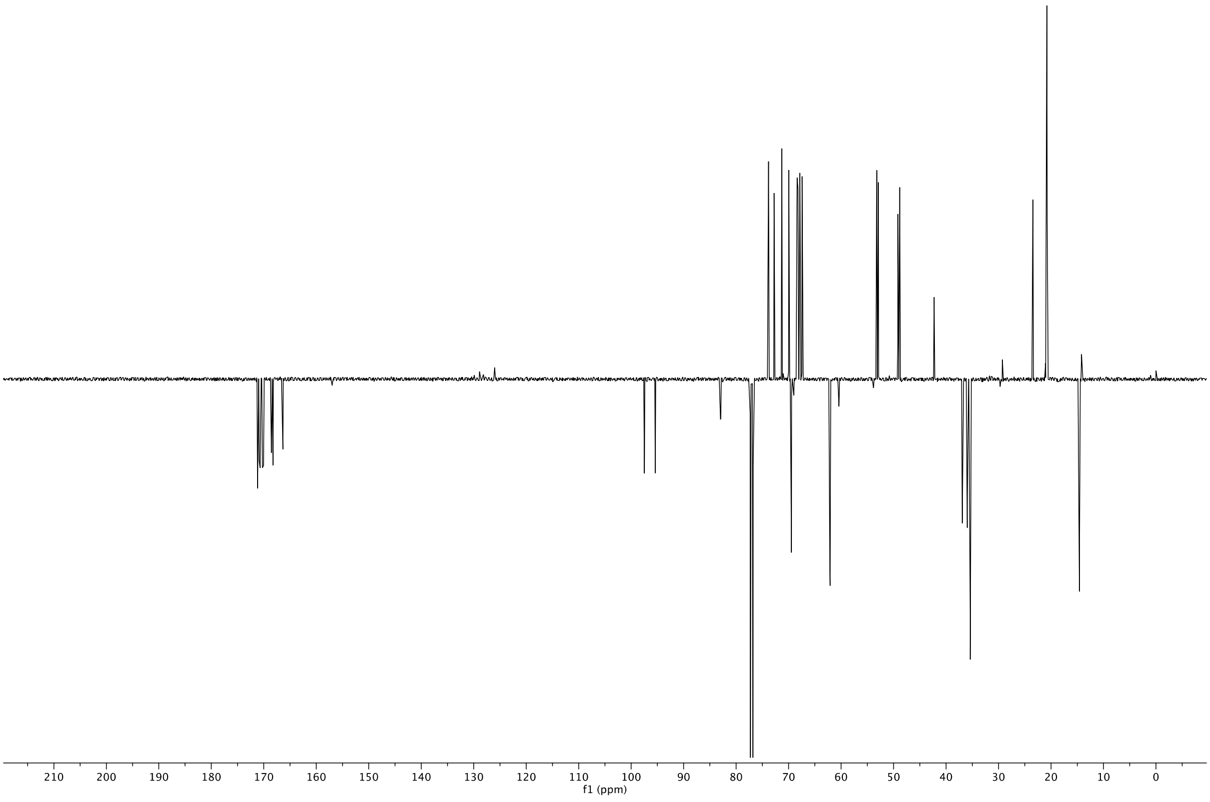

Methyl (5-*N*-propargylaminocarbonyl-2,4,7,8,9-penta-*O*-acetyl-3,5-dideoxy-D-glycero-β-D-galacto-non-2-ulopyranoside)onate (28)

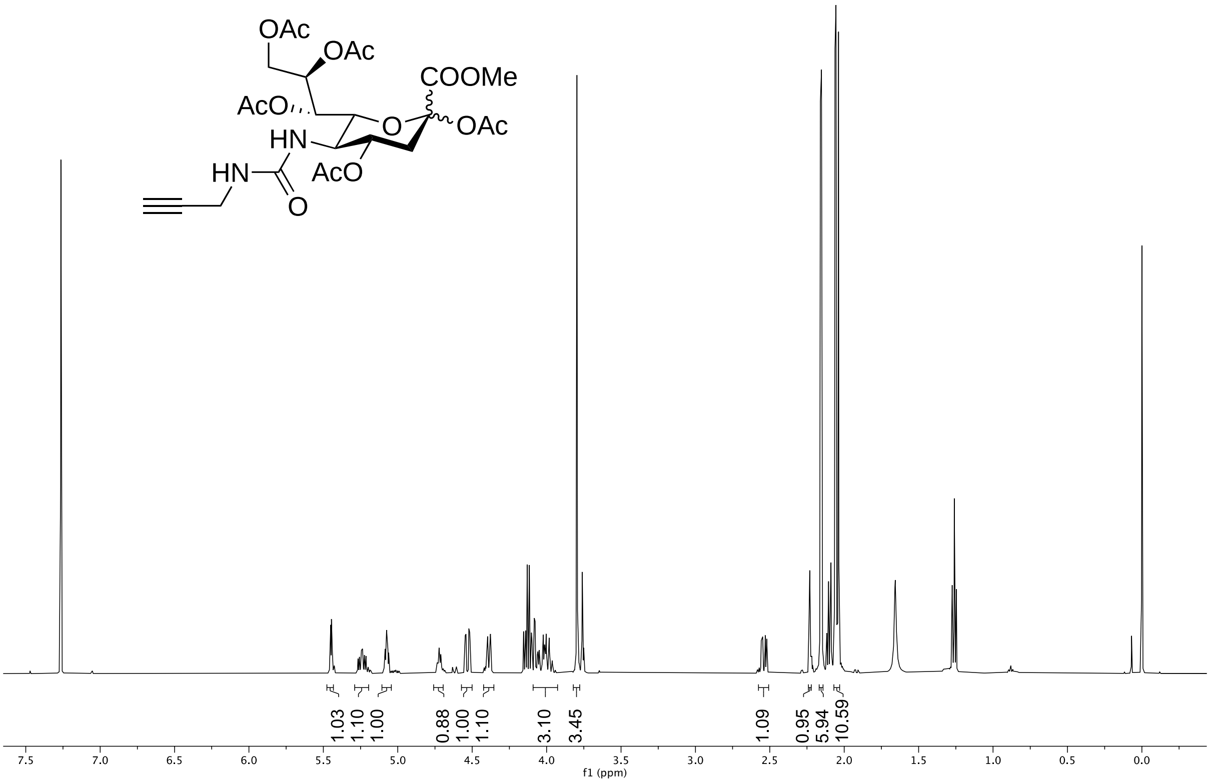

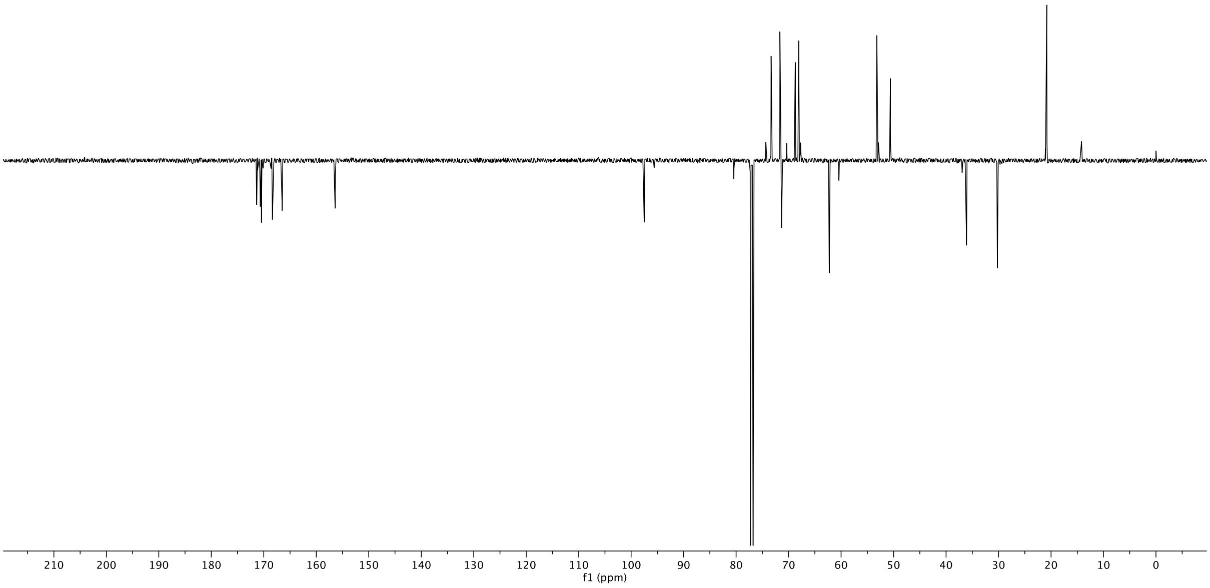

References:

(1) Heise, T., Pijnenborg, J. F., Büll, C., van Hilten, N., Kers-Rebel, E. D., Balneger, N., Elferink, H., Adema, G. J., and Boltje, T. J. (2018) Potent metabolic sialylation inhibitors based on C-5-modified fluorinated sialic acids. *J. Med. Chem. 62*, 1014-1021.

(2) Büll, C., Heise, T., Beurskens, D. l. M., Riemersma, M., Ashikov, A., Rutjes, F. P., van Kuppevelt, T. H., Lefeber, D. J., den Brok, M. H., and Adema, G. J. (2015) Sialic acid glycoengineering using an unnatural sialic acid for the detection of sialoglycan biosynthesis defects and on-cell synthesis of siglec ligands. *ACS chemical biology 10*, 2353-2363.

(3) Isidro-Llobet, A., Georgiou, K. H., Galloway, W. R., Giacomini, E., Hansen, M. R., Méndez-Abt, G., Tan, Y. S., Carro, L., Sore, H. F., and Spring, D. R. (2015) A diversity-oriented synthesis strategy enabling the combinatorial-type variation of macrocyclic peptidomimetic scaffolds. *Organic & biomolecular chemistry 13*, 4570-4580.
